# Functional Heterogeneity within the Developing Zebrafish Epicardium

**DOI:** 10.1016/j.devcel.2020.01.023

**Published:** 2020-03-09

**Authors:** Michael Weinberger, Filipa C. Simões, Roger Patient, Tatjana Sauka-Spengler, Paul R. Riley

**Affiliations:** 1 Department of Physiology, Anatomy and Genetics, University of Oxford, Oxford, Oxfordshire OX1 3PT, UK; 2 MRC Weatherall Institute of Molecular Medicine, Radcliffe Department of Medicine, University of Oxford, Oxford, Oxfordshire OX3 9DS, UK

**Keywords:** heterogeneity, epicardium, zebrafish, heart, single cell, RNA-seq, development

## Abstract

The epicardium is essential during cardiac development, homeostasis, and repair, and yet fundamental insights into its underlying cell biology, notably epicardium formation, lineage heterogeneity, and functional cross-talk with other cell types in the heart, are currently lacking. In this study, we investigated epicardial heterogeneity and the functional diversity of discrete epicardial subpopulations in the developing zebrafish heart. Single-cell RNA sequencing uncovered three epicardial subpopulations with specific genetic programs and distinctive spatial distribution. Perturbation of unique gene signatures uncovered specific functions associated with each subpopulation and established epicardial roles in cell adhesion, migration, and chemotaxis as a mechanism for recruitment of leukocytes into the heart. Understanding which mechanisms epicardial cells employ to establish a functional epicardium and how they communicate with other cardiovascular cell types during development will bring us closer to repairing cellular relationships that are disrupted during cardiovascular disease.

## Highlights

• scRNA-seq uncovered 3 developmental epicardial subpopulations (Epi1-3) in the zebrafish
• Epi1-specific gene, *tgm2b*, regulates the cell numbers in the main epicardial sheet
• Epi2-specific gene, *sema3fb*, restricts the number of *tbx18*^+^ cells in the cardiac outflow tract
• Epi3-specific gene, *cxcl12a*, guides *ptprc*/*CD45*^+^ myeloid cells to the developing heart

## Graphical Abstract

**Figure:**
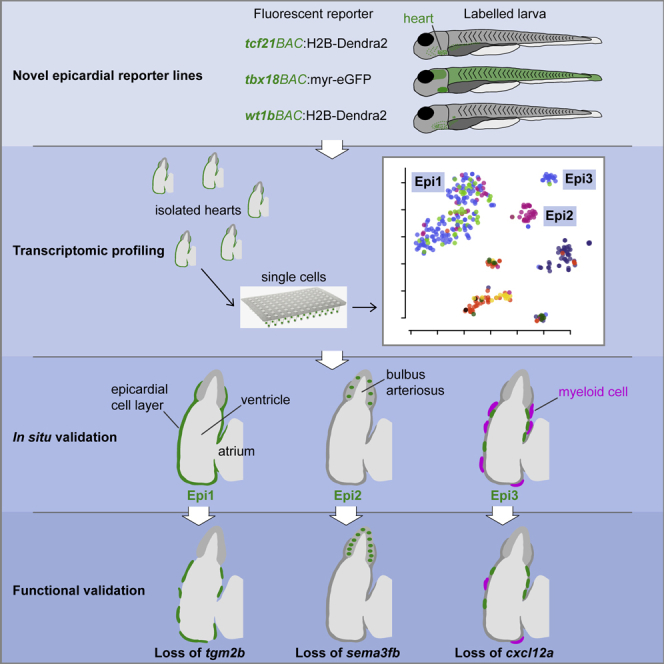

## Teaser

Weinberger et al. identify three cell subpopulations with unique gene expression signatures and patterns in the developing zebrafish epicardium. These epicardial subpopulations fulfill distinct functions in the formation of the epicardial cell layer, the development of the cardiac outflow tract, and the guidance of myeloid cells into the heart.

## Introduction

During embryonic development, numerous cardiovascular cell types interact to build and maintain a functional heart. The epicardium is a mesothelial cell sheet that covers the heart’s outer surface and, together with the myocardium and the endocardium, forms the wall of the heart (reviewed in Simões and Riley [2018]). The epicardium derives from a transient extracardiac structure called the proepicardium (PEO) (Männer et al., 2001, Peralta et al., 2013), which emerges at around 55 h post fertilization (hpf) in the zebrafish embryo. Between 55 hpf and 72 hpf, proepicardial cells translocate to the ventricle via pericardial fluid movements, where they form a continuous cell layer (Peralta et al., 2013). Subsequently, a subset of epicardial cells undergoes an epithelial-to-mesenchymal transition, giving rise to epicardium-derived cells (EPDCs) (von Gise and Pu, 2012). These delaminating cells invade the subepicardial compartment and colonize the underlying myocardium to nurture the further growth of the developing heart muscle and coronary vessels by acting as an essential source of mitogens (Pérez-Pomares and de La Pompa, 2011).

In addition to providing signals, EPDCs can directly give rise to many of the cell types that form the developing heart. Studies have reported an epicardial contribution to adipose tissue (Chau et al., 2014, Liu et al., 2014, Yamaguchi et al., 2015), vascular smooth muscle cells, necessary for vascular support and proper coronary formation, and cardiac fibroblasts in chick (Dettman et al., 1998, Männer, 1999, Mikawa and Gourdie, 1996, Pérez-Pomares et al., 1997), mouse (Acharya et al., 2012, Swonger et al., 2016, Wessels et al., 2012, Wu et al., 2013, Zhou et al., 2010), and zebrafish (Kikuchi et al., 2011). A much less consensual view of EPDC fate exists concerning their putative differentiation into endothelial cells (Dettman et al., 1998, Mikawa and Fischman, 1992, Pérez-Pomares et al., 2002, Pérez-Pomares et al., 1998, Zhou et al., 2008) and cardiomyocytes (Cai et al., 2008, del Monte et al., 2011, Guadix et al., 2006, Ruiz-Villalba et al., 2013, Zhou et al., 2008). These findings have relied heavily on tissue transplantation and Cre-based fate mapping analyses, which have limitations and may confound interpretation of results because of issues with activation of the Cre drivers in lineage derivatives and mosaic or ectopic expression of reporters (Davis et al., 2012).

Although there has been significant progress understanding the biology of the epicardium, it is still not clear whether pre-migratory EPDCs are a homogeneous source of multipotent progenitors or become specified as epicardial subpopulations already within the epicardium proper. In the developing mouse and chick heart, the epicardial expression of *Tcf21*, *Wt1*, and *Tbx18* is restricted to subsets of cells (Braitsch et al., 2012), establishing a precedent for cellular heterogeneity in the epicardium itself. This is supported by a recent study showing conserved heterogeneity within epicardium derived from human pluripotent stem cells (Gambardella et al., 2019). Epicardial heterogeneity might be rooted in the fact that multiple tissues contribute to this structure. Although most zebrafish ventricular epicardial cells originate from the PEO, the epicardium covering the bulbus arteriosus (BA) was found to originate from the pericardial sac (Peralta et al., 2014, Pérez-Pomares et al., 2003). Additionally, the PEO itself was shown to be heterogeneous (Plavicki et al., 2014). A subset of murine proepicardial cells that expresses the transcription factor Scleraxis (Scx) and the chemokine Semaphorin 3D (Sema3D) gives rise to the endocardium and coronary endothelium (Katz et al., 2012). Most of these cells do not express Wt1 or Tbx18.

However, previous insights into epicardial heterogeneity have remained limited and restricted to a small number of epicardial markers. To gain unbiased insight into epicardial cell heterogeneity, we characterized the developmental transcriptome of the zebrafish epicardium at a single-cell level, combining confocal microscopy of newly generated epicardial reporter lines and single-cell transcriptomics. We identified and functionally characterized three transcriptionally distinct epicardial cell subpopulations, only one of which (Epi1) contained cells co-expressing the bona fide epicardial signature genes *tcf21*, *tbx18*, and *wt1b* (Kikuchi et al., 2011, Serluca, 2008). Functional perturbation identified *tgm2b*, a transglutaminase gene highly enriched in Epi1, as necessary for the proper development of the epicardial cell sheet. The second subpopulation (Epi2), enriched in *tbx18* and smooth muscle markers such as *acta2* and *mylka*, was spatially localized outside the main epicardial layer, specifically in the smooth muscle of the BA. Loss of the chemokine semaphorin *3fb* (*sema3fb*), highly enriched in Epi2, increased the number of *tbx18*^*+*^ cells in the BA, revealing that *sema3fb* controlled the spatiotemporal access of epicardial cells to the outflow tract. The third subpopulation (Epi3) was highly enriched for cell guidance cues such as *cxcl12a*. Loss of this chemokine decreased the number of *ptprc/CD45*^+^ leukocytes on the epicardial surface, unraveling the importance of Epi3 in establishing intercellular communications responsible for the recruitment and/or retention of *ptprc/CD45*^+^ hematopoietic cells within the developing heart.

Building on the understanding of the genetic control of the developing epicardium, our study defines the molecular signatures and functional roles of specific epicardial cell subpopulations, thus providing insights into the specific programs within which the epicardium functions during cardiac development.

## Results

### Expression of *tcf21*, *tbx18*, and *wt1b* Is Heterogeneous in the Developing Zebrafish Epicardium

**Figure 1:**
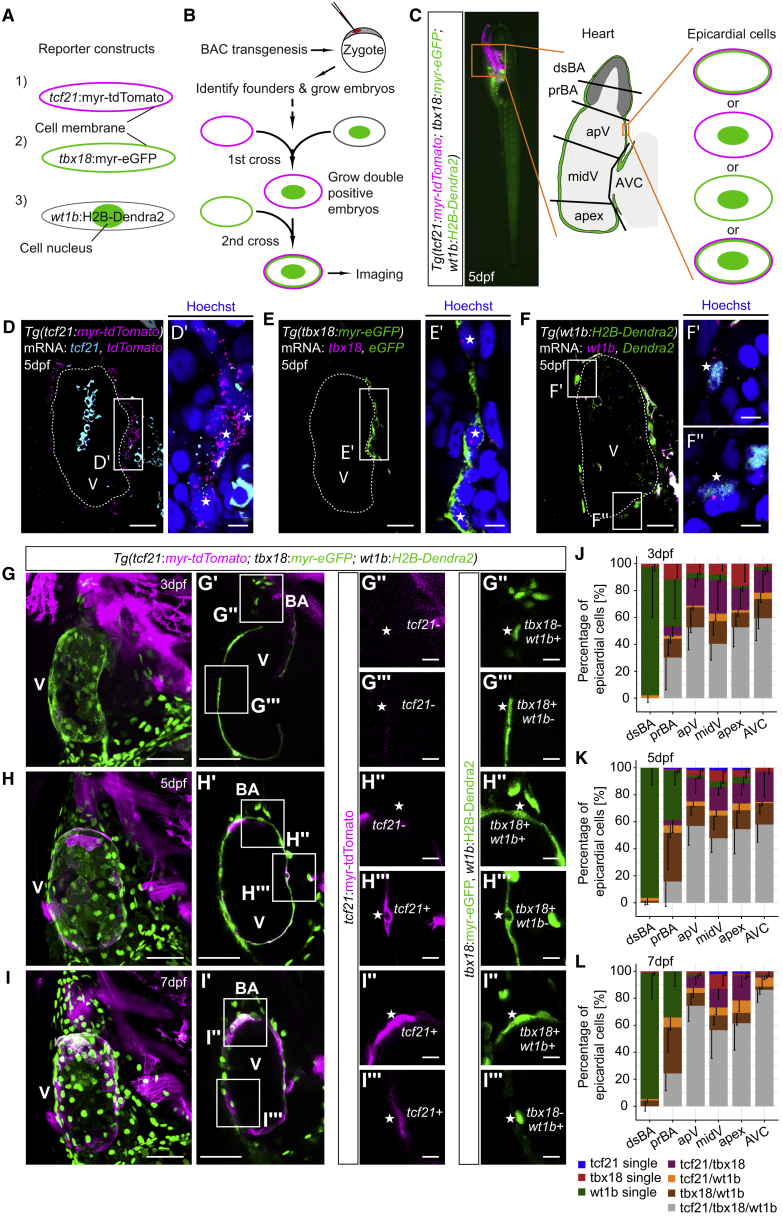
Heterogeneous Expression of *tcf21*, *tbx18*, and *wt1b* in the Developing Zebrafish Epicardium (A) Fluorescence of *tcf21*:myr-tdTomato (magenta membrane), *tbx18*:myr-eGFP (green membrane), and *wt1b*:H2B-Dendra2 (green nucleus). (B) Workflow to establish *TgBAC(tcf21:myr-tdTomato;tbx18:myr-eGFP;wt1b:H2B-Dendra2)*^*ox187*^. (C) Schematic of epicardial fluorescence patterns and cardiac regions analyzed. dsBA, distal bulbus arteriosus; prBA, proximal bulbus arteriosus; apV, arterial pole of the ventricle; and AVC, atrioventricular canal. (D–F) Single optical sections of mRNA stainings of (D) *tcf21* (cyan) and *tcf21*:myr-tdTomato (magenta), (E) *tbx18* (magenta) and *tbx18*:myr-eGFP (green), and (F) *wt1b* (magenta) and *wt1b*:H2B-Dendra2 (green). (D′–F′) Cell nuclei (asterisks) in the epicardial region surrounded by endogenous mRNA and fluorophore mRNA/protein. (G–I) Projections of the heart in triple reporter larvae at 3 dpf (G), 5 dpf (H), and 7 dpf (I). (G′ and H′) Single optical sections from (G) and (H). (I′) Short projection from (I). (G″–I″ and G‴–I‴) Representative epicardial fluorescence patterns. (J–L) Relative quantification of epicardial fluorescence patterns across the regions indicated in (C) at 3 dpf (J), 5 dpf (K), and 7 dpf (L). Scale bars: 20 μm in (D)–(F), 5 μm in (D′)–(F′), 50 μm in (G)–(I) and (G′)–(I′), and 10 μm in (G″)–(I″) and (G‴)–(I‴). Color channels were adjusted separately for brightness and contrast. Data in (J)–(L) are represented as mean minus standard deviation. Number of embryos analyzed: 3 dpf n = 6, 5 dpf n = 10, and 7 dpf n = 6. V, ventricle; BA, bulbus arteriosus. See also Figure S1.

Expression of *Tcf21, Tbx18*, and *Wt1* is restricted to subsets of epicardial cells in the developing mouse and chick heart (Braitsch et al., 2012). Similar heterogeneity is present in the zebrafish epicardium (González-Rosa et al., 2012, Kikuchi et al., 2011). However, the concurrent expression of all three epicardial genes has not been analyzed. Thus, we generated the zebrafish triple-reporter line *TgBAC(tcf21:myr-tdTomato;tbx18:myr-eGFP;wt1b:H2B-Dendra2)*^*ox187*^ (Figures 1A and 1B). Bacterial artificial chromosomes (BACs) contain large genomic fragments and recapitulate endogenous gene expression patterns more faithfully than promoter- or proximal enhancer-based transgenic lines (Bussmann and Schulte-Merker, 2011). In this line, membrane-tethered tdTomato and eGFP label *tcf21*^+^ and *tbx18*^+^ cells, respectively. Dendra2, localizing to the nucleus, identifies *wt1b*^+^ cells. The resulting combination of fluorescent signals enabled us to discriminate between epicardial cells that expressed combinations of *tcf21*, *tbx18*, and *wt1b* (Figure 1C). Whole-mount *in situ* hybridization chain reaction (HCR) (Choi et al., 2010, Choi et al., 2018) validated the newly generated BAC reporter lines (Figures 1D–1F, 1D′–1F′, and 1F″). Confocal imaging of triple-reporter larvae at 3 days post fertilization (dpf) (Figures 1G and 1G′), 5 dpf (Figures 1H, 1H′, and S1A–S1C), and 7 dpf (Figures 1I and 1I′) revealed that many epicardial cells did not express *tcf21*, *tbx18*, and *wt1b* simultaneously, but different subsets of the three markers (Figures 1G″–1I″ and 1G‴–1I‴). Interestingly, the distribution of these subsets differed across distinct morphological regions of the heart (Figures 1C and 1J–1L). For example, *wt1b*-only positive cells were mostly found on the BA, whereas *tcf21*/*tbx18*/*wt1b* triple-positive cells were mostly present on the ventricle. Furthermore, the relative number of triple-positive epicardial cells increased over time in all ventricular regions. However, in none of the cardiac regions did triple-positive cells become the only present epicardial subset. To validate these findings, we crossed the pre-existing reporter lines *TgBAC(tcf21:DsRed2)*^*pd37*^ and *TgBAC(tbx18:DsRed2)*^*pd22*^ (Kikuchi et al., 2011) to *Tg(wt1b:eGFP)*^*li1*^ (Perner et al., 2007) and observed clear heterogeneity in the *tcf21*/*wt1b* and *tbx18*/*wt1b* double-fluorescent settings (Figures S1D–S1K). We also observed epicardial heterogeneity at the endogenous gene expression level (Figure S1L). At 5 dpf, we detected nuclei adjacent to *myl7*^+^ myocardium that featured endogenous *tcf21*, *tbx18*, and *wt1b* transcripts (Figure 1L″, asterisk). However, we also detected *tcf21*^*+*^ and *wt1b*^+^ areas that lacked *tbx18* expression (Figure S1L‴, asterisk).

Overall, both newly generated BAC transgenic reporter lines and co-expression of endogenous transcripts demonstrate that the expression of *tcf21*, *tbx18*, and *wt1b* in the developing zebrafish epicardium is heterogeneous.

### Single-Cell Transcriptomic Profiling Identifies Distinct Cell Populations within the Developing Zebrafish Epicardium

**Figure 2:**
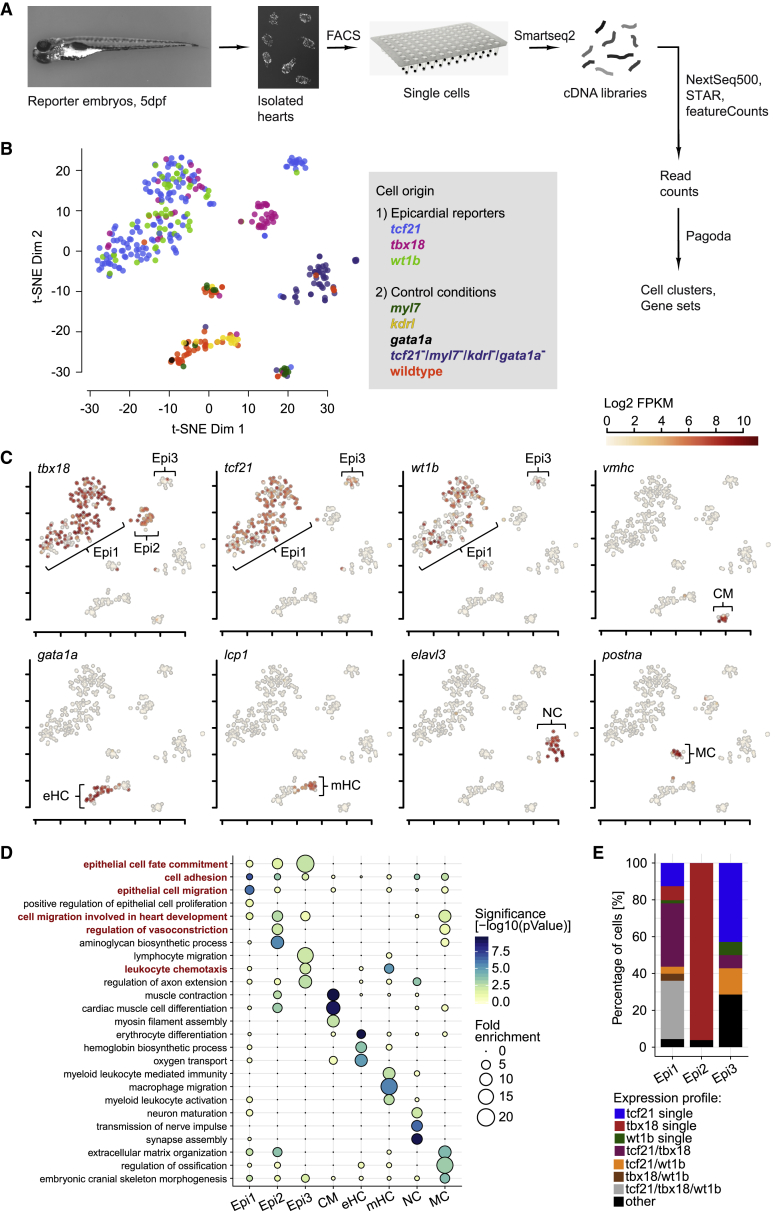
Single-Cell RNA Sequencing Reveals Distinct Epicardial Cell Clusters in the Developing Zebrafish Heart (A) Overview of the scRNA-seq workflow. (B) t-distributed stochastic neighbor embedding (t-SNE) clustering of single-cell samples. Colors indicate the reporter or WT line cells were isolated from. (C) Identification of single-cell clusters based on marker gene expression. Three clusters labeled by *tcf21*, *tbx18*, and *wt1b* are designated Epi1-3. Color key indicates range of log_2_ transformed fragments per kilobase of transcript per million reads mapped (FPKM) expression values. (D) GO term over-representation in the clusters (columns). Bubble size depicts fold enrichment and color scale depicts the statistical significance, with p-values calculated using Fisher’s exact test and Bonferroni correction for multiple hypothesis testing. GO terms important for further analysis are highlighted in red. (E) Relative quantification of the combinations of *tcf21*, *tbx18*, and *wt1b* expression in epi1–3 cells. CM, cardiomyocyte; eHC, erythroid hematopoietic cell; mHC, myeloid hematopoietic cell; NC, neural cell; and MC, mesenchymal cell. See also Figures S2 and S3.

To further investigate the observed cellular heterogeneity, we performed single-cell RNA sequencing (scRNA-seq) using Smart-seq2 approach (Picelli et al., 2013) and a NextSeq500 platform (Illumina) to obtain 75-bp paired-end sequencing reads of the generated libraries (see Figures S2A–S2F for quality control data). Because only a small fraction of cells in the heart is epicardial, we fluorescence-activated cell sorting (FACS)-purified cells from hearts extracted from *TgBAC(tcf21:H2B-Dendra2)*^*ox182*^, *TgBAC(tbx18:myr-eGFP)*^*ox184*^, and *TgBAC(wt1b:H2B-Dendra2)*^*ox186*^ transgenic embryos (Figure 2A; see Figures S2G–S2K for FACS gating plots). To enhance cell clustering in our dataset, we also isolated cells from non-epicardial cardiac tissues such as myocardium (*Tg(myl7:eGFP)*^*f1*^) (Huang et al., 2003), endocardium (*Tg(kdrl:GFP)*^*s843*^) (Beis et al., 2005), and blood (*Tg(gata1a:DsRed)*) (Traver et al., 2003), as well as cells from wild-type (WT) hearts. To enrich for *tcf21*^-^ epicardial cells, we purified non-fluorescent cells from *tcf21*:DsRed2; *myl7*:eGFP; *kdrl*:GFP; *gata1a*:DsRed quadruple transgenic reporter hearts. Following sequencing, we used the Pagoda pipeline (Fan et al., 2016) and dimensionality reduction to uncover three distinct epicardial cell populations (Figure 2B). These populations, termed Epi1, Epi2, and Epi3, were predominantly formed by cells isolated from the epicardial reporter hearts, and cells within these subpopulations expressed *tcf21*, *tbx18*, and *wt1b* in a differential manner (Figure 2C). *tbx18* was mostly expressed in cells within Epi1 and Epi2 and *tcf21* within Epi1 and Epi3, whereas *wt1b* was confined to subsets of cells within Epi1 and, to a lesser extent, Epi3. We further identified five distinct non-epicardial cell clusters expressing known markers of other cardiac cell types, namely cardiomyocytes (Figure 2C, CMs; *vmhc* [Yelon et al., 1999]), erythroid hematopoietic cells (Figure 2C, eHCs; *gata1a* [Lyons et al., 2002]), myeloid or leukocyte hematopoietic cells (Figure 2C, mHCs; *lcp1* [Kell et al., 2018]), neural cells (Figure 2C, NCs; *elavl3* [Park et al., 2000]) and mesenchymal cells (Figure 2C, MCs; *postna* [Snider et al., 2009]). This classification was supported by the enrichment of additional genes (Figure S3) and by the over-representation of gene ontology (GO) terms within the individual cell populations (Figure 2D). For example, cells in Epi1, Epi2, and Epi3 expressed many genes associated with epithelial cell fate commitment. Cells in Epi1 were enriched for genes associated with cell adhesion (Fisher’s exact test; p = 1.3E−07) and epithelial migration (p = 6.1E−07), fitting the epicardial capacity to migrate as an epithelial cell sheet (Wang et al., 2015). Cells in Epi2 expressed genes involved in vasoconstriction (p = 0.0227) and cell migration involved in heart development (p = 0.0021), suggesting they might fulfill a function outside the epicardial cell layer. Cells in Epi3 were enriched for genes associated with white blood cell migration (p = 0.0065) and axon extension (p = 0.0027), suggesting they might guide non-epicardial cells into the developing heart. To compare the reporter-based results described in Figure 1 with the endogenous transcriptomic data, we quantified the expression of *tcf21*, *tbx18*, and *wt1b* in Epi1, Epi2, and Epi3 and found that only Epi1 contained cells that co-expressed all three markers (Figure 2E). Most cells in Epi2 exclusively expressed *tbx18*, and many cells in Epi3 exclusively expressed *tcf21*. Thus, the 5 dpf zebrafish heart contains three transcriptionally distinct epicardial cell populations that differentially express *tcf21*, *tbx18*, and *wt1b*.

### Epi1, Epi2, and Epi3 Are Distinct in Their Transcriptomic Profile and Spatial Distribution within the Developing Heart

**Figure 3:**
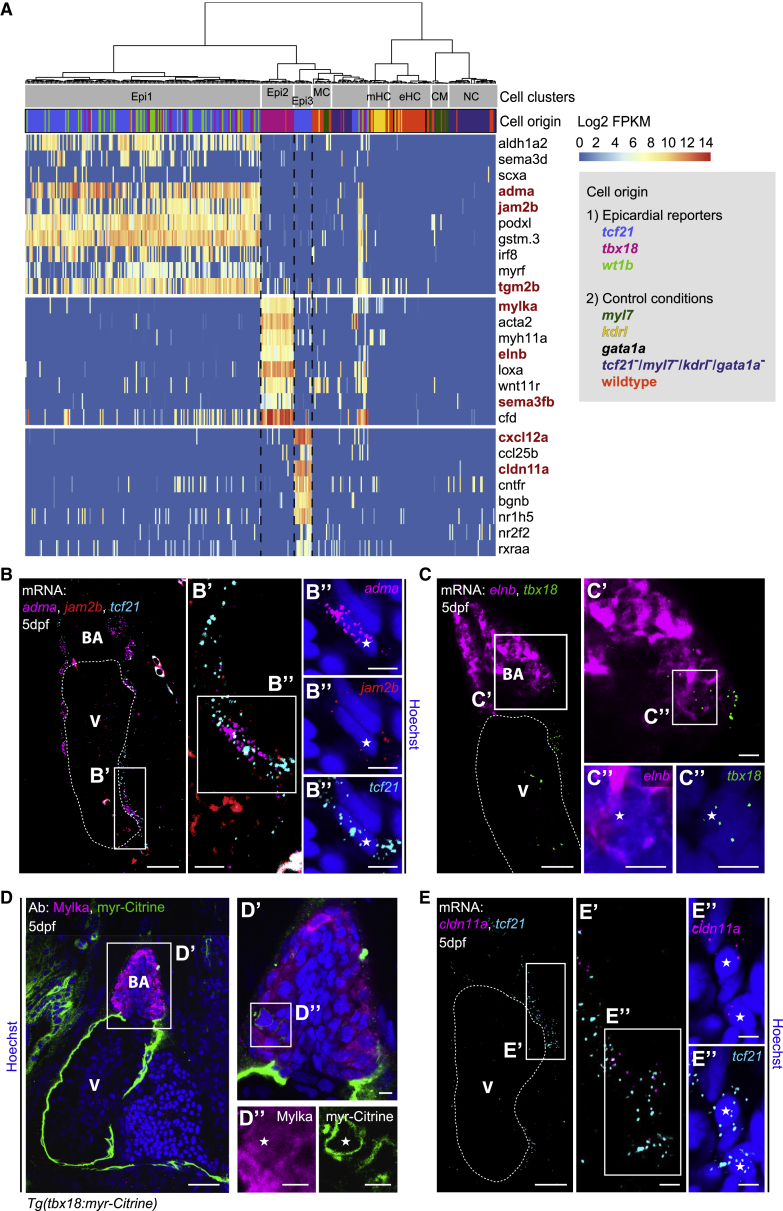
Transcriptionally Distinct Epicardial Subpopulations Epi1–3 Localize to Different Regions of the Developing Heart (A) Marker gene expression in Epi1–3. Cells were clustered in an unsupervised manner (columns). Color key indicates range of log_2_ transformed FPKM values. Genes analyzed further are highlighted in red. CM, cardiomyocyte; eHC, erythroid hematopoietic cell; mHC, myeloid hematopoietic cell; NC, neural cell; and MC, mesenchymal cell. (B) mRNA staining of the Epi1 markers *adma* (magenta), *jam2b* (red), and *tcf21* (cyan) in a 5 dpf heart. (B′ and B″) A nucleus (asterisk) in the epicardial region surrounded by *adma*, *jam2b*, and *tcf21*. (C) mRNA staining of the Epi2 marker *elnb* (magenta) and *tbx18* (green) at 5 dpf. (C′ and C″) Overlap of *elnb* and *tbx18* in the BA. (D) Antibody staining of Mylka and *tbx18*:myr-Citrine at 5 dpf. (D′ and D″) Overlap of Mylka and myr-Citrine in the BA. (E) mRNA staining of the Epi3 marker *cldn11a* (magenta) and *tcf21* (cyan) at 5 dpf. (E′ and E″) Two nuclei (asterisks) in the epicardial region between BA and atrium in close proximity to *cldn11a* and *tcf21*. Scale bars: 20 μm in (B)–(E) and 5 μm in (B′)–(E′) and (B″)–(E″). Color channels were adjusted separately for brightness and contrast. (B)–(E) are single optical sections. V, ventricle; BA, bulbus arteriosus.

Next, we interrogated the transcriptomic profiles of Epi1, Epi2, and Epi3. Single-cell gene enrichment analysis using scde (Kharchenko et al., 2014) showed that the retinoic acid synthesizing enzyme *retinaldehyde dehydrogenase* (*aldh1a2*), a known epicardial marker (Kikuchi et al., 2011, Lepilina et al., 2006), labeled Epi1 (Figure 3A). Epi1 cells also expressed genes without a previously ascribed epicardial function, such as the signaling peptide *adrenomedullin a* (*adma*), and the adhesion molecules *junctional adhesion molecule 2b* (*jam2b*) and *podocalyxin like* (*podxl*), suggesting that Epi1 might form the main epicardial cell sheet enveloping the heart. Using multiplexed hybridization chain reaction (HCR) *in situ* staining, we observed that both *adma* and *jam2b* mRNAs were present within the epicardial cell layer at 5 dpf, co-localizing with *tcf21* transcripts (Figures 3B–3B″).

In addition to *tbx18*, Epi2 cells expressed smooth muscle cell markers such as *smooth muscle actin* (*acta2*) and *myosin light chain kinase* (*mylka*), as well as extracellular matrix components such as *lysyl oxidase a* (*loxa*) and *elastin b* (*elnb*) (Figure 3A). *elnb* was previously shown to be specifically expressed in the BA of the zebrafish heart (Moriyama et al., 2016). Accordingly, we found the BA to be the only tissue in the 5 dpf zebrafish heart that expressed *elnb* mRNA (Figures 3C–3C″), as well as Mylka protein (Figures 3D–3D″). Furthermore, *tbx18* mRNA co-localized with *elnb* mRNA in the BA, and multiple Mylka^+^ cells were co-labeled by *tbx18*-driven myr-Citrine. These data reveal that Epi2 represents *tbx18*^*+*^ cells exclusively located in the smooth muscle layer of the BA.

RNA velocity analysis (La Manno et al., 2018) indicated that Epi2 cells might originate from cells resembling an Epi1 subpopulation (Figure S4A). Furthermore, pseudotime trajectories (Trapnell et al., 2014) separated Epi1 from Epi2, but also revealed a subset of cells in Epi1 positioned proximally to Epi2 (Figure S4B), suggesting that transcriptional transition from Epi1 to Epi2 could drive cellular transdifferentiation. To establish a lineage relationship between Epi2 and the epicardium beyond *tbx18* expression, we performed a tracing experiment using a *tcf21*-driven Cre *TgBAC(cryaa:EGFP,tcf21:Cre-ERT2)*^*pd42*^ (Kikuchi et al., 2011) crossed to the ubiquitous labeling line *Tg(ubi:Switch)* (Mosimann et al., 2011). We incubated embryos with 4-hydroxytamoxifen (4-OHT) from either 10 hpf or 43 hpf, shortly before the PEO starts to form (Peralta et al., 2013, Serluca, 2008). Similar to previous findings in the adult zebrafish BA (Kikuchi et al., 2011), we observed *tcf21-*derived cells in the smooth muscle layer of the larval BA, independently of when the 4-OHT treatment was started (Figures S4C–S4E, p = 0.1614). This finding strongly supports an epicardial origin of Epi2 cells rather than an upregulation of *tbx18* mRNA in the BA. However, lineage tracing with a newly generated *TgBAC(wt1b:Cre-2A-mCherry)*^*ox142*^ line did not identify *wt1b*-derived cells in the BA (Figures S4F and S4G), indicating that cells in Epi2 never express *wt1b*. Endogenous *wt1b* co-localized with *mCherry* transcripts (Figure S4H), which, together with co-localization with the *TgBAC(wt1b:H2B-Dendra2)*^*ox186*^ reporter expression (Figure S4I), validated the activity of the *TgBAC(wt1b:Cre-2A-mCherry)*^*ox142*^ line.

Cells in Epi3 expressed *claudin 11a* (*cldn11a*), coding for an essential tight junction component (Gow et al., 1999) (Figure 3A). We observed co-localization of *cldn11a* and *tcf21* transcripts by HCR (Figures 3E–3E″), mostly in the epicardial region between the BA and the atrioventricular boundary. The spatial restriction of *cldn11a* expression was consistent with the small number of cells forming Epi3 (Figure 2C). Additionally, cells in Epi3 expressed the retinoic acid-responsive factors *nr1h5* and *rxraa*, known to interact in mammalian cells (Otte et al., 2003), and the chemokine *cxcl12a* (Figure 3A). Therefore, Epi3 might be responsive to retinoic acid and involved in the guidance of non-epicardial cells.

### The Epi1-Enriched Gene *Transglutaminase 2b* Is Essential for Maintaining the Integrity of the Epicardial Cell Layer

To address whether epicardial heterogeneity underlies distinct cell fates and/or function, we used CRISPR-Cas9-mediated gene knockout. As single guide RNAs (sgRNAs) are rapidly degraded when not incorporated into Cas9 protein (Burger et al., 2016, Hendel et al., 2015), we used the Activator (Ac)-Dissociation (Ds) transposable element system (Emelyanov et al., 2006, McCLINTOCK, 1950) to stably express sgRNAs (Chong-Morrison et al., 2018) in zebrafish. We first cloned sgRNAs targeting *Citrine* and *eGFP* into the Ac-Ds U6a mini-vector and injected reporter embryos with the corresponding sgRNA vector, Ac and Cas9 mRNAs. This led to widespread disruption of fluorescence (Figure S5), indicating the system robustly and efficiently perturbed *Citrine* and e*GFP* expression using somatic CRISPR-Cas9 genome editing.

**Figure 4:**
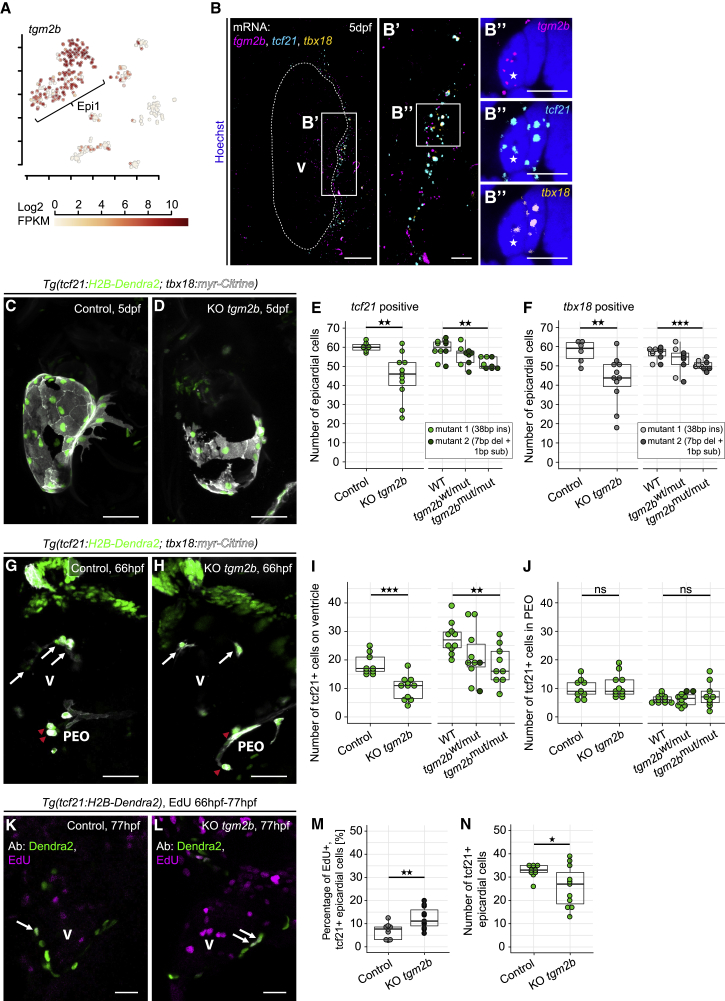
*Transglutaminase 2b* Is Enriched in Epi1 and Functions to Maintain the Integrity of the Epicardial Cell Layer (A) Expression of *tgm2b*. Color key indicates range of log_2_ transformed FPKM values. (B) mRNA staining of *tgm2b* (magenta), *tcf21* (cyan), and *tbx18* (orange) in a 5 dpf heart. (B′ and B″) A nucleus (asterisk) in the epicardial region surrounded by *tgm2b*, *tcf21*, and *tbx18*. (C) The epicardium in a 5 dpf control larva. (D) Disrupted epicardial integrity in a 5 dpf KO *tgm2b* larva. (E) Absolute quantification of *tcf21*:H2B-Dendra2^+^ epicardial cell numbers at 5 dpf in transient *tgm2b* knockouts (Control, KO *tgm2b*) and stable *tgm2b* mutants (heterozygous, *tgm2b*^wt/mut^; homozygous, *tgm2b*^mut/mut^). Larvae from two mutant lines were analyzed. (F) Absolute quantification of *tbx18*:myr-Citrine^+^ epicardial cell numbers at 5 dpf. (G) The epicardium in a 66 hpf control embryo. White arrows indicate epicardial cells, and red arrowheads indicate cells in the PEO. (H) Reduced epicardial cell numbers in a 66 hpf KO *tgm2b* embryo. (I and J) Absolute quantification of *tcf21*:H2B-Dendra2^+^ epicardial (I) and proepicardial (J) cell numbers at 66 hpf. (K) Proliferation in the epicardium of a 77 hpf control larva (white arrows). (L) Increased epicardial proliferation in a KO *tgm2b* larva. (M) Relative quantification of proliferating epicardial cells. (N) Absolute quantification of *tcf21*:H2B-Dendra2^+^ epicardial cells. Scale bars: 50 μm in (C), (D), (G), and (H); 20 μm in (B); 10 μm in (K) and (L); and 5 μm in (B′) and (B″). Color channels were adjusted separately for brightness and contrast. (C), (D), (G), and (H) are projections; (B), (K), and (L) are single optical sections. Data are represented as median, first, and third quartiles (box). Significance was calculated using Welch’s t test. ^∗^p < 0.05, ^∗∗^p < 0.01, ^∗∗∗^p < 0.001. V, ventricle; Ab, antibody. See also Figure S6.

*Transglutaminase 2b* (*tgm2b*), a gene enriched in Epi1 (Figure 4A), codes for a protein-crosslinking enzyme previously studied during bone formation (Deasey et al., 2012). HCR analysis showed that *tgm2b* transcripts were broadly distributed within the epicardium and co-localized with *tcf21* and *tbx18* mRNAs (Figures 4B–4B″). We designed *tgm2b* sgRNAs targeting the region coding for the active site of glutamyltransferase activity (Figures S6A and S6B). Somatic knockout of *tgm2b* led to defects in the epicardial cell sheet at 5 dpf (Figures 4C and 4D). In severe cases, large regions of the epicardial cell layer were missing, and epicardial cell numbers were significantly reduced in *tgm2b* knockout larvae (Figure 4E, p = 0.0016 and 4F, p = 0.005). We further established two stable *tgm2b* mutant lines, *tgm2b*^*38ins/38ins*^ (mutant line 1, ox188, carrying 38-bp insertion in the *tgm2b* coding region) and *tgm2b*^*7del1sub/7del1sub*^ (mutant line 2, ox189, carrying 7-bp deletion and 1-bp substitution) (Figure S6C). In both mutants, the resulting frameshift created an early translational termination site between WT residues 289 and 290 (length of WT tgm2b protein is 679 aa). *tgm2b* mutant larvae showed a reduction in epicardial cell numbers at 5 dpf, comparable with the phenotype observed in the transient *tgm2b* knockout (Figure 4E, p_*wt*_ _versus homozyg_ = 0.0011 and 4F, p_wt_ _versus homozyg_ = 0.0003). This phenotype might be due to a reduction in the number of epicardial cells that migrate from the PEO and attach to the myocardium, because the number of mutant *tcf21*^*+*^ epicardial cells was already lowered at 66 hpf (Figures 4G–4I, p_somatic_ _KO_ = 0.0002, p_wt_ _versus homozyg_ = 0.0051), yet the numbers in the PEO of control and mutant embryos were similar (Figure 4J, p_somatic_ _KO_ = 0.5951, p_wt_ _versus homozyg_ = 0.3964). We tested whether *tgm2b* might inhibit epicardial cell proliferation (Figures 4K–4N). Control and *tgm2b* transient knockout embryos were incubated with EdU from 66 hpf to 77 hpf and, surprisingly, we observed an increased number of proliferating *tcf21*^+^ cells in *tgm2b* knockout hearts. This suggests a compensatory mechanism for the decrease in epicardial cell numbers (Figures 4K–4M, p = 0.0062 and 4N, p = 0.0352).

In conclusion, *tgm2b* plays a critical role in maintaining the integrity of the forming epicardium and implicates Epi1 in ensuring the formation of a cohesive epithelial sheet of epicardial cells during heart development.

### The Epi2-Enriched Gene *sema3fb* Regulates the Number of *tbx18*^+^ Cells that Contribute to the Smooth Muscle Layer of the Outflow Tract

**Figure 5:**
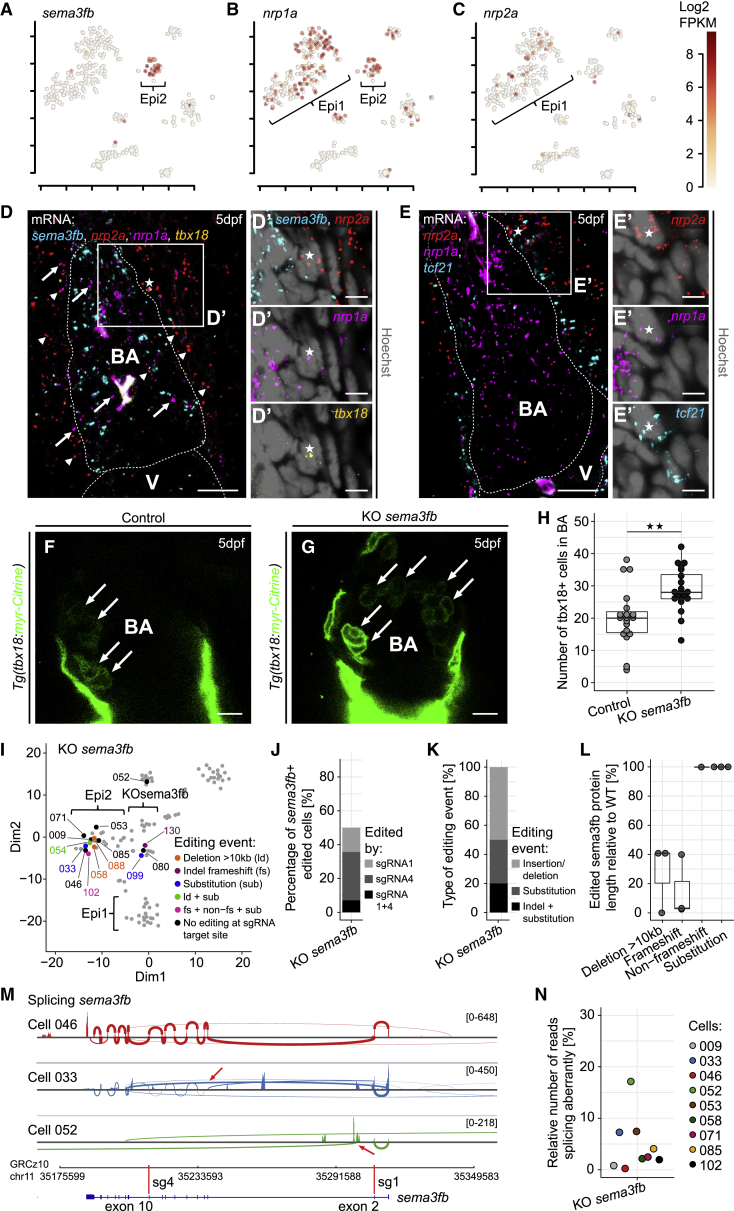
*Semaphorin 3fb* Is a Marker of Epi2 and Controls the Number of *tbx18*^+^ Cells in the Bulbus Arteriosus (A–C) Expression of *sema3fb* (A), *nrp1a* (B), and *nrp2a* (C). Color key indicates range of log_2_ transformed FPKM values. (D) mRNA staining of *sema3fb* (cyan), *nrp2a* (red, arrowheads), *nrp1a* (magenta, arrows), and *tbx18* (orange) at 5 dpf. (D′) Close proximity of *nrp2a*, *nrp1a,* and *tbx18* to a nucleus (asterisk) at the BA boundary. (E) mRNA staining of *nrp2a* (red), *nrp1a* (magenta), and *tcf21* (cyan) at 5 dpf. (E′) A nucleus (asterisk) in close proximity to *nrp2a*, *nrp1a*, and *tcf21*. (F) The BA in a 5 dpf control larva. (G) Increased numbers of *tbx18*:myr-Citrine^+^ cells (arrows) in the BA of a KO *sema3fb* larva. (H) Absolute quantification of *tbx18*:myr-Citrine^+^ cell numbers in the BA at 5 dpf. (I–N) Analysis of gene editing in single *sema3fb*-mutated epicardial cells. (I) t-SNE plot showing cell clusters and editing events. Numbers indicate cell identities, and colors depict editing events. Ld, large deletion; fs, frameshift; and sub, substitution. (J) Relative number of Cas9-edited *sema3fb*^+^ cells. Colors indicate the *sema3fb* sgRNA target site edited. (K) Frequency of editing event types. (L) Inferred edited sema3fb protein lengths, relative to WT. (M) Sashimi plots showing splicing of *sema3fb*. Arrows highlight reads splicing abnormally. Red bars indicate sgRNA target sites. (N) Relative number of *sema3fb* splicing reads that spliced abnormally. Scale bars: 10 μm in (D)–(G) and 5 μm in (D′) and (E′). Color channels were adjusted separately for brightness and contrast. (D)–(G) are single optical sections. Data in (H) and (L) are represented as median, first, and third quartiles (box). Significance calculated using Welch’s t test. ^∗∗^p < 0.01. V, ventricle; BA, bulbus arteriosus. See also Figure S6.

One of the most enriched factors in Epi2 was *sema3fb*, coding for a chemo-repellant known to be involved in neural patterning (Terriente et al., 2012) (Figure 5A). sema3fb belongs to the secreted class 3 semaphorins, which require a receptor complex consisting of a neuropilin (nrp1a/b or nrp2a/b) and a plexin for activity (reviewed in Neufeld et al. [2016]). We observed that *nrp1a* was co-expressed with *sema3fb* in Epi2, whereas most of the *nrp2a*^+^ cells were assigned to Epi1 that also featured *nrp1a* expression (Figures 5B and 5C). HCR analysis revealed that *sema3fb* was strongly expressed within the BA and only sparsely in the surrounding areas (Figures 5D and 5D′). In contrast, *nrp2a* mRNA was almost exclusively located in areas surrounding the BA (Figure 5D, arrowheads). *nrp1a* mRNA was present in close proximity to *sema3fb* in the BA (Figure 5D, arrows) and also adjacent to the BA. At the BA boundary, both *nrp1a* and *nrp2a* were detected in close proximity to *tbx18* (Figure 5D′, asterisk) and *tcf21* (Figure 5E, asterisk in 5E′). In summary, these results suggest that cells within the BA express *sema3fb* and *nrp1a* (and not *nrp2a*), whereas cells outside the BA, such as those within the adjacent epicardial layer, express *nrp2a* and *nrp1a* (and not *sema3fb*).

The expression pattern of *sema3fb* and the *nrp* receptors, together with the association of Epi2 to cardiac cell migration (Fisher’s exact test; p = 0.00211) (Figure 2D), suggested that Epi2 could be a source of repulsive guidance cues for other epicardial cells migrating into the BA. To test this hypothesis, we performed somatic mutagenesis of *sema3fb* (Figures S6D and S6E), which led to a significant increase in the number of *tbx18*^+^ cells in the BA (Figures 5F–5H, p = 0.0021), supporting a role for *sema3fb* in restricting the number of Epi2 cells in the outflow tract. We investigated whether this function might involve inhibition of *tbx18*^*+*^ cell proliferation in the BA (Figures S6F and S6G, arrows). However, there was no significant difference in the number of proliferating *tbx18*^+^ cells, although many more *tbx18*:myr-Citrine^+^ cells were detected within the BA in *sema3fb* knockouts (Figures S6F–S6H, p = 0.8202). This result argues against a role for *sema3fb* in restricting cell proliferation and suggests that it controls the number of *tbx18*^*+*^ cells in the BA, by restricting their migration from surrounding tissues such as the epicardium.

We further isolated cells from *tbx18*:myr-Citrine^+^*sema3fb* transient knockout hearts and performed scRNA-seq, adapting the recently established TARGET-seq method (Rodriguez-Meira et al., 2019). TARGET-seq allows the detection of mutations in both genomic DNA and transcriptome-derived cDNA at the single-cell level. We identified multiple types of *sema3fb*-gene-editing events in cells with sequencing read coverage of the *sema3fb* sgRNA target sites (Figures 5I–5K). Many of these (Figure 5I) were located in a cell cluster that was enriched in Epi2 markers (Figures 3A and S6I). However, other cells analyzed for *sema3fb* gene editing formed part of an additional cluster termed “KOsema3fb” only present in the *sema3fb* knockout dataset (Figure 5I). The KOsema3fb cluster did not feature the widespread presence of Epi1 or Epi2 markers, although multiple cells in this cluster expressed *tbx18* and *Citrine* mRNA (Figure S6I).

We found that 50% of the analyzed cells carried editing events, with both sgRNAs contributing to these (Figure 5J). Editing event types comprised insertions-deletions (indels) and nucleotide substitutions (Figure 5K). We analyzed *sema3fb* open reading frames (ORFs) after gene editing and found large deletions (Kosicki et al., 2018) and indel-induced frameshifts (Figure 5L), which reduced sema3fb protein length in over a third of the samples analyzed (Figure 5I). In addition, in up to 20% of the sequenced transcripts, we found aberrant splicing of *sema3fb* cDNA (Figures 5M and 5N), such as splicing across multiple *sema3fb* exons (Figure 5M, cell 033, arrow) or abnormal splicing from a possible cryptic intronic site (Figure 5M, cell 052, arrow) (Figure 5N). Aberrant splicing of *sema3fb* was absent in the non-knockout scRNA-seq dataset (Figure S6J), indicating that it was not an ordinarily occurring form of alternative splicing.

These results illustrate the impact of somatic *sema3fb* knockout on Epi2 cells and indicate that it reduces sema3fb functionality in around half of all *sema3fb*^+^ cells.

### Epi3-Enriched *cxcl12a* Attracts Leukocytes to the Developing Heart

**Figure 6:**
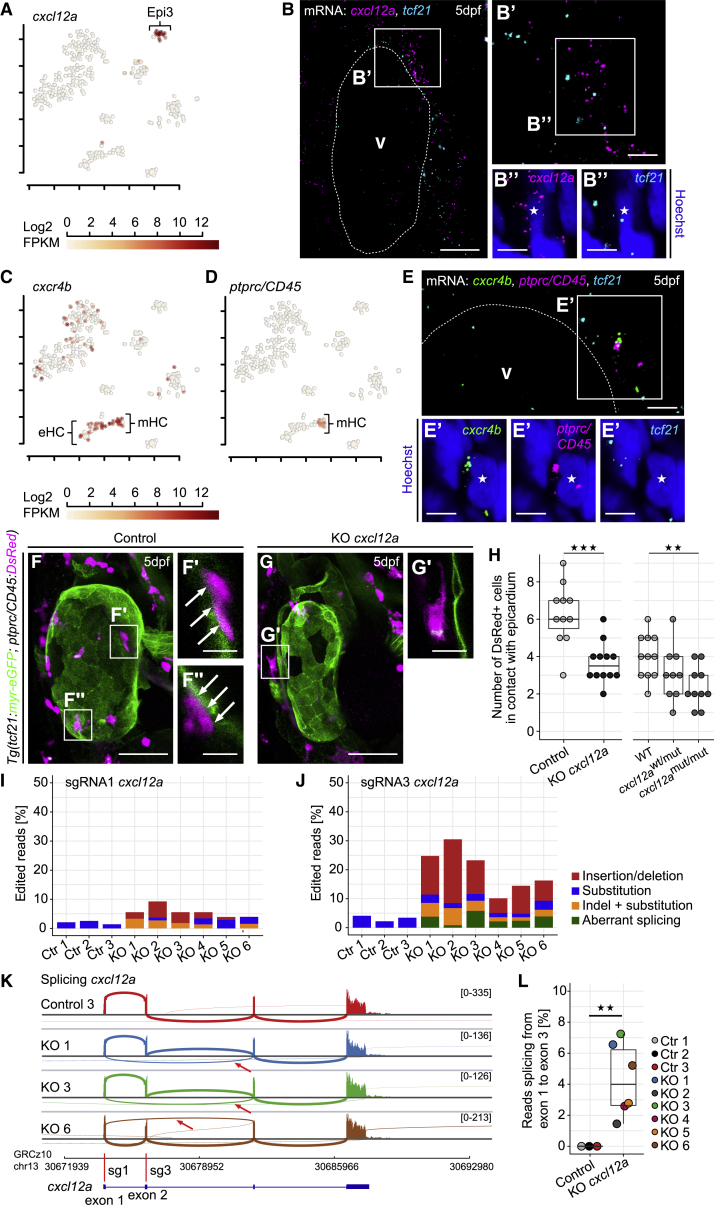
The Chemokine *cxcl12a* Is Expressed in Epi3 and Attracts *ptprc*^+^ Leukocytes to the Epicardium (A) Expression of *cxcl12a*. Color key indicates range of log_2_ transformed FPKM values. (B) mRNA staining of *cxcl12a* (magenta) and *tcf21* (cyan) at 5 dpf. (B′ and B″) A nucleus (asterisk) in the epicardial region between BA and atrium surrounded by *cxcl12a* and *tcf21*. (C and D) Expression of *cxcr4b* (C) and *ptprc* (D). Color key indicates range of log_2_ transformed FPKM values. (E) mRNA staining of *cxcr4b* (green), *ptprc* (magenta), and *tcf21* (cyan) at 5 dpf. (E′) A nucleus (asterisk) in close proximity to *cxcr4b* and *ptprc*. (F) The heart in a 5 dpf control larva. (F′ and F″) *ptprc/Cd45*:DsRed^+^ cells in contact with the epicardium (arrows). (G and G′) Fewer *ptprc/CD45*:DsRed^+^ cells on the heart of a 5 dpf KO *cxcl12a* larva. (H) Absolute quantification of *ptprc/CD45*:DsRed^+^ cells in contact with the epicardium in transient *cxcl12a* knockouts (Control, KO *cxcl12a*) and stable *cxcl12a* mutants (heterozygous, *cxcl12a*^wt/mut^; homozygous, *cxcl12a*^mut/mut^). (I and J) RNA-seq analysis of *cxcl12a* gene editing in single control (ctr) and *cxcl12a*-knockout (KO) larvae. Relative numbers of edited reads that spanned the target site of *cxcl12a* sgRNA1 (I) or sgRNA3 (J). (K) Sashimi plots showing splicing of *cxcl12a*. Arrows highlight reads splicing from exon 1 into exon 3. Red bars indicate sgRNA target sites. (L) Relative number of reads splicing from *cxcl12a* exon 1 that spliced from exon 1 into exon 3. Scale bars: 50 μm in (F) and (G); 20 μm in (B); 10 μm in (F′), (F″), and (G′), and 5 μm in (B′), (B″), (E), and (E′). Color channels were adjusted separately for brightness and contrast. (F) and (G) are projections; (B), (E), (F′), (F″), and (G′) are single optical sections. Data in (H) and (L) are represented as median, first, and third quartiles (box). Significance calculated using Welch’s t test. ^∗∗^p < 0.01, ^∗∗∗^p < 0.001. V, ventricle; eHC, erythroid hematopoietic cell; mHC, myeloid hematopoietic cell. See also Figure S7.

Epi3 cells expressed the chemokine *cxcl12a* (Figure 6A). HCR revealed that the spatial distribution of *cxcl12a* transcripts was restricted to an area between BA and atrium (Figures 6B–6B″), very similar to *cldn11a* (Figure 3E), and a subset of *cxcl12a* was located in close proximity to *tcf21*, indicating co-expression.

GO term analysis showed that Epi3 was enriched for genes involved in leukocyte chemotaxis (Fisher’s exact test; p = 0.0067), a process also associated with the myeloid hematopoietic cell cluster (Fisher’s exact test; p = 7.2E–06) (Figure 2D). Interestingly, the *cxcl12a* cognate receptor *cxcr4b* was strongly present in this population (Figure 6C, mHC), which was also enriched in *ptprc* (*CD45*) (Figure 6D), a pan-leukocyte marker in zebrafish (Bertrand et al., 2008). HCR revealed areas on the ventricular surface in which *cxcr4b* and *ptprc/CD45* were co-localized (Figures 6E and 6E′, asterisk). *tcf21* was present in neighboring regions but was not closely co-localizing with *cxcr4b* or *ptprc/CD45*. This suggested that epicardial cxcl12a might be attracting *ptprc/CD45*^+^ leukocytes to the developing heart. We thus designed sgRNAs targeting the *cxcl12a* gene (Figures S7A and S7B). Somatic loss of *cxcl12a* decreased the number of *ptprc/CD45*:DsRed^+^ cells (Bertrand et al., 2008) that were present on the surface of the heart (Figures 6F–6H, p = 0.0003). We observed direct contact between *ptprc/CD45*:DsRed^+^ cells and the outer epicardial surface in controls (arrows in Figures 6F′ and 6F″) and reduced contacts in *cxcl12a* knockout larvae (Figure 6G′). We next established a stable mutant line featuring a 15-bp deletion at the start of the *cxcl12a* coding sequence, *cxcl12a*^*15del/15del*^ (ox190) (Figure S7C). Homozygous mutant *cxcl12a*^*15del/15del*^ embryos showed a defect in lateral line development, recapitulating a previously published *cxcl12a* mutant phenotype arising from a different mutation (Valentin et al., 2007) (Figures S7D–S7F). Importantly, the number of *ptprc/CD45*:DsRed^+^ cells in contact with the epicardium was significantly reduced in *cxcl12a* mutant larvae (Figure 6H, p_*wt*_ _versus homozyg_ = 0.0042), consistent with our somatic *cxcl12a* knockout experiments. These data identify a role for *cxcl12a* in chemo-attracting leukocytes to the surface of the developing heart.

RNA-seq analysis of *cxcl12a* mRNA in single transient knockout larvae detected a variety of gene editing events (Figures 6I and 6J), with indels only detected in *cxcl12a* knockouts. Around 5% of the reads at the target site of *cxcl12a* sgRNA1 were edited (Figure 6I), whereas the editing frequency at the *cxcl12a* sgRNA3 target site was around 30% (Figure 6J). Furthermore, a significant number of reads spliced aberrantly from *cxcl12a* exon 1 into exon 3 in the *cxcl12a* transient knockouts (Figures 6J and 6K, arrows, and 6L). Additionally, we found that most indels should result in shortened *cxcl12a* ORFs (Figures S7G and S7H), with aberrant splicing reducing the *cxcl12a* ORF to only 25% of its non-edited length (Figure S7H). Despite the identified mutations, no significant reduction in *cxcl12a* mRNA levels was detected (Figure S7I, p = 0.2221).

In summary, we found clear evidence of somatic *cxcl12a* gene editing. All *cxcl12a*-mutated larvae featured indels at both sgRNA target sites, as well as aberrant *cxcl12a* splicing, which likely affected cxcl12a protein size and function.

## Discussion

Understanding epicardial cell fate and lineage potential to date has been constrained by the limited set of so-called canonical markers which are either, not entirely specific for epicardial cells or, fail to label the entire epicardial cell pool. Here, we identify epicardial cell subpopulations within the developing zebrafish heart, characterize their transcriptional profiles at the single-cell level, and functionally test their involvement during heart development in zebrafish.

**Figure 7:**
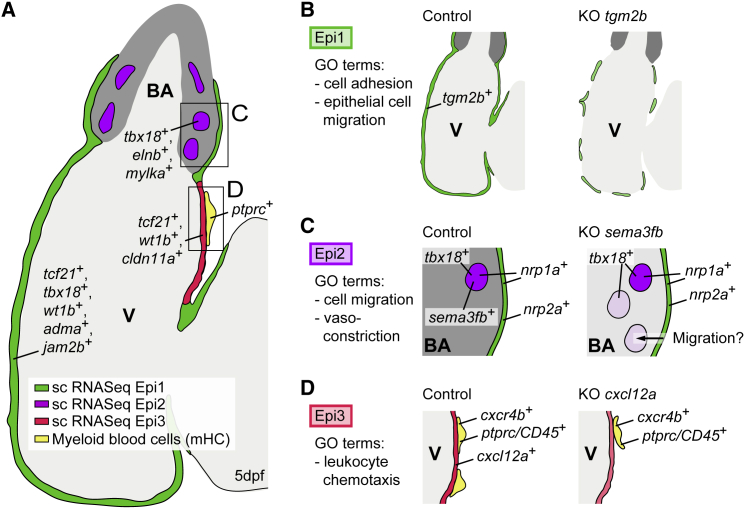
Summary of the Distinct Epicardial Cell Populations in the Developing Zebrafish Heart (A) Zebrafish heart at 5 dpf, showing the distributions of Epi1-3. (B) Loss of *tgm2b* disrupts the integrity of the epicardial cell layer. (C) Loss of *sema3fb* increases the number of Epi2 cells in the BA, possibly owing to increased cell migration from the epicardium. (D) Loss of *cxcl12a* decreases the number of myeloid cells on the heart. V, ventricle; BA, bulbus arteriosus.

Our study provides insights into the heterogeneity and associated functional diversity of the developing epicardium. We identify the epicardial cell populations Epi1, Epi2, and Epi3, their distinct genetic programs and spatial distributions (Figure 7A). GO analysis of the most highly enriched genes in each of these clusters and perturbation of signature genes provided insights into the maintenance of epicardial integrity, formation of the outflow tract, and myeloid cell recruitment into the developing heart. Because of the relatively low cell numbers analyzed in our dataset, there might, however, be additional scarce epicardial subpopulations that remained undetected.

### Maintenance of Epithelial Integrity within the Forming Epicardium

GO term analysis of Epi1 genes indicated a possible Epi1 role in the formation of a coherent epicardial cell sheet that migrates to envelope the myocardium. We focused on *tgm2b*, whose functional perturbation decreased epicardial cell numbers (Figure 7B). Interestingly, cell numbers in the PEO were unaffected by *tgm2b* knockout (Figure 4J), arguing against an early specification defect. Similarly, defective proliferation in the early epicardium was excluded as a possible cause for the reduction in epicardial cell numbers (Figures 4K–4M). Instead, there might be a reduction in the number of pro-epicardial cells that are able to attach to the myocardium during epicardium formation (Kwee et al., 1995, Sengbusch et al., 2002, Yang et al., 1995), a process also known to require heartbeat-derived fluid flow (Peralta et al., 2013). Additionally, *tgm2b* might be involved in the regulation of cell-cell contacts within the epicardial cell layer.

### The Regulation of Epicardial Cell Migration into the Outflow Tract

GO term analysis of Epi2-enriched genes suggested that this subpopulation might be linked to the smooth muscle wall of the BA. Indeed, many Epi2-specific genes were exclusively expressed in the developing smooth muscle layer of the BA. We show that these *tbx18*^+^ Epi2 cells had a *tcf21* origin but never expressed *wt1b*. Pseudotime analysis suggested that a subset of Epi1 cells might be in a transition state toward Epi2 (Figures S4A and S4B), supported by our *tcf21* lineage tracing results. The BA of the adult zebrafish heart is a positive regulator of epicardial cell migration (Wang et al., 2015). Following cardiac injury, the BA is thought to drive the coordinated movement of the regenerating epicardial cell sheet via Sonic hedgehog signaling (Wang et al., 2015). The secreted diffusible chemorepulsive factor sema3fb is enriched in Epi2 and functions to negatively regulate the number of epicardial cells that populate the BA (Figure 7C), therefore, acting as a gatekeeper controlling the number of cells in the outflow tract. Both sema3 signaling receptors, nrp1a and nrp2a, were expressed in the epicardium. NRP1 and NRP2 have been reported to interact with SEMA3 ligands differentially, with SEMA3F specifically binding to NRP2 and SEMA3A partnering with NRP1 (reviewed in Neufeld et al. [2016]). The fact that *nrp2a* transcripts were abundant in the vicinity of the BA, but excluded from the inside, supports a model in which nrp2a^+^ Epi1 cells are retained outside of the BA by the chemorepulsive activity of sema3fb, whereas nrp2a^−^ epicardial cells are able to migrate into and home to the BA. In transient *sema3fb* knockouts, we identified a *sema3fb*^+^, partially *tbx18*^+^ cell cluster not present in the WT setting (Figures 3A and S6I), which may represent a population of cells that have abnormally homed to the BA. Our work highlights the importance of understanding the mechanisms enabling the migration of epicardial cells in response to diffusible guidance cues.

### Epicardial Cell Regulation of Leukocyte Homing into the Heart

Epi3 might be involved in the guidance of white blood cells into the developing heart. Links between the epicardium and the immune system have been implicated during mouse fetal heart development, where CD68^+^ macrophages required the intact epicardium for their recruitment into the developing heart, a process dependent on the expression of *Wt1* (Stevens et al., 2016). Our study similarly reveals that the Epi3-enriched chemokine *cxcl12a* is necessary for the recruitment and/or retention of *ptprc/CD45*^+^ hematopoietic cells onto the surface of the heart (Figure 7D). These leukocyte or myeloid cells express the cognate chemokine receptor *cxcr4b*. Although there appear to be only a few Epi3 cells in the epicardium, their strong expression of the diffusible *cxcl12a* in the confined space of the pericardial cavity is likely to affect cxcl12a responsive cells in the vicinity, such as the *cxcr4b*^+^/*CD45*^+^ cells we analyzed. Thus, our work unravels a developmental mechanism by which an epicardial subpopulation regulates leukocyte recruitment through the expression of a paracrine signaling molecule. Non-PEO-derived CD45^+^ hematopoietic cells were shown to contribute to the developing epicardium of the mouse heart (Balmer et al., 2014). Therefore, it will be of interest to further explore the possible role of *cxcr4b*^+^/*CD45*^+^ cells present on the developing zebrafish heart. *Cxcl12a* is also expressed in adult epicardial cells upon cardiac injury (Itou et al., 2012), and, in the mouse, epicardial-associated CD45^+^ hematopoietic cell clusters respond dynamically to myocardial infarction (Balmer et al., 2014). These cells co-express CD45^+^/*CXCR4*^+^/*CD11b*^+^ in the bone marrow and within the ischemic heart (Ghadge et al., 2017), suggesting that *cxcl12a* may recapitulate its developmental role following adult heart injury.

In conclusion, our scRNA-seq work has opened up avenues for understanding epicardial cell biology and provides insights into the formation of a critical lineage, which is essential during both heart development and adult heart regeneration. The identification of functional subpopulations of epicardial cells and their roles as sources of chemo-attractant and repellent signals for cellular cross-talk during development may be exploited to facilitate cell-based therapies to regenerate the injured heart.

## STAR★Methods

### Key Resources Table

**Table:** 

REAGENT or RESOURCE	SOURCE	IDENTIFIER
**Antibodies**		

chicken polyclonal anti-GFP	abcam	Cat# ab13970, RRID: AB_300798
Living Colors® DsRed Polyclonal Antibody rabbit	Clontech	Cat# 632496, RRID: AB_10013483
mouse monoclonal anti-Dendra2	Origene	Cat# TA180094, clone OTI1G6, RRID: AB_2622288
mouse monoclonal anti-Mlck	Sigma-Aldrich	Cat# M7905, RRID: AB_477243
rabbit polyclonal anti-cxcr4b	Genetex	Cat# GTX132244; RRID: AB_2827420

**Chemicals, Peptides, and Recombinant Proteins**		

RNase Inhibitor	Clontech	Cat# 2313A
SMARTScribe reverse transcriptase	Clontech	Cat# 639537
SeqAMP DNA polymerase	Clontech	Cat# 638509
Protease	Qiagen	Cat# 19155
Herculase II Fusion DNA Polymerase	Agilent	Cat# 600675

**Critical Commercial Assays**		

Quant-iT PicoGreen dsDNA Assay	Thermo Fisher Scientific	Cat# P11496
Nextera XT library preparation kit	Illumina	Cat# FC-131-1024
RNAqueous Micro Total RNA isolation kit	Ambion	Cat# AM1931
NextSeq ® 500/550 Mid Output Kit v2.5 (150 cycles)	Illumina	Cat# 20024904
NextSeq ® 500/550 High Output Kit v2.5 (75 cycles)	Illumina	Cat# 20024906
See STAR Methods section for Hybridization Chain Reaction (HCR) v3.0	Molecular Instruments	N/A
Click-iT™ EdU Alexa Fluor™ 647 Imaging Kit	Thermo Fisher Scientific	Cat# C10340
InFusion HD Cloning kit	Clontech	Cat# 638910

**Deposited Data**		

Raw and analysed single-cell RNA-seq data	This paper	GEO: GSE121750

**Experimental Models: Organisms/Strains**		

*TgBAC(tcf21:myr-tdTomato)*^*ox181*^	Riley and Sauka-Spengler laboratories	ox181
*TgBAC(tcf21:H2B-Dendra2)*^*ox182*^	Riley and Sauka-Spengler laboratories	ox182
*TgBAC(tcf21:myr-eGFP)*^*ox183*^	Riley and Sauka-Spengler laboratories	ox183
*TgBAC(tbx18:myr-eGFP)*^*ox184*^	Riley and Sauka-Spengler laboratories	ox184
*TgBAC(tbx18:myr-Citrine)*^*ox185*^	Riley and Sauka-Spengler laboratories	ox185
*TgBAC(wt1b:H2B-Dendra2)*^*ox186*^	Riley and Sauka-Spengler laboratories	ox186
*TgBAC(tcf21:myr-tdTomato;tbx18:myr-eGFP;wt1b:H2B-Dendra2)*^*ox187*^	Riley and Sauka-Spengler laboratories	ox187
*TgBAC(wt1b:Cre-2A-mCherry)*^*ox142*^	Riley and Sauka-Spengler laboratories	ox142
*Tg(tgm2b*^*38ins*^*;tcf21:H2B-Dendra2;tbx18:myr-Citrine)*^*ox188*^	Riley and Sauka-Spengler laboratories	ox188
*Tg(tgm2b*^*7del1sub*^*;tcf21:H2B-Dendra2;tbx18:myr-Citrine)*^*ox189*^	Riley and Sauka-Spengler laboratories	ox189
*Tg(cxcl12a*^*15del*^*; ptprc/CD45:DsRed; tcf21:myr-eGFP)*^*ox190*^	Riley and Sauka-Spengler laboratories	ox190
*TgBAC(tcf21:DsRed2)*^*pd37*^	Kikuchi et al., 2011	ZFIN: ZDB-TGCONSTRCT-110818-7
*TgBAC(tbx18:DsRed2)*^*pd22*^	Kikuchi et al., 2011	ZFIN: ZDB-TGCONSTRCT-110818-6
*TgBAC(cryaa:EGFP,tcf21:Cre-ERT2)*^*pd42*^	Kikuchi et al., 2011	ZFIN: ZDB-TGCONSTRCT-110818-8
*Tg(wt1b:eGFP)*^*li1*^	Perner et al., 2007	ZFIN: ZDB-TGCONSTRCT-071127-1
*Tg(myl7:eGFP)*^*f1*^	Huang et al., 2003	ZFIN: ZDB-TGCONSTRCT-070117-164
*Tg(kdrl:GFP)*^*s843*^	Beis et al., 2005	ZFIN: ZDB-TGCONSTRCT-070529-1
*Tg(gata1a:DsRed)*	Traver et al., 2003	ZFIN: ZDB-TGCONSTRCT-070117-38
*Tg(ptprc/CD45:DsRed)*^*sd3*^	Bertrand et al., 2008	ZFIN: ZDB-TGCONSTRCT-081120-3
*Tg(–3.5ubb:loxP-EGFP-loxP-mCherry)*	Mosimann et al., 2011	ZFIN: ZDB-TGCONSTRCT-110124-1
*Tg(ubb:loxPAmCyanSTOPloxPZsYellow)*	Zhou et al., 2011	ZFIN: ZDB-TGCONSTRCT-111115-6

**Oligonucleotides**		

Smart-seq2 LNA-TSO: AAGCAGTGGTATCAACGCAGAGTACATrGrG+G	Picelli et al., 2013	N/A
Smart-seq2 OligodT-ISPCR primer: aagcagtggtatcaacgcagagtacttttttttttttttttttttttttttttttvn	Picelli et al., 2013	N/A
Smart-seq2 ISPCR primer: AAGCAGTGGTATCAACGCAGAGT	Picelli et al., 2013	N/A
See Table S1 for BAC recombineering primers	This paper	N/A
See Table S2 for sgRNA cloning primers	This paper	N/A
See Table S3 for sgRNA/Cas9 nuclease activity test primers	This paper	N/A
See Table S4 for *sema3fb* and *cxcl12a* cDNA/ genomic DNA primers	This paper	N/A

**Recombinant DNA**		

pGEM-BirA-2A-H2B-Dendra2-SV40pA-FKF	This paper	Addgene Cat# 119864
pGEM-myr-Citrine-SV40pA-FKF	This paper	Addgene Cat#119865
pGEM-myr-eGFP-SV40pA-FKF	This paper	Addgene Cat#119866
pGEM-myr-tdTomato-SV40pA-FKF	This paper	Addgene Cat#119867
pGEM-NLS-Cre-2A-mCherry-FKF	This paper	Addgene Cat#119868
BAC clone DKEYP-79F12	https://www.sourcebioscience.com	DKEYP-79F12
BAC clone DKEYP-117G5	https://www.sourcebioscience.com	DKEYP-117G5
BAC clone CH73-157N22	https://www.sourcebioscience.com	CH73-157N22

**Software and Algorithms**		

STAR v2.4.2a	Dobin et al. (2013)	https://github.com/alexdobin/STAR
samtools v1.3	(Li et al., 2009)	http://htslib.org/
FeatureCounts v1.6.2	Liao et al. (2014)	http://bioinf.wehi.edu.au/featureCounts
velocyto v0.17.13	La Manno et al., 2018	http://velocyto.org/
R v3.4.3	R Core Team	https://www.r-project.org/
scater v1.6.3	McCarthy et al., 2017	https://bioconductor.org/packages/release/bioc/html/scater.html
scde v1.99.4	Fan et al. (2016)	http://hms-dbmi.github.io/scde/index.html
edgeR v3.20.9	Robinson et al., 2010	https://bioconductor.org/packages/release/bioc/html/edgeR.html
velocyto.R v0.5	La Manno et al., 2018	https://github.com/velocyto-team/velocyto.R
monocle v2.6.3	Qiu et al., 2017	https://github.com/cole-trapnell-lab/monocle-release
topGO v2.30.1	(Alexa and Rahnenfuehrer, 2019)	http://bioconductor.org/packages/release/bioc/html/topGO.html
Rtsne v0.13	Krijthe, 2015	https://github.com/jkrijthe/Rtsne
ggplot2 v 2.2.1	(Wickham, 2016)	https://ggplot2.tidyverse.org/
IGV v2.5.0	(Robinson et al., 2011)	https://software.broadinstitute.org/software/igv/

### Lead Contact and Materials Availability

Further information and requests for resources and reagents should be directed to and will be fulfilled by the Lead Contact, Paul Riley (paul.riley@dpag.ox.ac.uk). Requests for zebrafish transgenic lines should be directed to Tatjana Sauka-Spengler (tatjana.sauka-spengler@imm.ox.ac.uk). All plasmids generated in this study are readily available from Addgene (www.addgene.org/Tatjana_Sauka-Spengler/ pGEM-BirA-2A-H2B-Dendra2-SV40pA-FKF, Cat#119864; pGEM-myr-Citrine-SV40pA-FKF, Cat#119865; pGEM-myr-eGFP-SV40pA-FKF, Cat#119866; pGEM-myr-tdTomato-SV40pA-FKF Cat#119867; pGEM-NLS-Cre-2A-mCherry-FKF, Cat#119868).

### Experimental Model and Subject Details

For this study, both females and males of transgenic and wildtype zebrafish strains were used. Animals used for breeding were between 3 and 24 months old. Zebrafish embryos/larvae that were used for experiments were raised to an age of up to 7 days post fertilisation (dpf). Larvae were euthanised and analysed shortly before reaching an age of 5dpf (free-feeding) during all experiments for which the experimental timepoint is stated as “5dpf” or “120hpf” in text or figures. Fish were kept at a 14 hours light, 10 hours dark cycle and fed four times a day. All animal experiments were performed under a Home Office Licence according to the Animals Scientific Procedures Act 1986, UK, and approved by the local ethics committee.

#### Zebrafish Lines

Published transgenic reporter lines used in this study were: *TgBAC(tcf21:DsRed2)*^*pd37*^ (Kikuchi et al., 2011), *TgBAC(tbx18:DsRed2)*^*pd22*^ (Kikuchi et al., 2011), *TgBAC(cryaa:EGFP,tcf21:Cre-ERT2)*^*pd42*^ (Kikuchi et al., 2011), *Tg(wt1b:eGFP)*^*li1*^ (Perner et al., 2007), *Tg(myl7:eGFP)*^*f1*^ (Huang et al., 2003), *Tg(kdrl:GFP)*^*s843*^ (Beis et al., 2005), *Tg(gata1a:DsRed)* (Traver et al., 2003), *Tg(ptprc/CD45:DsRed)*^*sd3*^ (Bertrand et al., 2008), *Tg(–3.5ubb:loxP-EGFP-loxP-mCherry)* [*Tg(ubi:Switch)*] (Mosimann et al., 2011) and *Tg(ubb:loxPAmCyanSTOPloxPZsYellow)* [*Tg(ubi:CSY)*] (Zhou et al., 2011). *Tg(tcf21:DsRed2; myl7:eGFP)* and *Tg(kdrl:GFP; gata1a:DsRed)* double transgenic lines were generated by natural mating.

### Method Details

#### Generation of Transgenic Zebrafish Lines

To generate TgBAC(tcf21:myr-tdTomato)^ox181^, TgBAC(tcf21:H2B-Dendra2)^ox182^, TgBAC(tcf21:myr-eGFP)^ox183^, TgBAC(tbx18:myr-eGFP)^ox184^, TgBAC(tbx18:myr-Citrine)^ox185^, TgBAC(wt1b:H2B-Dendra2)^ox186^ and TgBAC(wt1b:Cre-2A-mCherry)^ox142^ we used a BAC recombineering approach (Bussmann and Schulte-Merker, 2011). Dendra2, tdTomato, eGFP and Citrine fluorophore sequences were linked to 5’ histone 2b (H2B) or 2x myristoylation (myr) sequences (Dempsey et al., 2012, Zacharias et al., 2002). pGEM H2B-Dendra2-FRT-Kan-FRT, pGEM myr-tdTomato-FRT-Kan-FRT, pGEM myr-eGFP-FRT-Kan-FRT, pGEM myr-Citrine-FRT-Kan-FRT and pGEM Cre-2A-mCherry-FRT-Kan-FRT recombination donor constructs were generated by amplifying H2B-Dendra2, myr-tdTomato, myr-eGFP, myr-Citrine and Cre-2A-mCherry cassettes using Herculase II fusion DNA polymerase (Agilent Technologies) and cloning them into the donor plasmid (#89890, Addgene) using InFusion (InFusion HD Cloning kit, Clontech). BACs used were DKEYP 79F12 (tcf21), DKEYP 117G5 (tbx18) and CH73 157N22 (wt1b). Donor constructs were inserted into the first coding exon of tcf21, tbx18 or wt1b BACs via bacterial homologous recombination as previously described (Trinh et al., 2017). Primers used are listed in Table S1. The corresponding constructs used for amplification of donor fragments flanked with recombinant arms are available from Addgene (#119864 - #119868). BACs were injected into one-cell zygotes and integrated into the genome via Tol2-mediated recombination. TgBAC(tcf21:myr-tdTomato;tbx18:myr-eGFP;wt1b:H2B-Dendra2)^ox187^ was generated by two consecutive rounds of breeding from TgBAC(tcf21:myr-tdTomato)^ox181^, TgBAC(wt1b:H2B-Dendra2)^ox186^ and TgBAC(tbx18:myr-eGFP)^ox184^. Images of euthanised larvae were obtained using a LSM780 confocal microscope (ZEISS) and a 20x objective.

#### Larval Heart Isolation, Dissociation and FAC-Sorting

Larvae were euthanised using tricaine methanesulfonate (MS-222) and larval hearts were isolated following a published protocol (Burns and MacRae, 2006), using a 21-gauge needle for disruption. This procedure recovered around 50% of the larval hearts. Hearts were dissociated using 15mg/ml collagenase (C8176, Sigma Aldrich) in 0.05% trypsin solution at 30°C for 14mins. Single cells were sorted on a BD FACS-Aria Fusion into Smart-seq2 lysis buffer dispensed in 96 well plates. 7-AAD cell viability dye was used to exclude non-viable cells during FACS. 288 fluorescent cells were purified from *TgBAC(tcf21:H2B-Dendra2)*^*ox182*^ (300 hearts), 96 fluorescent cells from *TgBAC(tbx18:myr-eGFP)*^*ox184*^ (180 hearts) and 96 fluorescent cells from *TgBAC(wt1b:H2B-Dendra2)*^*ox186*^ (130 hearts). Additionally, 31 GFP positive cells were collected from *Tg(myl7:eGFP)*^*f1*^ (40 hearts), 40 GFP positive cells and 20 DsRed fluorescent cells from *Tg(kdrl:GFP; gata1a:DsRed)* (65 hearts), 96 non-fluorescent cells from *Tg(tcf21:DsRed2; myl7:eGFP)* x *Tg(kdrl:GFP; gata1a:DsRed)* (100 ventricles) and 96 cells from 50 wildtype hearts. Cells from *TgBAC(tcf21:H2B-Dendra2)*^*ox182*^ were sorted in four separate FACS sessions, GFP positive and DsRed positive cells from *Tg(kdrl:GFP; gata1a:DsRed)* and GFP positive cells from *Tg(myl7:eGFP)*^*f1*^ were sorted in one session, cells in all other conditions were sorted separately in one session each. Cells sorted in separate FACS sessions were processed separately during cDNA synthesis, cells sorted in one session were processed as one batch. Three empty wells were processed within the *kdrl*:GFP, *gata1a*:DsRed batch. During library preparation and sequencing, cDNA samples from *tbx18*:myr-eGFP, *wt1b*:H2B-Dendra2, *kdrl*:GFP, *gata1a*:DsRed and *myl7*:eGFP conditions were scrambled and processed as two new batches, *tcf21*:H2B-Dendra2,*myl7*:eGFP,*kdrl*:GFP,*gata1a*:DsRed quadruple negative and wildtype conditions were processed in one batch, all other conditions were processed separately.

During *sema3fb* knockout experiments, 241 Citrine positive single cells were purified from 300 *TgBAC(tbx18:myr-Citrine)*^*ox185*^ hearts in three batches and sorted into 96-well plates containing lysis buffer with protease (Qiagen). cDNA was synthesised separately for each batch. Batches were combined during library preparation and sequencing.

#### Single Cell Library Preparation and Sequencing

Single cells were processed following the Smart-seq2 protocol (Picelli et al., 2013) to reverse transcribe poly-adenylated RNA. cDNA was then amplified with 20 PCR cycles. In two of the batches of cells sorted from *TgBAC(tcf21:H2B-Dendra2)*^*ox182*^, all cDNA samples were taken further into library preparation and sequencing. However, only a low percentage of these samples passed the quality control after sequencing (Figure S2F). Therefore, in all other batches the amount of synthesized cDNA was quantified using the Quant-iT PicoGreen dsDNA Assay (ThermoFisher). cDNA quality of 11 samples sampling the whole range of the obtained PicoGreen results was tested on an Agilent 2100 Bioanalyzer (Agilent Technologies). The Bioanalyzer results were used to determine a quality cut-off PicoGreen value. Only cDNA samples with a PicoGreen value above the cut-off were taken further into library preparation. Final sequencing libraries were prepared using the Nextera XT DNA Library Preparation Kit (FC-131-1024, Illumina) and 75bp paired-end sequencing reads were generated on a NextSeq500 platform (Illumina). The number of sequenced cells for each condition can be found in Figure S2.

#### KO *sema3fb* Single Cell Library Preparation and Sequencing

Single cells isolated from hearts of euthanised 5dpf *sema3fb* knockout larvae were processed adapting the TARGET-seq protocol (Rodriguez-Meira et al., 2019). Similar to TARGET-seq, single cells were subjected to a mild protease digestion to release genomic DNA. In addition to template-switching and oligo-dT primers, primers specific to *sema3fb* mRNA were used to enforce reverse transcription of *sema3fb* during first-strand synthesis. Primers specific for *sema3fb* cDNA were added during cDNA amplification. Additionally, genomic DNA primers binding intronic sequences surrounding the target sites of *sema3fb* sgRNA1 (exon 2) and sgRNA4 (exon 10) were added into the same PCR mix. Single cell cDNA was amplified using 24 PCR cycles. Primer specificity was validated against zebrafish RefSeq mRNA and genomic DNA assemblies in PrimerBlast. Final sequencing libraries were prepared using the Nextera XT DNA Library Preparation Kit (Illumina) and 130 libraries were sequenced on a NextSeq500 platform (Illumina), generating 75bp paired-end sequencing reads. A subset of 40 samples was re-sequenced for increased coverage of the *sema3fb* locus, generating 75bp single-read sequencing reads. Primer sequences can be found in Table S4.

#### KO *cxcl12a* Single Embryo Library Preparation and Sequencing

Zygotes were injected with two sgRNAs targeting AmCyan (control, n=3) or sgRNA1 and sgRNA3 targeting *cxcl12a* (KO *cxcl12a*, n=6), single larvae were euthanised using MS-222 shortly before 5dpf and RNA was extracted using the RNaqueous Micro Kit (Ambion). Approximately 50ng of purified RNA was processed with SMARTScribe reverse transcriptase (Clontech), using 1.5μM *cxcl12a* specific exonic reverse primer and 5μM Smart-seq2 template-switching oligo. cDNA was amplified using SeqAmp polymerase (Clontech), 0.33 μM Smart-seq2 ISPCR primers and 6 PCR cycles. Final sequencing libraries were prepared using the Nextera XT DNA Library Preparation Kit (Illumina) and libraries were sequenced on a NextSeq500 platform (Illumina), generating 75bp paired-end sequencing reads. All samples were re-sequenced for increased coverage, generating 75bp single-read sequencing reads. The *cxcl12a* primer sequence can be found in Table S4.

#### Hybridisation Chain Reaction

Larvae were euthanised using MS-222 and fixed in 4% paraformaldehyde (PFA) overnight at 4°C. Subsequently, larvae were stored at -20°C in methanol. Hybridisation chain reaction (HCR) v3.0 (Choi et al., 2018) was performed following a protocol by Choi et al. Briefly, larvae were permeabilized using 30μg/ml proteinase K for 45 minutes at room temperature, post-fixed in 4% PFA and incubated overnight at 37°C in 30% probe hybridisation buffer containing 2pmol of each probe mixture. Excess probes were washed off with 30% probe wash buffer at 37°C and 5xSSCT at room temperature and larvae were incubated overnight at room temperature in amplification buffer containing 15pmol of each fluorescently labelled hairpin. Following HCR, larvae were incubated with Hoechst reagent (1:1000, 5xSSCT) for 30 minutes at room temperature. Probe sequences were designed by the manufacturer. Probe sets used were: dr_tcf21-B1, dr_tbx18-B2, dr_wt1b-B3, dr_myl7-B5, dr_adma-B3, dr_jam2b-B4, dr_elnb-B4, dr_cldn11a-B2, dr_tgm2b-B4, dr_sema3fb-B5, dr_nrp1a-B4, dr_nrp2a-B3, dr_cxcl12a-B3, dr_cxcr4b-B5, dr_ptprc-B4, EGFP-B4, Dendra2-B5, tdTomato-B5, mCherry-B1. During BAC reporter line validation, HCR was performed using probes specifically targeting exon 1 of *tcf21*, *tbx18* or *wt1b* (dr_tcf21_exon1-B1, dr_tbx18_exon1-B2, dr_wt1b_exon1-B3), which was absent from the BAC reporter constructs. Images were obtained using a LSM780 confocal microscope (ZEISS) and a 40x objective. Contrast and brightness were adjusted separately for each colour channel.

#### Immunocytochemistry

Larvae were euthanised using MS-222 and fixed in 4% PFA for 45 minutes at room temperature. After washing, larvae were blocked using 5% goat serum (PBS, 0.5% Triton, 2% DMSO) for 1 hour at room temperature. Primary antibodies used were: chicken anti-GFP (ab13970, abcam, 1:500), rabbit anti-DsRed (632496, Clontech, 1:500), mouse anti-Dendra2 (TA180094, clone OTI1G6, Origene, 1:500), mouse anti-Mlck (M7905, Sigma-Aldrich, 1:500) and rabbit anti-cxcr4b (GTX132244, Genetex, 1:300), added overnight at 4°C. Secondary antibodies and Hoechst reagent were used at 1:1000 dilution and added for 2 hours at room temperature. Images were obtained using a LSM780 confocal microscope (ZEISS) and a 40x objective, or a MVX10 fluorescence stereo microscope (Olympus). Contrast and brightness were adjusted separately for each colour channel.

#### EdU Staining

Larvae were incubated in embryo medium containing 1mM EdU and 1% DMSO. During *sema3fb* knockout experiments, larvae were incubated for 18 hours, from 102hpf to shortly before 120hpf. During *tgm2b* knockout experiments, larvae were incubated for 11 hours, from 66hpf until 77hpf. KO *sema3fb* larvae were stained by immunocytochemistry against GFP, followed by detection of EdU using the Click-iT Alexa Fluor 647nm kit (ThermoFisher) for 2 hours at room temperature. KO *tgm2b* larvae were immuno-stained against Dendra2, followed by EdU detection.

#### Lineage Tracing

4-hydroxytamoxifen (4-OHT) was added to the embryo medium at a final concentration of 10μM, from a 1mM stock solution made in 100% ethanol. Embryos were incubated with 4-OHT from 10 hours-post-fertilisation (hpf) or 43hpf. For embryos incubated with 4-OHT from 10hpf, the 4-OHT containing medium was renewed at 2dpf and at 4dpf. For embryos incubated from 43hpf, the 4-OHT containing medium was replaced at 4dpf. Embryos were grown until shortly before 5dpf.

#### Transient Cas9-Mediated Gene Knockout

sgRNAs against the coding region of the target gene were designed to have a high Cas9-mediated nuclease cutting efficiency, a low number of genomic off-target sites and a target site proximal to the recognition site of a restriction enzyme. sgRNA primers (see Table S2) were annealed and the product inserted via Golden Gate cloning into a U6a mini-vector containing a tracrRNA backbone and Ds transposon sequences (Addgene #119069). sgRNA vectors were first injected individually to test their Cas9-mediated nuclease cutting efficiency. Typically, 30pg of vector was injected into one-cell stage zygotes, together with 10pg Ac mRNA and 160pg Cas9 mRNA. For actual gene editing experiments, two sgRNA vectors were co-injected at an amount of 30-38pg each. Control embryos were injected with two sgRNA vectors targeting mCherry (KO *tgm2b*, *sema3fb*) or AmCyan (KO *cxcl12a*).

#### Cas9 Activity Efficiency Test

One cell stage zygotes were injected with a single sgRNA vector, Ac mRNA and Cas9 mRNA. Genomic DNA from single euthanised embryos was isolated at 1dpf and the genomic region surrounding the sgRNA target site (200-400bp) was amplified via PCR (see Table S3 for primers used). Half the volume of each PCR product was digested with a restriction enzyme that had a recognition site overlapping or adjacent to the sgRNA target site.

#### Generation of Stable Mutant Zebrafish Lines

We injected *tgm2b* sgRNA2 vector, Ac mRNA and Cas9 mRNA into one cell stage *TgBAC(tcf21:H2B-Dendra2)*^*ox182*^ x *TgBAC(tbx18:myr-Citrine)*^*ox185*^ zygotes, reared fluorescent fish to adulthood and screened for germline transmission of editing events to generate *tgm2b*^*38ins/38ins*^ *mutant in the Tg(tcf21:H2B-Dendra2;tbx18:myr-Citrine)* background (ox188) and *tgm2b*^*7del1sub/7del1sub*^ *mutant in Tg(tcf21:H2B-Dendra2;tbx18:myr-Citrine)* background (ox189). Similarly, we injected *cxcl12a* sgRNA1 vector into *Tg(ptprc/CD45:DsRed)*^*sd3*^ x *TgBAC(tcf21:myr-eGFP)*^*ox183*^ zygotes to generate *cxcl12a*^*15del/15del*^ in *Tg(ptprc/CD45:DsRed; tcf21:myr-eGFP)* background (ox190). To identify mutants, embryos were fin-clipped and extracted genomic DNA was PCR amplified using the primers:

*tgm2b*_sgRNA2_for GGGAGGAAGCGTTCAGATACTA and

*tgm2b*_sgRNA2_rev ATACCGCACTGGCAGACAC (wildtype PCR product size 61bp), or *cxcl12a*_sgRNA1_for GCAAACATGGATCTCAAAGTGA and

*cxcl12a*_sgRNA1_rev TTAACTTACCGTTGGAAATCGG (wildtype PCR product size 82bp). The sizes of the PCR products were then determined using gel electrophoresis. Mutant genotypes were characterised via Sanger sequencing.

#### Bioinformatic Processing

##### Single Cell RNA Sequencing Data Analysis

Transcriptomic data was mapped to the zebrafish reference genome (GRCz10-91) using the STAR gapped aligner (Dobin et al., 2013). Duplicate reads were removed and reads were summarized using featureCounts (Liao et al., 2014). FeatureCounts files containing data from different sequencing batches were subsequently concatenated. Quality control of the single cell read counts was done using the Scater R package (McCarthy et al., 2017). Libraries that had a size more than 3 median absolute deviations (MADs) below the median of the whole dataset were excluded from the analysis. Furthermore, libraries were excluded from the analysis if the number of expressed genes was more than 3 MADs below the median of the whole dataset, or if the percentage of counts representing ERCC spike-in features was more than 3 MADs above the median of the whole dataset. Genes with an average expression across all cells of below 0.1 counts as well as mitochondrial genes were excluded from analysis. Quality control plots can be found in Figure S2. The cleaned dataset was further processed using Pagoda routines in the scde package (Fan et al., 2016, Kharchenko et al., 2014). Genes annotated to cell cycle related gene ontology terms were excluded from the dataset. Gene enrichment in the single cell clusters was analysed with scde. Counts were transformed into FPKM expression values with the rpkm() command in the edgeR package (Robinson et al., 2010) and heatmaps drawn using pheatmap. The Rtsne package (Krijthe, 2015) was used to plot t-SNE representations of the dataset that were based on 2376 highly variable genes. Gene ontology term analysis was performed with the R topGO package (Alexa and Rahnenfuehrer, 2019), using enriched genes with cZ>2. RNA velocity was analysed using the Velocyto R package (La Manno et al., 2018) and pseudotime analysis was performed using the Monocle 2 package (Qiu et al., 2017).

##### KO *sema3fb* Single Cell RNA Sequencing Data Analysis

To identify single cell clusters, sequencing data from *sema3fb* knockout samples was processed using the Pagoda pipeline as described above. FPKM values were computed using the rpkm() function in edgeR. Raw counts for *sema3fb* were generated using featureCounts and a custom gene annotation in which exons targeted by *sema3fb* sgRNAs and amplified from genomic DNA (exons 2 and 10) were absent. Raw counts were then normalised to sample library size; no *sema3fb* FPKM values were determined since effective *sema3fb* transcript lengths could not reliably be computed for each single cell sample. To analyse genome editing, STAR mapped sequencing reads (without duplicates) were inspected using the Integrative Genomics Viewer (Robinson et al., 2011). For re-sequenced samples, the two BAM files were merged using samtools (Li et al., 2009). 14 samples containing reads covering at least one of the sgRNA target sites (sgRNA1: chr11:35307703, sgRNA4:chr11:35213000) were analysed in further detail. For these samples, sequences of reads spanning the sgRNA target sites were compared to the genomic reference sequence and to reads of wildtype *sema3fb* positive cells from the original scRNASeq dataset. Samples with read sequences that differed from these reference sequences were classified as genome-edited. Editing types detected were grouped into insertions/deletions (indels), basepair substitutions and large deletions over 10kb in length. The effect of genome editing on the sema3fb protein open reading frame (ORF) was analysed using SnapGene Viewer (SnapGene) and the lengths of edited sema3fb ORFs were normalised to wildtype sema3fb length (791aa). IGV was used to generate Sashimi plots showing splicing of *sema3fb*. In 9 samples that contained spliced reads for more than one *sema3fb* splice junction, the number of reads indicating aberrant *sema3fb* splicing was normalised against the total number of *sema3fb* splicing reads and plotted. 12 non *sema3fb* knockout *sema3fb* expressing single cell transcriptome samples were analysed similarly.

##### KO *cxcl12a* RNA Sequencing Data Analysis

Sequencing reads were mapped with STAR to GRCz10-91, duplicate reads removed and the *cxcl12a* locus analysed using IGV. Read sequences at the sgRNA target sites (chr13:30674045 sgRNA1, chr13:30676177 sgRNA3) were compared to the reference genome. The numbers of reads that contained indels or substitutions or that indicated aberrant splicing from exon 1 to exon 3 were normalised against the total number of reads spanning the respective sgRNA target site. Sashimi plots were generated with IGV and the number of reads that indicated aberrant splicing from exon 1 to exon 3 was normalised against the total number of reads splicing from exon 1. The cxcl12a ORFs resulting from genome editing were analysed in SnapGene Viewer and their lengths were normalised against the wildtype cxcl12a length (99aa). FPKM values were computed manually. To determine the effective transcript length (as produced by Smart-seq2 template-switching oligo and *cxcl12a* cDNA reverse primer) for each sample, the amount of *cxcl12a* transcripts not containing exon 2 (length 1224bp) was weighted against the amount of wildtype transcript (length 1342bp) using the previously computed ratio of aberrant splicing.

### Quantification and Statistical Analysis

Statistical details of experiments can be found in the figures, figure legends and main text, including p-values and numbers of animals analysed. Statistical analyses were performed in R. Plots were generated with ggplot2 (Wickham, 2016) in R. For the scRNA-seq GO-term enrichment analysis, significance was calculated using Fisher's exact test with Bonferroni correction for multiple testing. For data shown in Figures 4–6, significance was calculated using Welch’s t-test. Some plots include box and whiskers plots (in the style of Tukey), indicating median and first/third quartiles.

### Data and Code Availability

Raw and processed sequencing data generated in this study was submitted to GEO (accession number GSE121750).

## Supplemental Information

Document S1. Figures S1–S7 and Tables S1–S4

Document S2. Article plus Supplemental Information
