# Supplementary material for: Functional Heterogeneity within the Developing Zebrafish Epicardium
Source: Dev Cell. 2020 Mar 9;52(5):574–590.e6. doi: 10.1016/j.devcel.2020.01.023 (PMC7063573; doi:10.1016/j.devcel.2020.01.023)
Supplement: Document S1. Figures S1–S7 and Tables S1–S4 [file mmc1.pdf]

Developmental Cell, Volume *52*

Supplemental Information

Functional Heterogeneity within the Developing Zebrafish Epicardium

Michael Weinberger, Filipa C. Simões, Roger Patient, Tatjana Sauka-Spengler, and Paul R. Riley

Figure S1.

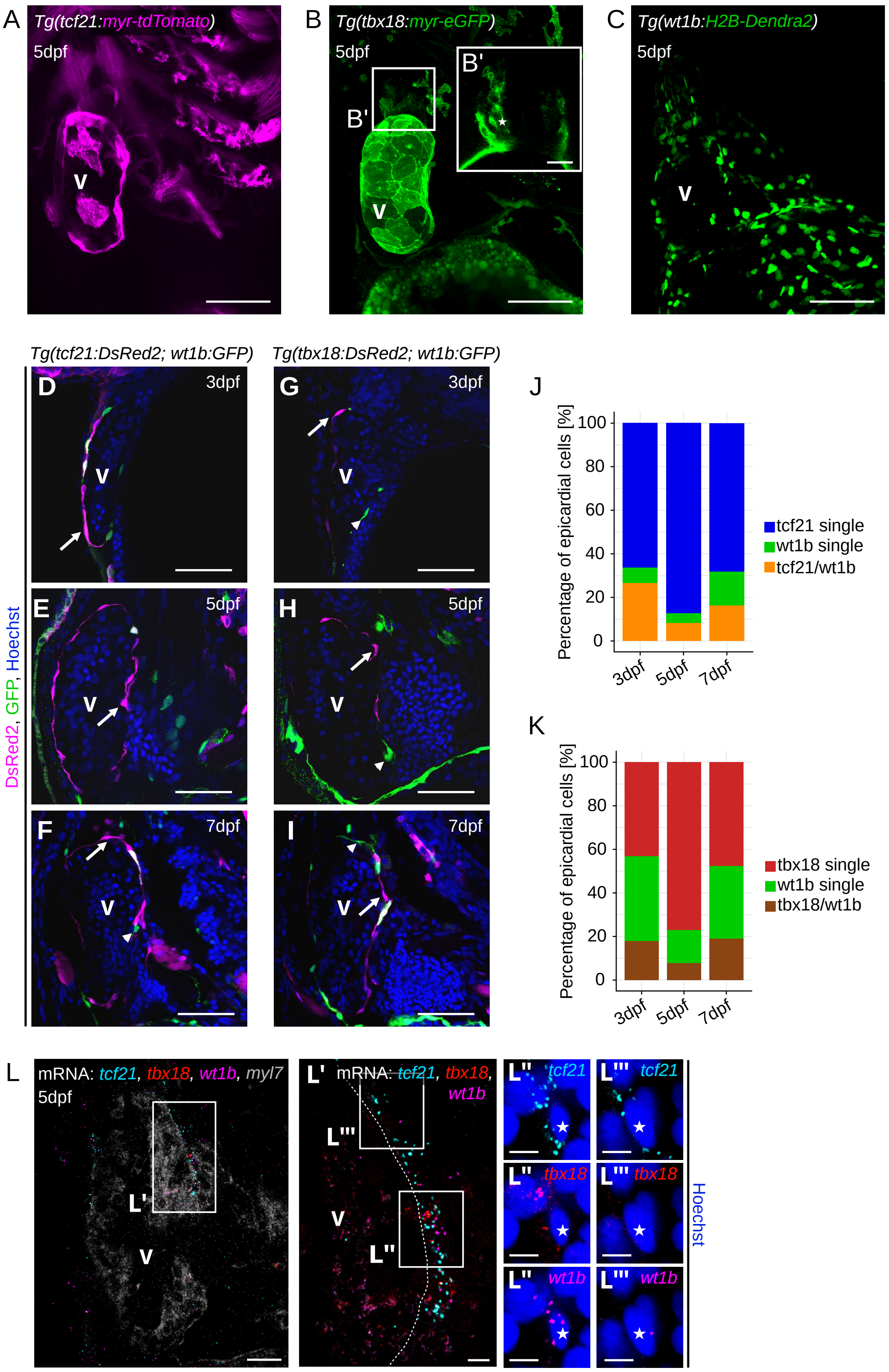

A
Tg(tcf21:myr-tdTomato)
5dpf
V
B
Tg(tbx18:myr-eGFP)
5dpf
B'
V
C
Tg(wt1b:H2B-Dendra2)
5dpf
V
Tg(tcf21:DsRed2; wt1b:GFP)
Tg(tbx18:DsRed2; wt1b:GFP)
DsRed2, GFP, Hoechst
D 3dpf
E 5dpf
F 7dpf
G 3dpf
H 5dpf
I 7dpf
J
Percentage of epicardial cells [%]
100
80
60
40
20
0
3dpf
5dpf
7dpf
tcf21 single
wt1b single
tcf21/wt1b
K
Percentage of epicardial cells [%]
tbx18 single
wt1b single
tbx18/wt1b
L
mRNA: tcf21, tbx18, wt1b, myl7
5dpf
L'
L''
L'''
mRNA: tcf21, tbx18, wt1b
tcf21
tbx18
wt1b
Hoechst

Figure S1. Heterogeneous expression of *tcf21*:DsRed2, *tbx18*:DsRed2 and *wt1b*:GFP in the developing zebrafish epicardium. Related to Figure 1.

(A-C) Projections of *TgBAC(tcf21:myr-tdTomato)* (A), *TgBAC(tbx18:myr-eGFP)* (B) and *TgBAC(wt1b:H2B-Dendra2)* (C) hearts at 5dpf. (B') Magnified single optical section of the boxed area in B showing the presence of *tbx18*:myr-eGFP^{+} cells within the BA (asterisk). (D-F) Projections of the heart region in *tcf21*:DsRed2^{+}, *wt1b*:GFP^{+} larvae at 3dpf (D), 5dpf (E) and 7dpf (F). Many epicardial cells are DsRed2^{+}, GFP^{-} (arrows), at 7dpf some epicardial cells are DsRed2^{-}, GFP^{+} (arrowhead). (G-I) Projections of the heart region in *tbx18*:DsRed2^{+}, *wt1b*:GFP^{+} larvae at 3dpf (G), 5dpf (H) and 7dpf (I). Many epicardial cells are DsRed2^{+}, GFP^{-} (arrows) and DsRed2^{-}, GFP^{+} (arrowheads). (J) Relative quantification of the epicardial combinations of *tcf21*:DsRed2 and *wt1b*:GFP. 3dpf n=6, 5dpf n=9, 7dpf n=3. (K) Relative quantification of the epicardial combinations of *tbx18*:DsRed2 and *wt1b*:GFP. 3dpf n=6, 5dpf n=10, 7dpf n=6. (L) Visualisation of *tcf21* (cyan), *tbx18* (red) and *wt1b* (magenta) and *myl7* (grey) mRNA at 5dpf. (L'-L''') Cell nuclei (asterisks) in the epicardial region in close proximity to *tcf21*, *tbx18* and *wt1b* (L''), as well as in close proximity to *tcf21* and *wt1b*, but not *tbx18* (L'''). Scale bars in A-C,D-I: 50µm, L: 20µm, B': 10µm, L'-L''': 5µm. Colour channels in microscopy images were adjusted separately for brightness/contrast. A-C are projections, D-I,L are single optical sections. V=ventricle.

Figure S2.

A

Line sorted from	Wildtype	*Tg(tcf21:H2B-Dendra2)*	*Tg(tbx18:myr-eGFP)*	*Tg(wt1b:H2B-Dendra2)*	*Tg(myl7:eGFP)*	*Tg(kdrl:GFP;gata1a:DsRed)*	*Tg(kdrl:GFP;gata1a:DsRed)*	*Tg(tcf21:DsRed2; myl7:eGFP) x Tg(kdrl:GFP; gata1a:DsRed)*	Empty well
Fluorescent cells sorted	No	Yes	Yes	Yes	Yes	Yes *kdrl*:GFP	Yes *gata1a*:DsRed	No	No
Number of cells sequenced	52	228	52	50	14	15	2	48	3
Number of cells after QC	51	137	52	47	14	15	2	48	0
Proportion passed (%)	98	60	100	94	100	100	100	100	0

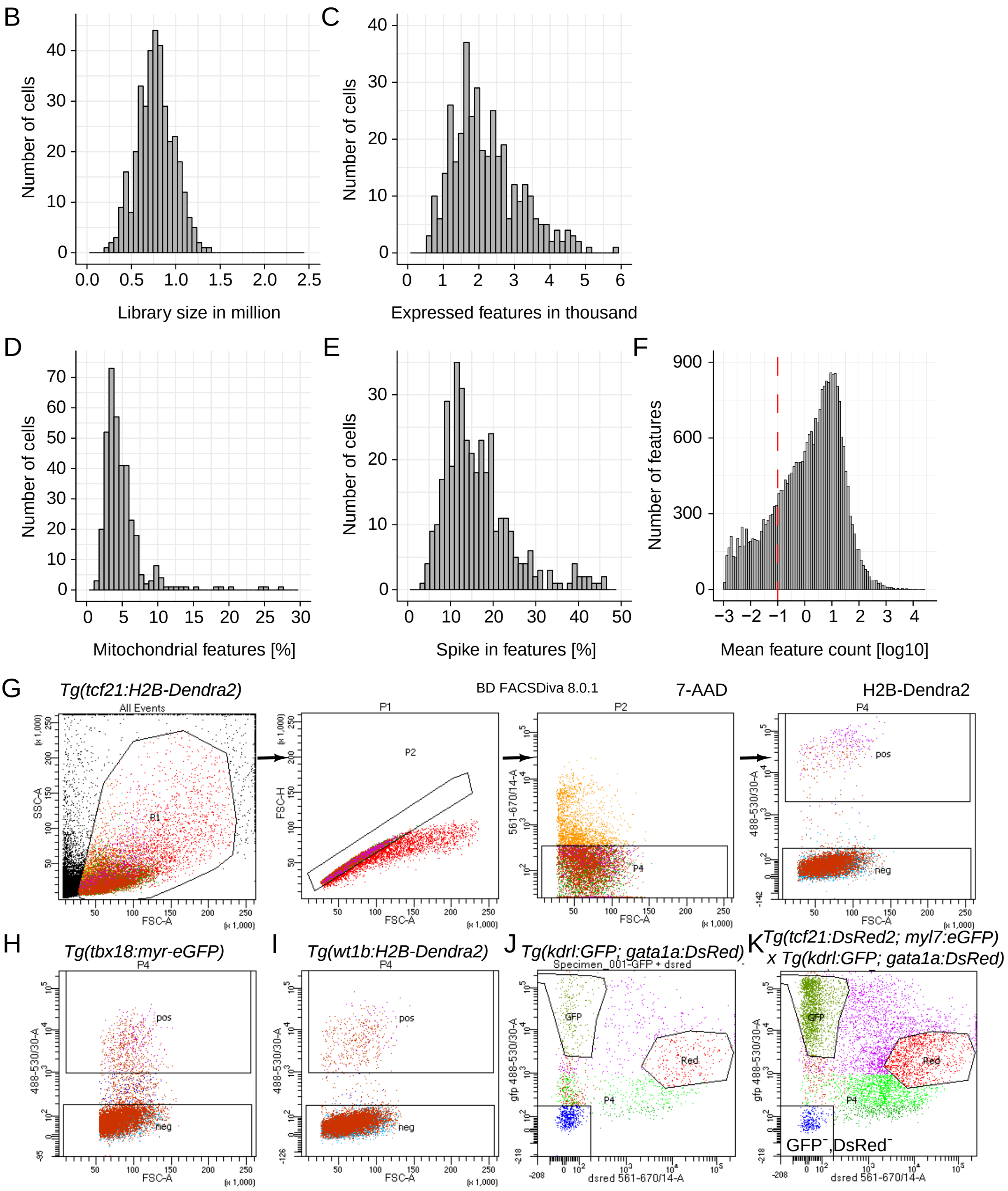

Figure S2. Quality control of the scRNA-seq dataset. Related to Figure 2.

(A) Number of single cells processed from each reporter/wildtype line to generate the scRNA-seq dataset. QC=quality control. (B-F) Histograms of library sizes (B), numbers of expressed features (C), percentage of mitochondrial reads on total reads (D), percentage of spike-in reads on total reads (E) and mean gene expression levels across cells (F). The dashed red line in F indicates the cut-off value of 0.1 read counts that was used to exclude genes with a lower mean expression level. (G) Representative FACS strategy to isolate fluorescent cells from reporter hearts (*TgBAC(tcf21:H2B-Dendra2)*). 7-AAD was used to exclude non-viable cells. (H-J) Flow cytometry profiles of *tbx18*:myr-eGFP$^+$ (H), *wt1b*:H2B-Dendra2$^+$ (I), *kdrl*:GFP/*gata1a*:DsRed$^+$ (J) cells. (K) FACS gating of quadruple negative cells in *Tg(tcf21:DsRed2;myl7:eGFP)* x *Tg(kdrl:GFP;gata1a:DsRed)* hearts.

Figure S3.

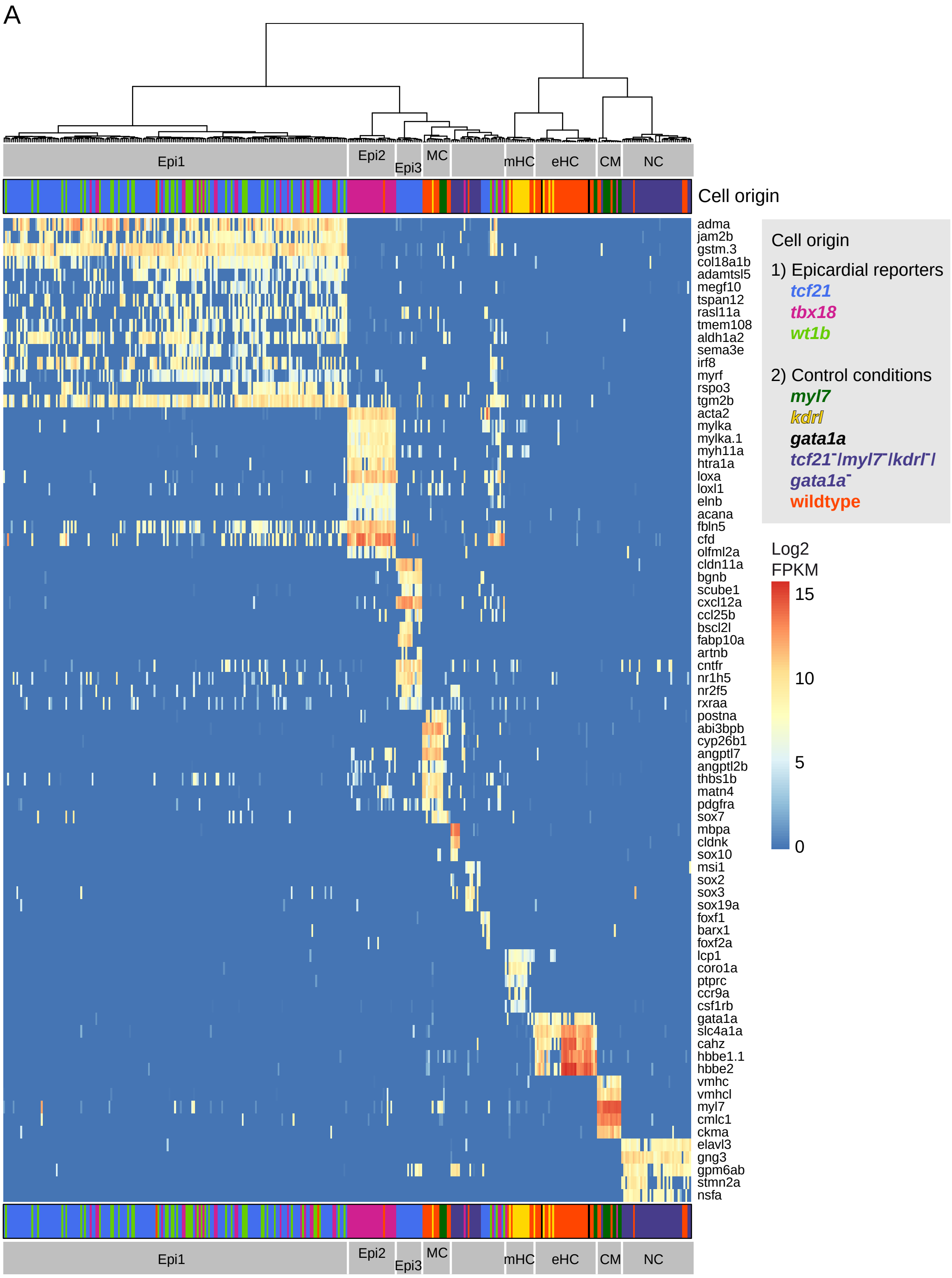
A
Epi1
Epi2
Epi3
MC
mHC
eHC
CM
NC
Cell origin
adma
jam2b
gstm.3
col18a1b
adamtsl5
megf10
tspan12
rasl11a
tmem108
aldh1a2
sema3e
irf8
myrf
rspo3
tgm2b
acta2
mylka
mylka.1
myh11a
htra1a
loxa
loxl1
elnb
acana
fbln5
cfd
olfml2a
cldn11a
bgnb
scube1
cxcl12a
ccl25b
bscl2l
fabp10a
artnb
cntfr
nr1h5
nr2f5
rxraa
postna
abi3bpb
cyp26b1
angptl7
angptl2b
thbs1b
matn4
pdgfra
sox7
mbpa
cldnk
sox10
msi1
sox2
sox3
sox19a
foxf1
barx1
foxf2a
lcp1
coro1a
ptprc
ccr9a
csf1rb
gata1a
slc4a1a
cahz
hbbe1.1
hbbe2
vmhc
vmhcl
myl7
cmlc1
ckma
elavl3
gng3
gpm6ab
stmn2a
nsfa
Cell origin
1) Epicardial reporters
tcf21
tbx18
wt1b
2) Control conditions
myl7
kdrl
gata1a
tcf21⁻/myl7⁻/kdrl⁻/
gata1a⁻
wildtype
Log2
FPKM
15
10
5
0

Figure S3. Marker gene expression across scRNA-seq clusters. Related to Figure 2.

(A) Cells were clustered in an unsupervised manner (columns). Shown are log2 transformed FPKM values. Cell cluster identity and colour-coded line origin of each cell are indicated at top and bottom of the heatmap. CM=cardiomyocytes, eHC=erythroid haematopoietic cells, mHC=myeloid haematopoietic cells, NC=neural cells, MC=mesenchymal cells.

Figure S4.

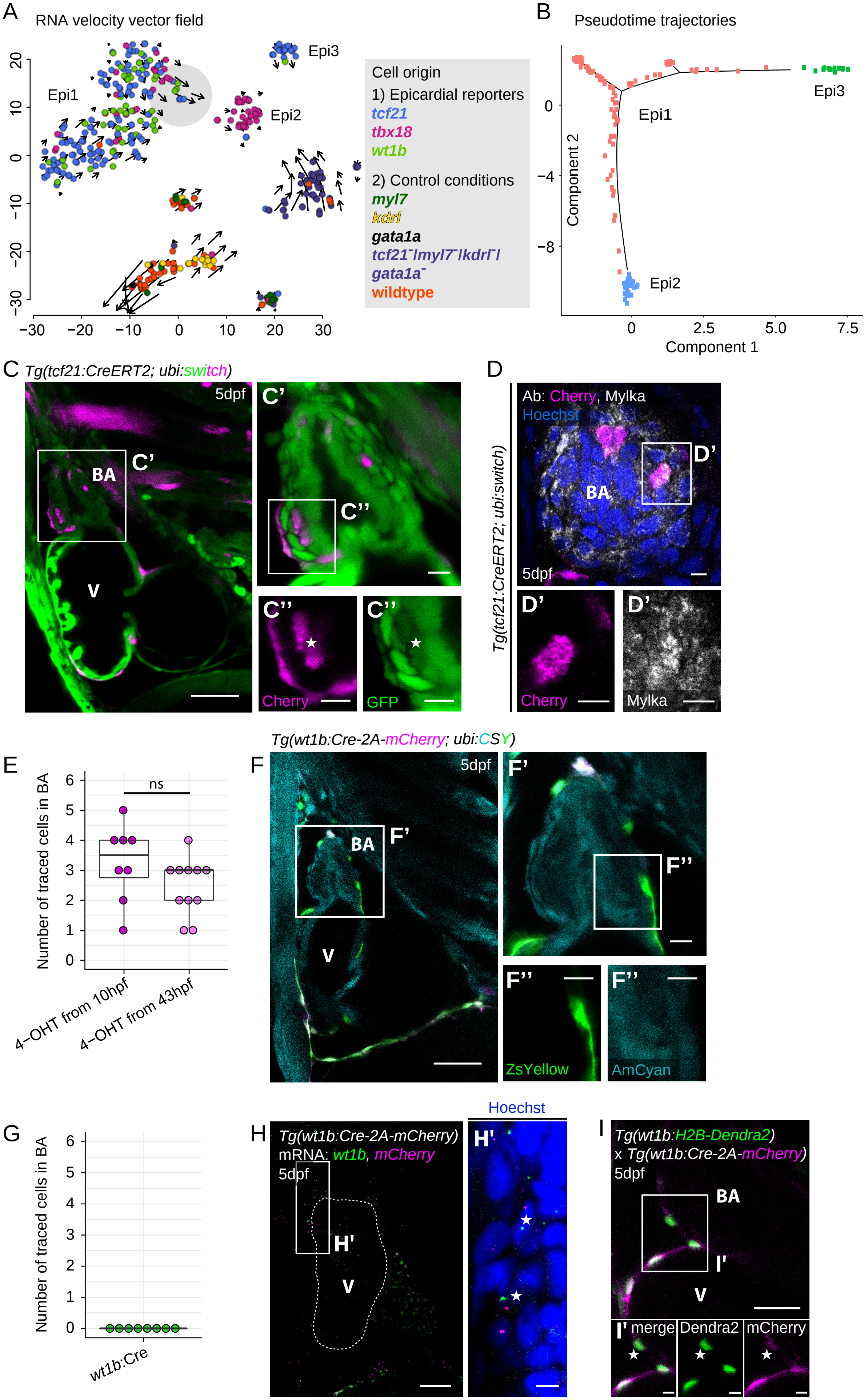

A
RNA velocity vector field
Epi1
Epi2
Epi3
Cell origin
1) Epicardial reporters
tcf21
tbx18
wt1b
2) Control conditions
myl7
kdrl
gata1a
tcf21⁻/myl7⁻/kdrl⁻/
gata1a⁻
wildtype
B
Pseudotime trajectories
Component 1
Component 2
C
Tg(tcf21:CreERT2; ubi:switch)
5dpf
BA
V
C'
C''
Cherry
GFP
D
Ab: Cherry, Mylka
Hoechst
Tg(tcf21:CreERT2; ubi:switch)
5dpf
BA
D'
Cherry
Mylka
E
Number of traced cells in BA
ns
4-OHT from 10hpf
4-OHT from 43hpf
F
Tg(wt1b:Cre-2A-mCherry; ubi:CSY)
5dpf
BA
V
F'
F''
ZsYellow
AmCyan
G
Number of traced cells in BA
wt1b:Cre
H
Tg(wt1b:Cre-2A-mCherry)
mRNA: wt1b, mCherry
5dpf
H'
V
Hoechst
I
Tg(wt1b:H2B-Dendra2)
x Tg(wt1b:Cre-2A-mCherry)
5dpf
BA
I'
V
merge
Dendra2
mCherry

Figure S4. Epi2 cells have a *tcf21* origin but never express *wt1b*. Related to Figure 3.

(A) Directionality of cell differentiation in the scRNA-seq dataset, as determined by the Velocyto R package. mRNA velocities are shown in a vector field that is projected onto the pre-calculated t-SNE plot. A group of cells in Epi1 that differentiates towards Epi2 is highlighted by grey background. (B) Pseudotime trajectories of Epi1-3 cells. Cell affiliation to Epi1-3 is indicated by colour. (C) 4-hydroxy-tamoxifen (4-OHT) mediated permanent colour labeling (magenta) in *tcf21*:Cre-ERT2, *ubi*:switch larvae at 5dpf. 4-OHT was added at 10hpf. (C',C'') *tcf21* lineage traced cells in the BA (asterisk, boxed area in C). (D) Antibody staining of Mylka and mCherry in the BA of a 5dpf *tcf21*:Cre-ERT2, *ubi*:switch larvae. (D') A *tcf21* lineage traced cell expressing Mylka (boxed area in D). (E) Absolute quantification of *tcf21*:Cre-ERT2 traced cells in the BA. Larvae were incubated with 4-OHT from either 10hpf or 43hpf and analysed at 5dpf. (F) Permanent colour labelling (green) in the heart region of a *wt1b*:Cre-2A-mCherry; *ubi*:CSY larva at 5dpf. (F',F'') Absence of *wt1b* lineage traced cells in the BA (boxed area in F). (G) Absolute quantification of the number of *wt1b*:Cre lineage traced cells within the BA. (H) mRNA staining of *wt1b* (magenta) and *mCherry* (green) in a *TgBAC(wt1b:Cre-2A-mCherry)* heart at 5dpf. (H') Nuclei (asterisks) surrounded by *wt1b* and *mCherry*. (I) Single optical section from a *TgBAC(wt1b:H2B-Dendra2)* x *TgBAC(wt1b:Cre-2A-mCherry)* heart at 5dpf. (I') Overlap of *wt1b*:H2B-Dendra2 and *wt1b*:Cre-2A-mCherry (asterisk). Scale bars in C,F: 50µm, H,I: 20µm, C',C'',D,D',F',F'': 10µm, H',I': 5µm. Colour channels in microscopy images were adjusted separately for brightness/contrast. C,D,F,H,I are single optical sections. Data represented as median, first and third quartiles (box). Significance was calculated using Welch's t-

test. V=ventricle, BA=bulbus arteriosus, Ab=antibody. E: n(4-OHT 10hpf)=8, n(4-OHT 43hpf)=11. G: n=8.

Figure S5.

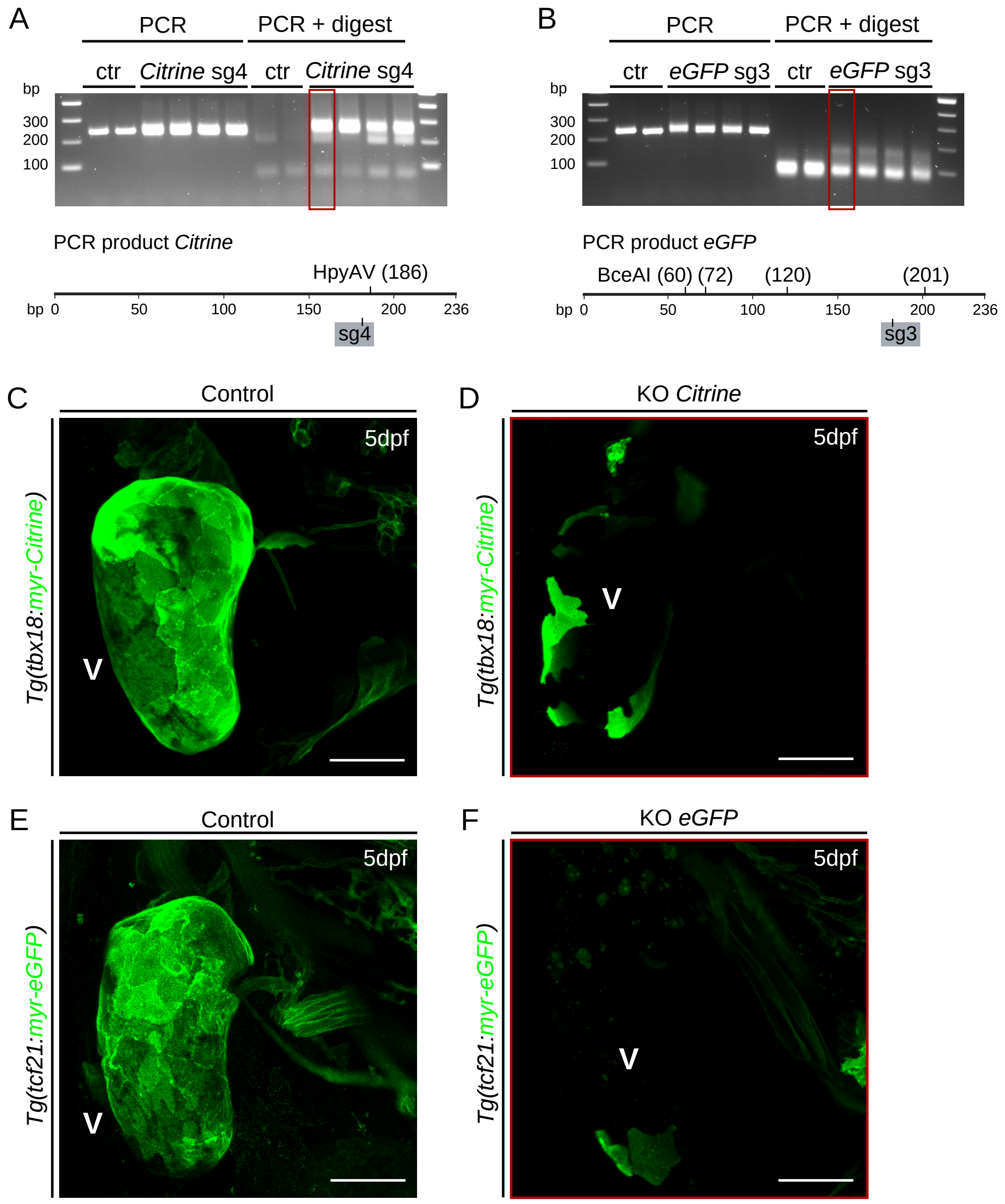
A
PCR
PCR + digest
ctr
Citrine sg4
ctr
Citrine sg4
bp
300
200
100
PCR product Citrine
HpyAV (186)
bp 0
50
100
150
200
236
sg4
B
PCR
PCR + digest
ctr
eGFP sg3
ctr
eGFP sg3
bp
300
200
100
PCR product eGFP
BceAI (60) (72)
(120)
(201)
bp 0
50
100
150
200
236
sg3
C
Control
5dpf
Tg(tbx18:myr-Citrine)
V
D
KO Citrine
5dpf
Tg(tbx18:myr-Citrine)
V
E
Control
5dpf
Tg(tcf21:myr-eGFP)
V
F
KO eGFP
5dpf
Tg(tcf21:myr-eGFP)
V

Figure S5. Efficient transient gene knockout by CRISPR/Cas9 technology. Related to Figures 4-6.

(A) Restriction digest to test the efficiency of sgRNA4 (sg4)/Cas9-mediated nuclease activity targeting *Citrine*. The sgRNA binding sequence is indicated by a grey square. PCR products were digested using HpyAV, presence of undigested PCR product indicates the disruption of the restriction site. DNA from the embryo analysed in D is highlighted by a red box. (B) Restriction digest to test the efficiency of sgRNA3 (sg3)/Cas9-mediated nuclease activity targeting *eGFP*. PCR products were digested using BceAI. DNA from the embryo analysed in F is highlighted by a red box. (C) Projection of the heart in a 5dpf *TgBAC(tbx18:myr-Citrine)*^{ox185} larva injected with control sgRNA. (D) Projection of the heart in a 5dpf larva injected with sg4 targeting *Citrine* (KO *Citrine*), showing strongly disrupted fluorescence. (E) Projection of the heart in a 5dpf *TgBAC(tcf21:myr-eGFP)*^{ox183} larva injected with control sgRNA. (F) Projection of the heart in a 5dpf larva injected with sg3 targeting *eGFP* (KO *eGFP*). Scale bars: 50µm. V=ventricle.

Figure S6.

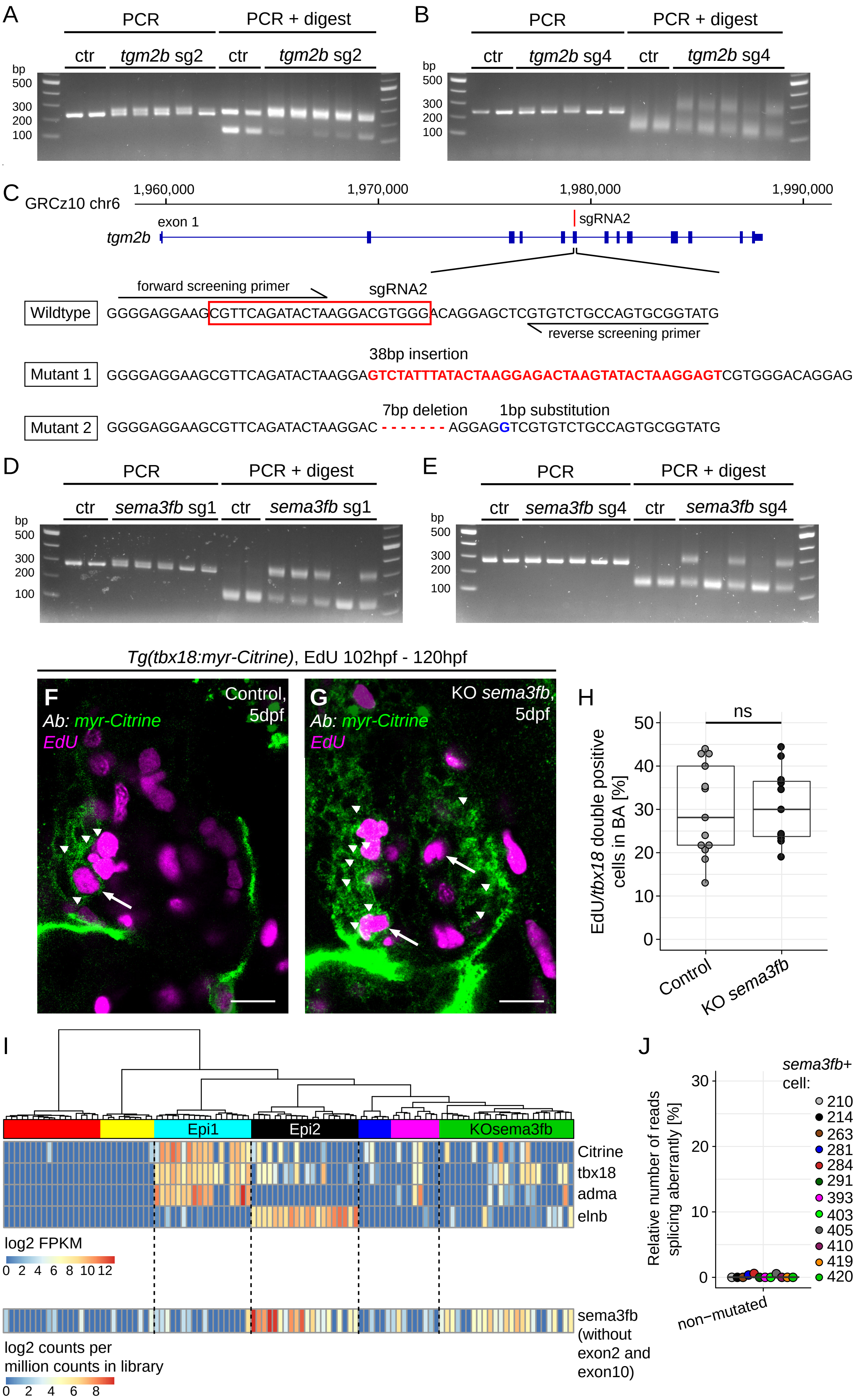

A
PCR
PCR + digest
ctr
tgm2b sg2
bp
500
300
200
100
B
tgm2b sg4
C
GRCz10 chr6
1,960,000
1,970,000
1,980,000
1,990,000
exon 1
sgRNA2
tgm2b
forward screening primer
Wildtype
GGGGAGGAAGCGTTCAGATACTAAGGACGTGGGACAGGAGCTCGTGTCTGCCAGTGCGGTATG
reverse screening primer
38bp insertion
Mutant 1
GGGGAGGAAGCGTTCAGATACTAAGGAGTCTATTTATACTAAGGAGACTAAGTATACTAAGGAGTCGTGGGACAGGAG
7bp deletion
1bp substitution
Mutant 2
GGGGAGGAAGCGTTCAGATACTAAGGAC - - - - - - - AGGAGGTCGTGTCTGCCAGTGCGGTATG
D
sema3fb sg1
E
sema3fb sg4
Tg(tbx18:myr-Citrine), EdU 102hpf - 120hpf
F
Control, 5dpf
Ab: myr-Citrine
EdU
G
KO sema3fb, 5dpf
H
ns
EdU/tbx18 double positive cells in BA [%]
0
10
20
30
40
50
Control
KO sema3fb
I
Epi1
Epi2
KOsema3fb
Citrine
tbx18
adma
elnb
log2 FPKM
0 2 4 6 8 10 12
sema3fb (without exon2 and exon10)
log2 counts per million counts in library
0 2 4 6 8
J
Relative number of reads splicing aberrantly [%]
sema3fb+ cell:
210
214
263
281
284
291
393
403
405
410
419
420
non−mutated

Figure S6. Testing the efficiency of sgRNA/Cas9-mediated nuclease activity targeting *tgm2b* and *sema3fb*. Related to Figures 4 and 5.

(A) Restriction digest to test the efficiency of sgRNA2 (sg2)/Cas9-mediated nuclease activity targeting *tgm2b*. PCR products were digested using BmgBI. (B) Restriction digest to test the efficiency of sgRNA4 (sg4)/Cas9-mediated nuclease activity targeting *tgm2b*. PCR products were digested using HpyCH4III. (C) Schematic indicating the genomic binding site of *tgm2b* sgRNA2 (red box) and the gene editing events identified in the two stable *tgm2b* mutant lines. (D) Restriction digest to test the efficiency of sgRNA1 (sg1)/Cas9-mediated nuclease activity targeting *sema3fb*. PCR products were digested using BsrI. (E) Restriction digest to test the efficiency of sgRNA4 (sg4)/Cas9-mediated nuclease activity targeting *sema3fb*. PCR products were digested using Fnu4HI. (F-H) EdU detection and antibody staining against myr-Citrine in 5dpf *TgBAC(tbx18:myr-Citrine)*^{ox185} larvae. EdU/myr-Citrine^{+} cells in the BA are highlighted by arrows, myr-Citrine^{+} cells by arrowheads. (F) The BA in a control larva. (G) The BA in a KO *sema3fb* larva. (H) Relative quantification of the number of *tbx18*:myr-Citrine^{+} cells in the BA that were EdU^{+}. (I) Heatmap showing marker gene expression (log2 FPKM) in *sema3fb*-mutated single cells. The expression of *sema3fb* is shown as log2 of *sema3fb* read counts per million library counts. *sema3fb* counts were computed excluding exon 2 and exon 10 to ensure that only *sema3fb* cDNA excluding genomic amplicons was quantified. (J) Levels of abnormally splicing reads in the *sema3fb* locus in non *sema3fb* knockout cells, relative to the total number of splicing *sema3fb* reads. Colours indicate cell identity. Scale bars in F,G: 10µm. Colour channels in microscopy images were adjusted separately for brightness/contrast. F,G are single optical sections. Data represented as median, first and third quartiles (box).

Significance was calculated using Welch’s t-test. Ab=antibody, ns=non-significant. H: n(control)=13, n(KO *sema3fb*)=11.

Figure S7.

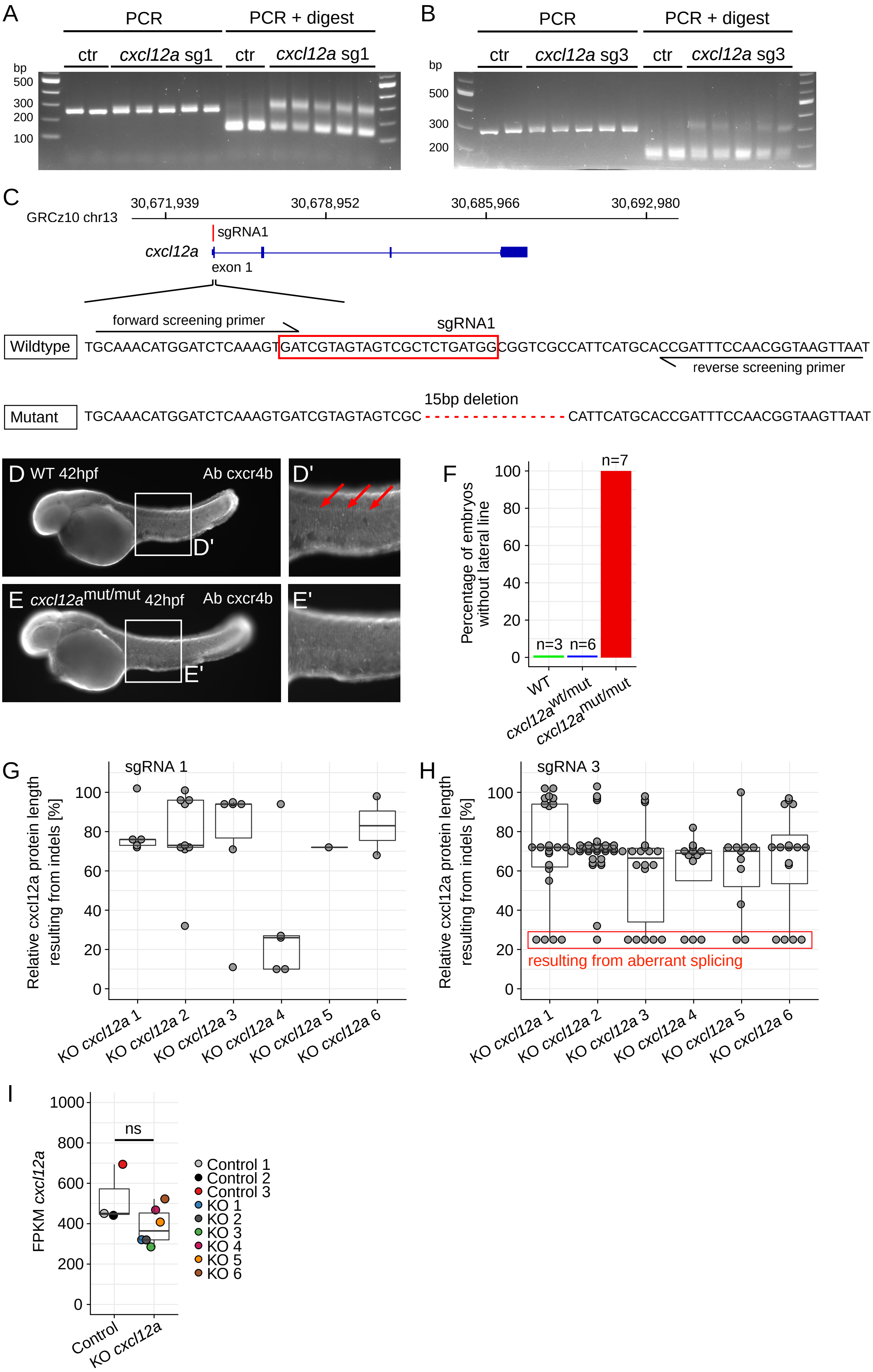
A
PCR
PCR + digest
ctr
cxcl12a sg1
bp
500
300
200
100
B
cxcl12a sg3
C
GRCz10 chr13
30,671,939
30,678,952
30,685,966
30,692,980
sgRNA1
cxcl12a
exon 1
forward screening primer
Wildtype
TGCAAACATGGATCTCAAAGTGATCGTAGTAGTCGCTCTGATGGCGGTCGCCATTCATGCACCGATTTCCAACGGTAAGTTAAT
reverse screening primer
15bp deletion
Mutant
TGCAAACATGGATCTCAAAGTGATCGTAGTAGTCGC - - - - - - - - - - - - - - - CATTCATGCACCGATTTCCAACGGTAAGTTAAT
D WT 42hpf
Ab cxcr4b
D'
E cxcl12a mut/mut 42hpf
E'
F
Percentage of embryos without lateral line
n=7
n=3
n=6
WT
cxcl12a wt/mut
cxcl12a mut/mut
G
sgRNA 1
Relative cxcl12a protein length resulting from indels [%]
KO cxcl12a 1
KO cxcl12a 2
KO cxcl12a 3
KO cxcl12a 4
KO cxcl12a 5
KO cxcl12a 6
H
sgRNA 3
resulting from aberrant splicing
I
FPKM cxcl12a
ns
Control
KO cxcl12a
Control 1
Control 2
Control 3
KO 1
KO 2
KO 3
KO 4
KO 5
KO 6

Figure S7. Testing the efficiency of sgRNA/Cas9-mediated nuclease activity targeting *cxcl12a*. Related to Figure 6.

(A) Restriction digest to test the efficiency of sgRNA1 (sg1)/Cas9-mediated nuclease activity targeting *cxcl12a*. PCR products were digested using BccI. (B) Restriction digest to test the efficiency of sgRNA3 (sg3)/Cas9-mediated nuclease activity targeting *cxcl12a*. PCR products were digested using BsmFI. (C) Schematic indicating the genomic binding site of *cxcl12a* sgRNA1 (red box) and the 15bp deletion identified in the stable *cxcl12a* mutant line. (D-F) Analysis of lateral line development via antibody staining against cxcr4b in wildtype (D) and homozygous *cxcl12a* mutant (E) embryos at 42hpf. (F) Quantification of the relative number of embryos analysed in which the lateral line was not present. (G,H) Quantification of inferred cxcl12a protein lengths due to editing at sgRNA1 (G) or sgRNA3 (H) target sites, relative to wildtype cxcl12a. Protein length generated by splicing from exon1 into exon 3 is indicated by a red box in H. (I) FPKM values of *cxcl12a* in control and *cxcl12a*-mutated (KO) samples. D,E are stereo microscope images. F: Data represented as mean, G-I: Data represented as median, first and third quartiles (box). Significance was calculated using Welch's t-test.

Primer name	Primer sequence
BAC Tcf21 H2B-Dendra2 fkf for	CATCTCCTCAAGAAGTCCTTTTCTCCACTCCACCCTTGTCTCCAGCCAACatgccagagccagcgaagtctg
BAC Tcf21 myr-tdTomato / myr-eGFP fkf for	CATCTCCTCAAGAAGTCCTTTTCTCCACTCCACCCTTGTCTCCAGCCAACatgggctgcatcaagagcaagc
BAC Tcf21 fkf rev	CCAAACAACATTAGATTAACCGAATCGGAAAACCAAAATGAATTTATGAAAACTCAATATTAATTCTGATTGCAAGAAGTGTCTCACtccaGAAGTAGTGAGGAGGCTTT
BAC Tbx18 myr-Citrine / myr-eGFP fkf for	TTCTGGTGAACTTCTCTTTCTCGGCCAATCTGTCTTCTCGGTCGGTAACCatgggctgcatcaagagcaagc
BAC Tbx18 fkf rev	TACTTACGGGATTCGTCGCTGGTGCAATCTATCTCGCAACTCCTGGTGCTtccaGAAGTAGTGAGGAGGCTTT
BAC Wt1b H2B-Dendra2 fkf for	GTGTTTTGCAACCCAGAAAATCCGTCTAAATGCTGACAGAGCCGTGCGGCCCGatgccagagccagcgaagtctg
BAC Wt1b Cre-2A-Cherry fkf for	GTGTTTTGCAACCCAGAAAATCCGTCTAAATGCTGACAGAGCCGTGCGGCCCGATGCCcAAgAAAAAGcGcAAaG
BAC Wt1b fkf rev	GACCACATTGAGAGAGATTTTGAGGCGAGATTGTAAGGACGGGATGGTTTTCTCACtccaGAAGTAGTGAGGAGGCTTT

Table S1: Primers used for BAC recombineering. Related to Figure 1.

Gene/sgRNA name	sgRNA forward primer	sgRNA reverse primer
tgm2b sg2	ttcgCGTTCAGATACTAAGGACGT	aaacACGTCCTTAGTATCTGAACG
tgm2b sg4	ttcgGCTGCAGGAACTATCACTGT	aaacACAGTGATAGTTCCTGCAGC
sema3fb sg1	ttcgGGACAGTCTATGGCCAGTCC	aaacGGACTGGCCATAGACTGTCC
sema3fb sg4	ttcgTGACGGTGGTCACTGCTGCT	aaacAGCAGCAGTGACCACCGTCA
cxcl12a sg1	ttcgGATCGTAGTAGTCGCTCTGA	aaacTCAGAGCGACTACTACGATC
cxcl12a sg3	ttcgGCTCGCGAATGCTTCTCTGT	aaacACAGAGAAGCATTCGCGAGC
Citrine sg4	ttcgCTCGTGACCACCTTCGGCTA	aaacTAGCCGAAGGTGGTCACGAG
eGFP sg3	ttcgGCACTGCACGCCGTAGGTCA	aaacTGACCTACGGCGTGCAGTGC
mCherry sg1	ttcgCGGCCACGAGTTCGAGATCG	aaacCGATCTCGAACTCGTGGCCG
mCherry sg2	ttcgTCTGGGTGCCCTCGTAGGGG	aaacCCCCTACGAGGGCACCCAGA
AmCyan sg1	ttcgGAATGCAAGGGGCCCACCGT	aaacACGGTGGGCCCCTTGCATTC
AmCyan sg2	ttcgCTATCTACAGTGTTCATGTA	aaacTACATGAACACTGTAGATAG

Table S2: Primers used for Golden Gate cloning of sgRNA sequences into the U6a promoter Ac/Ds mini-vector. Related to Figures 4-6. sgRNA sequences (without PAM) are underlined.

**Gene/ sgRNA name**	**PCR forward primer**	**PCR reverse primer**	**Product size (bp)**
tgm2b sg2	CAGTAATGACCAGGATTCAGGG	gtgcatgtagcccATTAAGGAT	221
tgm2b sg4	ctgtgcaaaacatatgctggat	TGTGCACACCTTCACTTTTCTC	228
sema3fb sg1	ATTGCCCCACAAAATAACATTC	CAATCCTTAAAAGGAACGCAAG	273
sema3fb sg4	TTCTCACAGCTAACTTTTCCCG	TGTTGGAGATGGAAACAGACAC	259
cxcl12a sg1	AGCCTGTTCGCAAATTTAACTC	TCACCATAAAGTGTGCCAAAAC	245
cxcl12a sg3	TCATTATTCCATCCAACGTTCA	CCATTCAGTGTTTGGAGATTGA	284
Citrine, *eGFP*	GAGGAGCTGTTCACCGGG	AAGTCGTGCTGCTTCATGTG	236
mCherry sg1, sg2	AGGGCGAGGAGGATAACATG	TCAAGTAGTCGGGGATGTCG	254
AmCyan sg1, sg2	CCATATGGATGGCTGTGTCA	CATTCCGTCAGGAAATGCTT	231

Table S3: Primers used to amplify genomic regions containing sgRNA/Cas9 target sites. Related to Figures 4-6.

Primer name	Primer sequence	PCR product size [bp]
sema3fb mRNA for	ATTTGAGGCGAGCGAATGAGT	1669
sema3fb mRNA rev	ACCTCGTCTGGGTAGTCCTT	-
sema3fb-ISPCR cDNA for	aagcagtggtatcaacgcagagtATTTGAGGCGAGCGAATGAGT	1715
sema3fb-ISPCR cDNA rev	aagcagtggtatcaacgcagagtACCTCGTCTGGGTAGTCCTT	-
sema3fb gDNA sg1 for	CAGGGGAATTGCATGGTCCT	3683
sema3fb gDNA sg1 rev	AGTGGGGTTGGATGTCATGG	-
sema3fb gDNA sg4 for	ACTGAGCTGAAACAACCTCACTT	3104
sema3fb gDNA sg4 rev	ATTCTCAGTCTGTACGCTTGGCT	-
cxcl12a-ISPCR cDNA rev	aagcagtggtatcaacgcagagtGTGTGTTGTGACGCTGAGTTC	1395

Table S4. Primers used to amplify *sema3fb* cDNA and genomic regions in *sema3fb*-mutated single cells. Related to Figure 5.
