# Supplementary material for: Functional Heterogeneity within the Developing Zebrafish Epicardium
Source: Dev Cell. 2020 Mar 9;52(5):574–590.e6. doi: 10.1016/j.devcel.2020.01.023 (PMC7063573; doi:10.1016/j.devcel.2020.01.023)
Supplement: Document S2. Article plus Supplemental Information [file mmc2.pdf]

Developmental Cell

Article

Functional Heterogeneity within the Developing Zebrafish Epicardium

Graphical Abstract

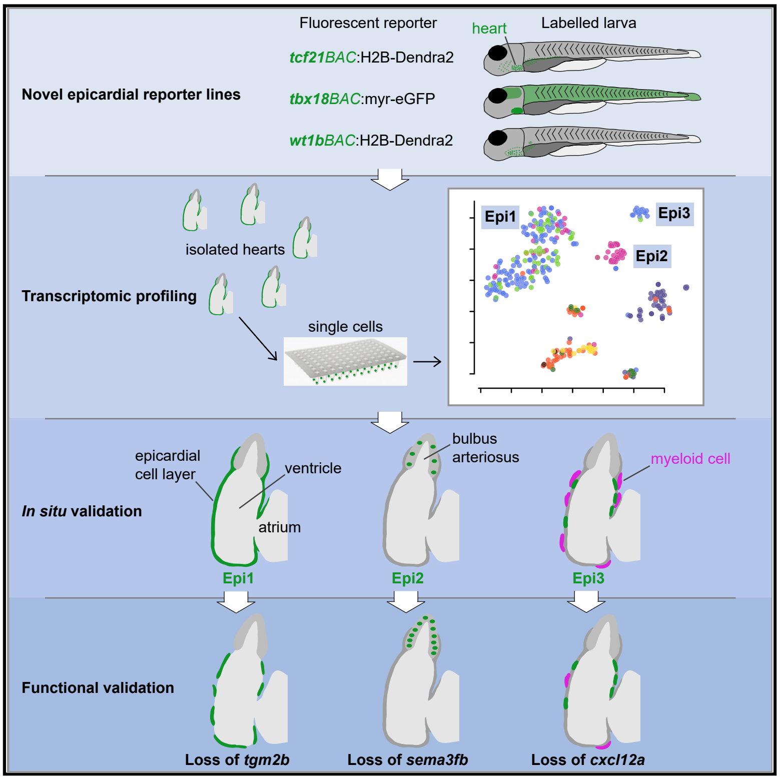

Highlights

- scRNA-seq uncovered 3 developmental epicardial subpopulations (Epi1-3) in the zebrafish
- Epi1-specific gene, *tgm2b*, regulates the cell numbers in the main epicardial sheet
- Epi2-specific gene, *sema3fb*, restricts the number of *tbx18*$^+$ cells in the cardiac outflow tract
- Epi3-specific gene, *cxcl12a*, guides *ptprc/CD45*$^+$ myeloid cells to the developing heart

Authors

Michael Weinberger, Filipa C. Simões, Roger Patient, Tatjana Sauka-Spengler, Paul R. Riley

Correspondence

tatjana.sauka-spengler@imm.ox.ac.uk (T.S.-S.),
paul.riley@dpag.ox.ac.uk (P.R.R.)

In Brief

Weinberger et al. identify three cell subpopulations with unique gene expression signatures and patterns in the developing zebrafish epicardium. These epicardial subpopulations fulfill distinct functions in the formation of the epicardial cell layer, the development of the cardiac outflow tract, and the guidance of myeloid cells into the heart.

Weinberger et al., 2020, Developmental Cell *52*, 574–590
March 9, 2020 © 2020 The Authors. Published by Elsevier Inc.
https://doi.org/10.1016/j.devcel.2020.01.023

CellPress

Developmental Cell
Article

Functional Heterogeneity within the Developing Zebrafish Epicardium

Michael Weinberger,[1,2,3] Filipa C. Simões,[1,2,3] Roger Patient,[2] Tatjana Sauka-Spengler,[2,4,*] and Paul R. Riley[1,4,5,*]
[1]Department of Physiology, Anatomy and Genetics, University of Oxford, Oxford, Oxfordshire OX1 3PT, UK
[2]MRC Weatherall Institute of Molecular Medicine, Radcliffe Department of Medicine, University of Oxford, Oxford, Oxfordshire OX3 9DS, UK
[3]These authors contributed equally
[4]Senior author
[5]Lead Contact
*Correspondence: tatjana.sauka-spengler@imm.ox.ac.uk (T.S.-S.), paul.riley@dpag.ox.ac.uk (P.R.R.)
https://doi.org/10.1016/j.devcel.2020.01.023

SUMMARY

The epicardium is essential during cardiac development, homeostasis, and repair, and yet fundamental insights into its underlying cell biology, notably epicardium formation, lineage heterogeneity, and functional cross-talk with other cell types in the heart, are currently lacking. In this study, we investigated epicardial heterogeneity and the functional diversity of discrete epicardial subpopulations in the developing zebrafish heart. Single-cell RNA sequencing uncovered three epicardial subpopulations with specific genetic programs and distinctive spatial distribution. Perturbation of unique gene signatures uncovered specific functions associated with each subpopulation and established epicardial roles in cell adhesion, migration, and chemotaxis as a mechanism for recruitment of leukocytes into the heart. Understanding which mechanisms epicardial cells employ to establish a functional epicardium and how they communicate with other cardiovascular cell types during development will bring us closer to repairing cellular relationships that are disrupted during cardiovascular disease.

INTRODUCTION

During embryonic development, numerous cardiovascular cell types interact to build and maintain a functional heart. The epicardium is a mesothelial cell sheet that covers the heart's outer surface and, together with the myocardium and the endocardium, forms the wall of the heart (reviewed in Simões and Riley [2018]). The epicardium derives from a transient extracardiac structure called the proepicardium (PEO) (Männer et al., 2001; Peralta et al., 2013), which emerges at around 55 h post fertilization (hpf) in the zebrafish embryo. Between 55 hpf and 72 hpf, proepicardial cells translocate to the ventricle via pericardial fluid movements, where they form a continuous cell layer (Peralta et al., 2013). Subsequently, a subset of epicardial cells undergoes an epithelial-to-mesenchymal transition, giving rise to epicardium-derived cells (EPDCs) (von Gise and Pu, 2012). These delaminating cells invade the subepicardial compartment and colonize the underlying myocardium to nurture the further growth of the developing heart muscle and coronary vessels by acting as an essential source of mitogens (Pérez-Pomares and de La Pompa, 2011).

In addition to providing signals, EPDCs can directly give rise to many of the cell types that form the developing heart. Studies have reported an epicardial contribution to adipose tissue (Chau et al., 2014; Liu et al., 2014; Yamaguchi et al., 2015), vascular smooth muscle cells, necessary for vascular support and proper coronary formation, and cardiac fibroblasts in chick (Dettman et al., 1998; Männer, 1999; Mikawa and Gourdie, 1996; Pérez-Pomares et al., 1997), mouse (Acharya et al., 2012; Swonger et al., 2016; Wessels et al., 2012; Wu et al., 2013; Zhou et al., 2010), and zebrafish (Kikuchi et al., 2011). A much less consensual view of EPDC fate exists concerning their putative differentiation into endothelial cells (Dettman et al., 1998; Mikawa and Fischman, 1992; Pérez-Pomares et al., 2002, 1998; Zhou et al., 2008) and cardiomyocytes (Cai et al., 2008; del Monte et al., 2011; Guadix et al., 2006; Ruiz-Villalba et al., 2013; Zhou et al., 2008). These findings have relied heavily on tissue transplantation and Cre-based fate mapping analyses, which have limitations and may confound interpretation of results because of issues with activation of the Cre drivers in lineage derivatives and mosaic or ectopic expression of reporters (Davis et al., 2012).

Although there has been significant progress understanding the biology of the epicardium, it is still not clear whether premigratory EPDCs are a homogeneous source of multipotent progenitors or become specified as epicardial subpopulations already within the epicardium proper. In the developing mouse and chick heart, the epicardial expression of *Tcf21*, *Wt1*, and *Tbx18* is restricted to subsets of cells (Braitsch et al., 2012), establishing a precedent for cellular heterogeneity in the epicardium itself. This is supported by a recent study showing conserved heterogeneity within epicardium derived from human pluripotent stem cells (Gambardella et al., 2019). Epicardial heterogeneity might be rooted in the fact that multiple tissues contribute to this structure. Although most zebrafish ventricular epicardial cells originate from the PEO, the epicardium covering the bulbus arteriosus (BA) was found to originate from the pericardial sac (Peralta et al., 2014; Pérez-Pomares et al., 2003). Additionally, the PEO itself was shown to be heterogeneous (Plavicki et al., 2014). A subset of murine proepicardial

This is an open access article under the CC BY license (http://creativecommons.org/licenses/by/4.0/).

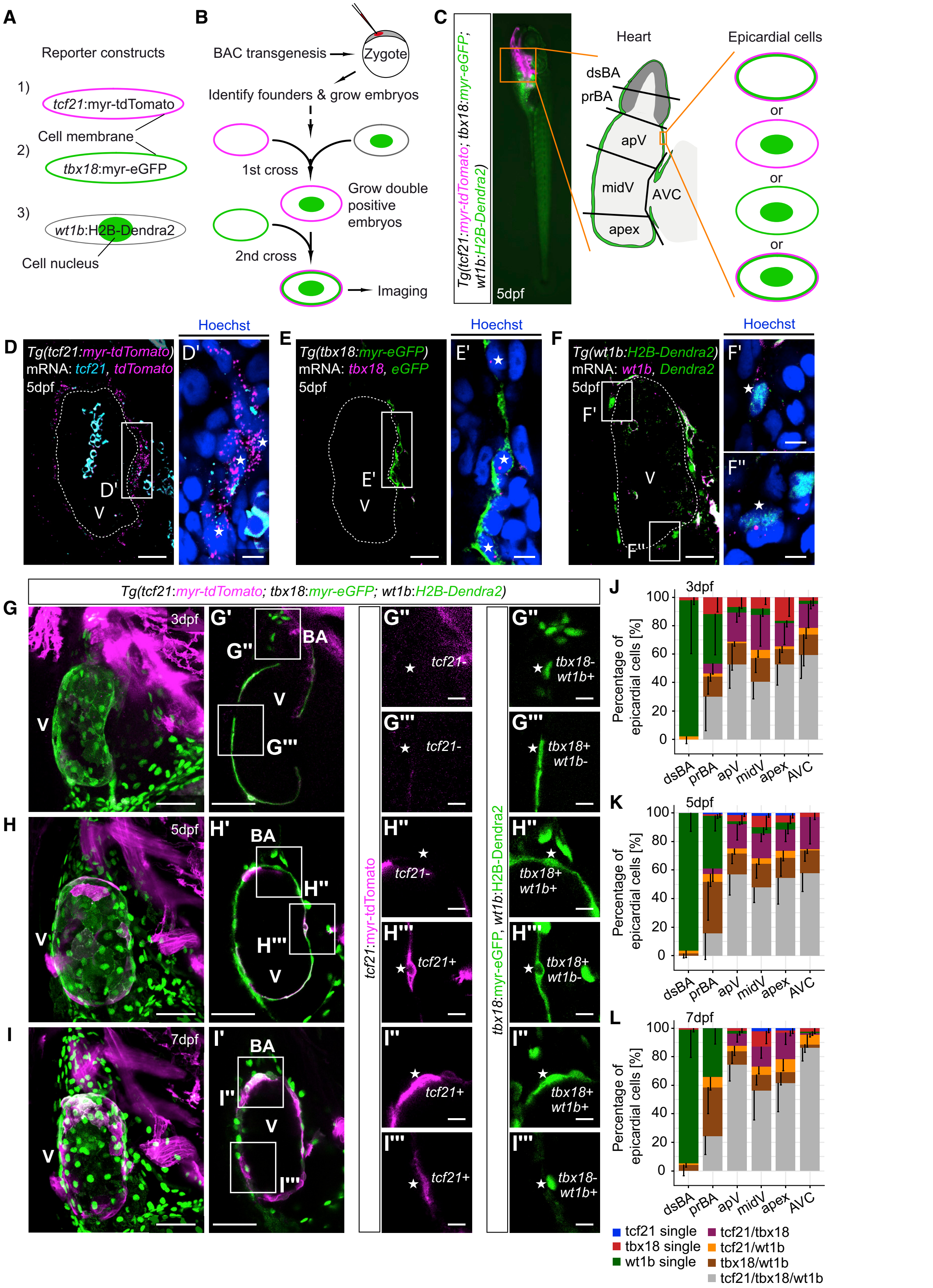

(legend on next page)

cells that expresses the transcription factor Scleraxis (Scx) and the chemokine Semaphorin 3D (Sema3D) gives rise to the endocardium and coronary endothelium (Katz et al., 2012). Most of these cells do not express Wt1 or Tbx18.

However, previous insights into epicardial heterogeneity have remained limited and restricted to a small number of epicardial markers. To gain unbiased insight into epicardial cell heterogeneity, we characterized the developmental transcriptome of the zebrafish epicardium at a single-cell level, combining confocal microscopy of newly generated epicardial reporter lines and single-cell transcriptomics. We identified and functionally characterized three transcriptionally distinct epicardial cell subpopulations, only one of which (Epi1) contained cells co-expressing the bona fide epicardial signature genes *tcf21*, *tbx18*, and *wt1b* (Kikuchi et al., 2011; Serluca, 2008). Functional perturbation identified *tgm2b*, a transglutaminase gene highly enriched in Epi1, as necessary for the proper development of the epicardial cell sheet. The second subpopulation (Epi2), enriched in *tbx18* and smooth muscle markers such as *acta2* and *mylka*, was spatially localized outside the main epicardial layer, specifically in the smooth muscle of the BA. Loss of the chemokine semaphorin *3fb* (*sema3fb*), highly enriched in Epi2, increased the number of *tbx18*$^+$ cells in the BA, revealing that *sema3fb* controlled the spatiotemporal access of epicardial cells to the outflow tract. The third subpopulation (Epi3) was highly enriched for cell guidance cues such as *cxcl12a*. Loss of this chemokine decreased the number of *ptprc/CD45*$^+$ leukocytes on the epicardial surface, unraveling the importance of Epi3 in establishing intercellular communications responsible for the recruitment and/or retention of *ptprc/CD45*$^+$ hematopoietic cells within the developing heart.

Building on the understanding of the genetic control of the developing epicardium, our study defines the molecular signatures and functional roles of specific epicardial cell subpopulations, thus providing insights into the specific programs within which the epicardium functions during cardiac development.

RESULTS

Expression of *tcf21*, *tbx18*, and *wt1b* Is Heterogeneous in the Developing Zebrafish Epicardium

Expression of *Tcf21, Tbx18*, and *Wt1* is restricted to subsets of epicardial cells in the developing mouse and chick heart (Braitsch et al., 2012). Similar heterogeneity is present in the zebrafish epicardium (González-Rosa et al., 2012; Kikuchi et al., 2011). However, the concurrent expression of all three epicardial genes has not been analyzed. Thus, we generated the zebrafish triple-reporter line *TgBAC(tcf21:myr-tdTomato;tbx18:myr-eGFP;wt1b:H2B-Dendra2)*^{ox187} (Figures 1A and 1B). Bacterial artificial chromosomes (BACs) contain large genomic fragments and recapitulate endogenous gene expression patterns more faithfully than promoter- or proximal enhancer-based transgenic lines (Bussmann and Schulte-Merker, 2011). In this line, membrane-tethered tdTomato and eGFP label *tcf21*$^+$ and *tbx18*$^+$ cells, respectively. Dendra2, localizing to the nucleus, identifies *wt1b*$^+$ cells. The resulting combination of fluorescent signals enabled us to discriminate between epicardial cells that expressed combinations of *tcf21*, *tbx18*, and *wt1b* (Figure 1C). Whole-mount *in situ* hybridization chain reaction (HCR) (Choi et al., 2010, 2018) validated the newly generated BAC reporter lines (Figures 1D–1F, 1D′–1F′, and 1F″). Confocal imaging of triple-reporter larvae at 3 days post fertilization (dpf) (Figures 1G and 1G′), 5 dpf (Figures 1H, 1H′, and S1A–S1C), and 7 dpf (Figures 1I and 1I′) revealed that many epicardial cells did not express *tcf21*, *tbx18*, and *wt1b* simultaneously, but different subsets of the three markers (Figures 1G″–1I″ and 1G‴–1I‴). Interestingly, the distribution of these subsets differed across distinct morphological regions of the heart (Figures 1C and 1J–1L). For example, *wt1b*-only positive cells were mostly found on the BA, whereas *tcf21/tbx18/wt1b* triple-positive cells were mostly present on the ventricle. Furthermore, the relative number of triple-positive epicardial cells increased over time in all ventricular regions. However, in none of the cardiac regions did triple-positive cells become the only present epicardial subset. To validate these findings, we crossed the pre-existing reporter lines *TgBAC(tcf21:DsRed2)*^{pd37} and *TgBAC(tbx18:DsRed2)*^{pd22} (Kikuchi et al., 2011) to *Tg(wt1b:eGFP)*^{li1} (Perner et al., 2007) and observed clear heterogeneity in the *tcf21/wt1b* and *tbx18/wt1b* double-fluorescent settings (Figures S1D–S1K). We also observed epicardial heterogeneity at the endogenous gene expression level (Figure S1L). At 5 dpf, we detected nuclei adjacent to *myl7*$^+$ myocardium that featured endogenous *tcf21*, *tbx18*, and *wt1b* transcripts (Figure 1L″, asterisk). However, we also detected *tcf21*$^+$ and *wt1b*$^+$ areas that lacked *tbx18* expression (Figure S1L‴, asterisk).

Overall, both newly generated BAC transgenic reporter lines and co-expression of endogenous transcripts demonstrate

Figure 1. Heterogeneous Expression of *tcf21*, *tbx18*, and *wt1b* in the Developing Zebrafish Epicardium

(A) Fluorescence of *tcf21*:myr-tdTomato (magenta membrane), *tbx18*:myr-eGFP (green membrane), and *wt1b*:H2B-Dendra2 (green nucleus).

(B) Workflow to establish *TgBAC(tcf21:myr-tdTomato;tbx18:myr-eGFP;wt1b:H2B-Dendra2)*^{ox187}. (C) Schematic of epicardial fluorescence patterns and cardiac regions analyzed. dsBA, distal bulbus arteriosus; prBA, proximal bulbus arteriosus; apV, arterial pole of the ventricle; and AVC, atrioventricular canal.

(D–F) Single optical sections of mRNA stainings of (D) *tcf21* (cyan) and *tcf21*:myr-tdTomato (magenta), (E) *tbx18* (magenta) and *tbx18*:myr-eGFP (green), and (F) *wt1b* (magenta) and *wt1b*:H2B-Dendra2 (green). (D′–F′) Cell nuclei (asterisks) in the epicardial region surrounded by endogenous mRNA and fluorophore mRNA/protein.

(G–I) Projections of the heart in triple reporter larvae at 3 dpf (G), 5 dpf (H), and 7 dpf (I).

(G′ and H′) Single optical sections from (G) and (H).

(I′) Short projection from (I).

(G″–I″ and G‴–I‴) Representative epicardial fluorescence patterns.

(J–L) Relative quantification of epicardial fluorescence patterns across the regions indicated in (C) at 3 dpf (J), 5 dpf (K), and 7 dpf (L).

Scale bars: 20 μm in (D)–(F), 5 μm in (D′)–(F′), 50 μm in (G)–(I) and (G′)–(I′), and 10 μm in (G″)–(I″) and (G‴)–(I‴). Color channels were adjusted separately for brightness and contrast. Data in (J)–(L) are represented as mean minus standard deviation. Number of embryos analyzed: 3 dpf n = 6, 5 dpf n = 10, and 7 dpf n = 6. V, ventricle; BA, bulbus arteriosus.

See also Figure S1.

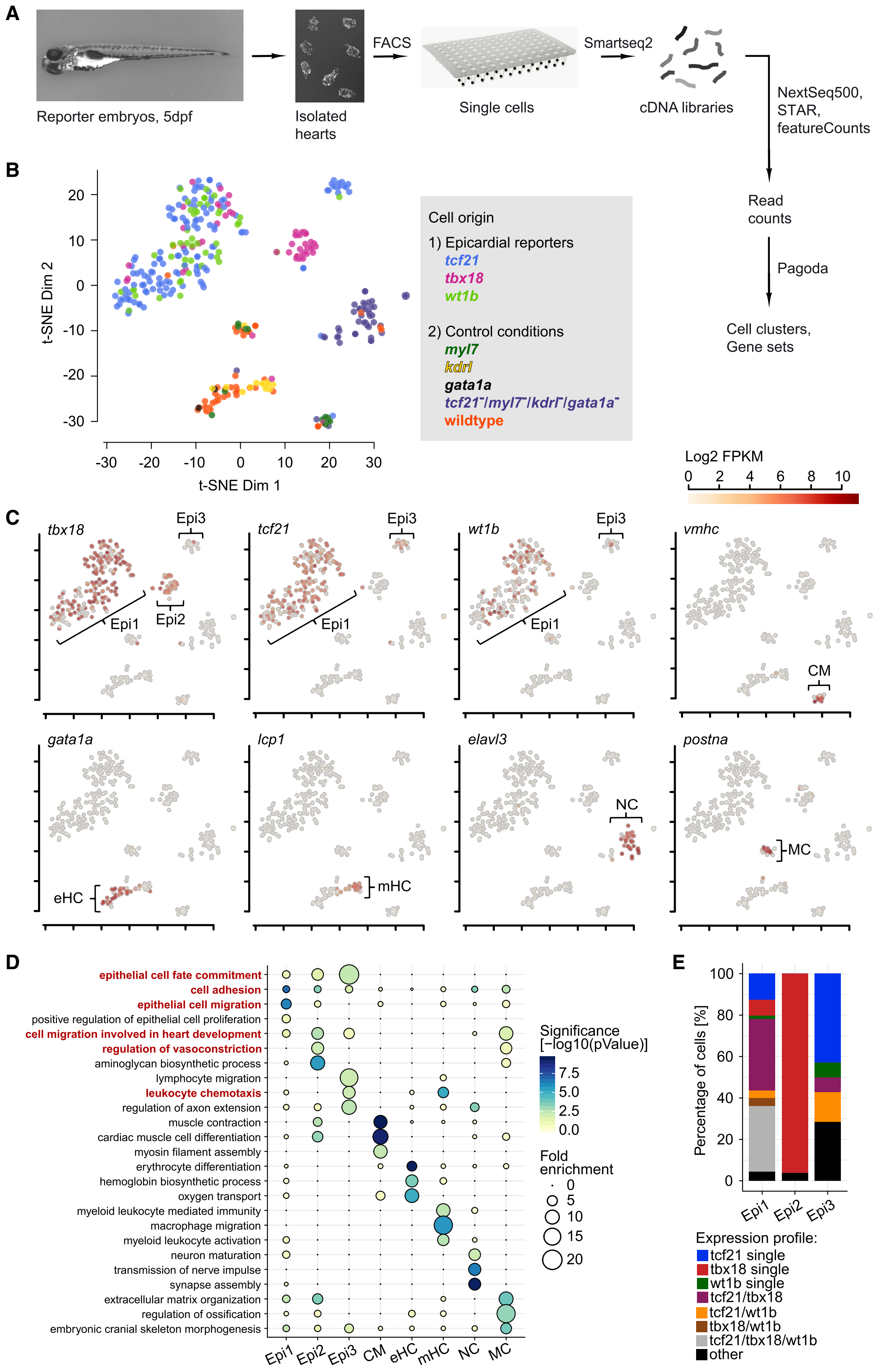

(legend on next page)

that the expression of *tcf21*, *tbx18*, and *wt1b* in the developing zebrafish epicardium is heterogeneous.

Single-Cell Transcriptomic Profiling Identifies Distinct Cell Populations within the Developing Zebrafish Epicardium

To further investigate the observed cellular heterogeneity, we performed single-cell RNA sequencing (scRNA-seq) using Smart-seq2 approach (Picelli et al., 2013) and a NextSeq500 platform (Illumina) to obtain 75-bp paired-end sequencing reads of the generated libraries (see Figures S2A–S2F for quality control data). Because only a small fraction of cells in the heart is epicardial, we fluorescence-activated cell sorting (FACS)-purified cells from hearts extracted from *TgBAC(tcf21:H2B-Dendra2)^{ox182}*, *TgBAC(tbx18:myr-eGFP)^{ox184}*, and *TgBAC(wt1b:H2B-Dendra2)^{ox186}* transgenic embryos (Figure 2A; see Figures S2G–S2K for FACS gating plots). To enhance cell clustering in our dataset, we also isolated cells from non-epicardial cardiac tissues such as myocardium (*Tg(myl7:eGFP)^{f1}*) (Huang et al., 2003), endocardium (*Tg(kdrl:GFP)^{s843}*) (Beis et al., 2005), and blood (*Tg(gata1a:DsRed)*) (Traver et al., 2003), as well as cells from wild-type (WT) hearts. To enrich for *tcf21*$^-$ epicardial cells, we purified non-fluorescent cells from *tcf21*:DsRed2; *myl7*:eGFP; *kdrl*:GFP; *gata1a*:DsRed quadruple transgenic reporter hearts. Following sequencing, we used the Pagoda pipeline (Fan et al., 2016) and dimensionality reduction to uncover three distinct epicardial cell populations (Figure 2B). These populations, termed Epi1, Epi2, and Epi3, were predominantly formed by cells isolated from the epicardial reporter hearts, and cells within these subpopulations expressed *tcf21*, *tbx18*, and *wt1b* in a differential manner (Figure 2C). *tbx18* was mostly expressed in cells within Epi1 and Epi2 and *tcf21* within Epi1 and Epi3, whereas *wt1b* was confined to subsets of cells within Epi1 and, to a lesser extent, Epi3. We further identified five distinct non-epicardial cell clusters expressing known markers of other cardiac cell types, namely cardiomyocytes (Figure 2C, CMs; *vmhc* [Yelon et al., 1999]), erythroid hematopoietic cells (Figure 2C, eHCs; *gata1a* [Lyons et al., 2002]), myeloid or leukocyte hematopoietic cells (Figure 2C, mHCs; *lcp1* [Kell et al., 2018]), neural cells (Figure 2C, NCs; *elavl3* [Park et al., 2000]) and mesenchymal cells (Figure 2C, MCs; *postna* [Snider et al., 2009]). This classification was supported by the enrichment of additional genes (Figure S3) and by the over-representation of gene ontology (GO) terms within the individual cell populations (Figure 2D). For example, cells in Epi1, Epi2, and Epi3 expressed many genes associated with epithelial cell fate commitment. Cells in Epi1 were enriched for genes associated with cell adhesion (Fisher's exact test; p = 1.3E−07) and epithelial migration (p = 6.1E−07), fitting the epicardial capacity to migrate as an epithelial cell sheet (Wang et al., 2015). Cells in Epi2 expressed genes involved in vasoconstriction (p = 0.0227) and cell migration involved in heart development (p = 0.0021), suggesting they might fulfill a function outside the epicardial cell layer. Cells in Epi3 were enriched for genes associated with white blood cell migration (p = 0.0065) and axon extension (p = 0.0027), suggesting they might guide non-epicardial cells into the developing heart. To compare the reporter-based results described in Figure 1 with the endogenous transcriptomic data, we quantified the expression of *tcf21*, *tbx18*, and *wt1b* in Epi1, Epi2, and Epi3 and found that only Epi1 contained cells that co-expressed all three markers (Figure 2E). Most cells in Epi2 exclusively expressed *tbx18*, and many cells in Epi3 exclusively expressed *tcf21*. Thus, the 5 dpf zebrafish heart contains three transcriptionally distinct epicardial cell populations that differentially express *tcf21*, *tbx18*, and *wt1b*.

Epi1, Epi2, and Epi3 Are Distinct in Their Transcriptomic Profile and Spatial Distribution within the Developing Heart

Next, we interrogated the transcriptomic profiles of Epi1, Epi2, and Epi3. Single-cell gene enrichment analysis using scde (Kharchenko et al., 2014) showed that the retinoic acid synthesizing enzyme *retinaldehyde dehydrogenase* (*aldh1a2*), a known epicardial marker (Kikuchi et al., 2011; Lepilina et al., 2006), labeled Epi1 (Figure 3A). Epi1 cells also expressed genes without a previously ascribed epicardial function, such as the signaling peptide *adrenomedullin a* (*adma*), and the adhesion molecules *junctional adhesion molecule 2b* (*jam2b*) and *podocalyxin like* (*podxl*), suggesting that Epi1 might form the main epicardial cell sheet enveloping the heart. Using multiplexed hybridization chain reaction (HCR) *in situ* staining, we observed that both *adma* and *jam2b* mRNAs were present within the epicardial cell layer at 5 dpf, co-localizing with *tcf21* transcripts (Figures 3B–3B″).

In addition to *tbx18*, Epi2 cells expressed smooth muscle cell markers such as *smooth muscle actin* (*acta2*) and *myosin light chain kinase* (*mylka*), as well as extracellular matrix components such as *lysyl oxidase a* (*loxa*) and *elastin b* (*elnb*) (Figure 3A). *elnb* was previously shown to be specifically expressed in the BA of the zebrafish heart (Moriyama et al., 2016). Accordingly, we found the BA to be the only tissue in the 5 dpf zebrafish heart that expressed *elnb* mRNA (Figures 3C–3C″), as well as Mylka protein (Figures 3D–3D″). Furthermore, *tbx18* mRNA co-localized with *elnb* mRNA in the BA, and multiple Mylka$^+$ cells were co-labeled by *tbx18*-driven myr-Citrine. These data reveal that Epi2 represents *tbx18*$^+$ cells exclusively located in the smooth muscle layer of the BA.

Figure 2. Single-Cell RNA Sequencing Reveals Distinct Epicardial Cell Clusters in the Developing Zebrafish Heart

(A) Overview of the scRNA-seq workflow.

(B) t-distributed stochastic neighbor embedding (t-SNE) clustering of single-cell samples. Colors indicate the reporter or WT line cells were isolated from.

(C) Identification of single-cell clusters based on marker gene expression. Three clusters labeled by *tcf21*, *tbx18*, and *wt1b* are designated Epi1-3. Color key indicates range of log_2 transformed fragments per kilobase of transcript per million reads mapped (FPKM) expression values.

(D) GO term over-representation in the clusters (columns). Bubble size depicts fold enrichment and color scale depicts the statistical significance, with p-values calculated using Fisher's exact test and Bonferroni correction for multiple hypothesis testing. GO terms important for further analysis are highlighted in red.

(E) Relative quantification of the combinations of *tcf21*, *tbx18*, and *wt1b* expression in epi1–3 cells. CM, cardiomyocyte; eHC, erythroid hematopoietic cell; mHC, myeloid hematopoietic cell; NC, neural cell; and MC, mesenchymal cell.

See also Figures S2 and S3.

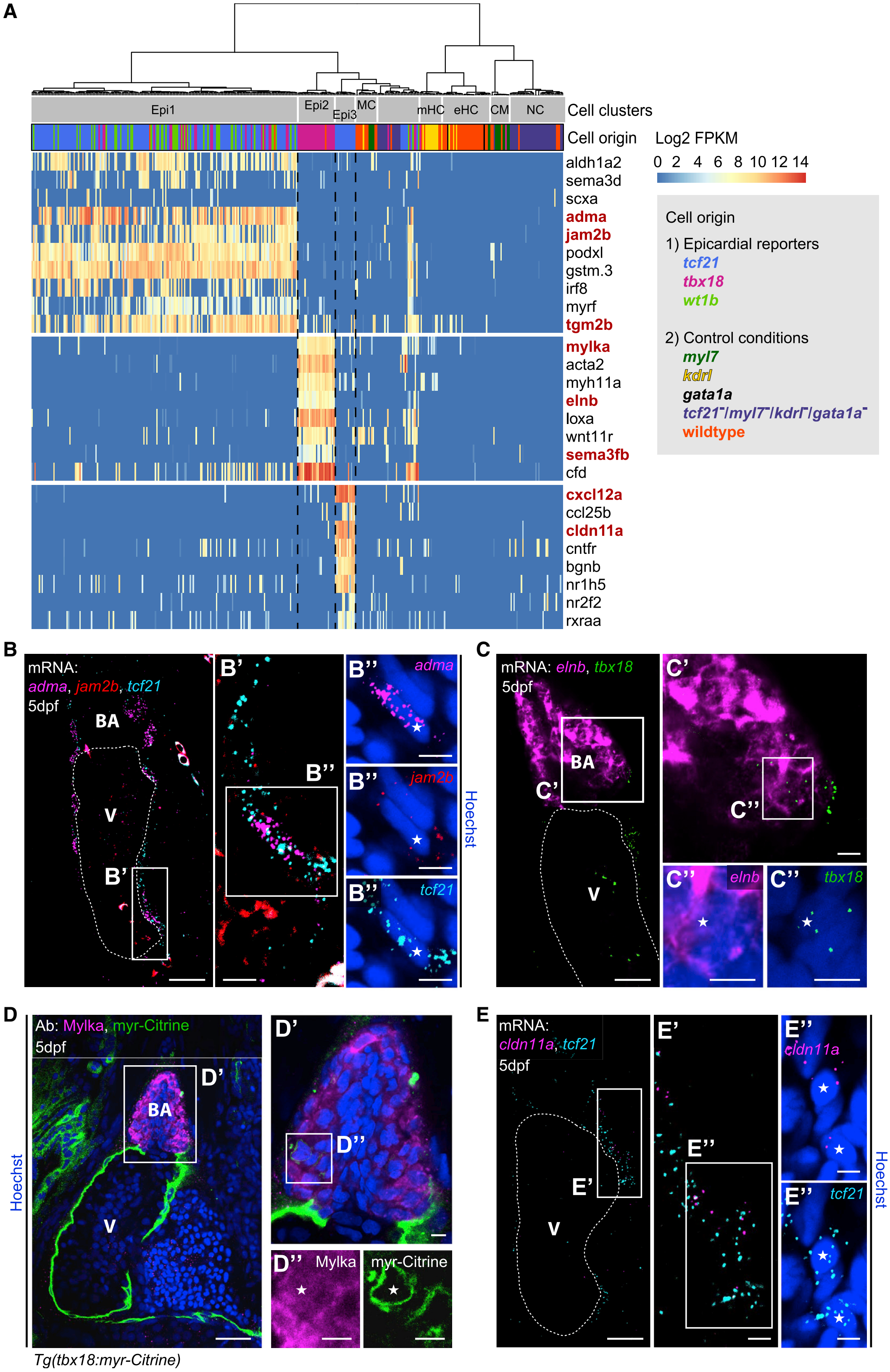

(legend on next page)

RNA velocity analysis (La Manno et al., 2018) indicated that Epi2 cells might originate from cells resembling an Epi1 subpopulation (Figure S4A). Furthermore, pseudotime trajectories (Trapnell et al., 2014) separated Epi1 from Epi2, but also revealed a subset of cells in Epi1 positioned proximally to Epi2 (Figure S4B), suggesting that transcriptional transition from Epi1 to Epi2 could drive cellular transdifferentiation. To establish a lineage relationship between Epi2 and the epicardium beyond *tbx18* expression, we performed a tracing experiment using a *tcf21*-driven Cre *TgBAC(cryaa:EGFP,tcf21:Cre-ERT2)*^{pd42} (Kikuchi et al., 2011) crossed to the ubiquitous labeling line *Tg(ubi:Switch)* (Mosimann et al., 2011). We incubated embryos with 4-hydroxytamoxifen (4-OHT) from either 10 hpf or 43 hpf, shortly before the PEO starts to form (Peralta et al., 2013; Serluca, 2008). Similar to previous findings in the adult zebrafish BA (Kikuchi et al., 2011), we observed *tcf21*-derived cells in the smooth muscle layer of the larval BA, independently of when the 4-OHT treatment was started (Figures S4C–S4E, $p = 0.1614$). This finding strongly supports an epicardial origin of Epi2 cells rather than an upregulation of *tbx18* mRNA in the BA. However, lineage tracing with a newly generated *TgBAC(wt1b:Cre-2A-mCherry)*^{ox142} line did not identify *wt1b*-derived cells in the BA (Figures S4F and S4G), indicating that cells in Epi2 never express *wt1b*. Endogenous *wt1b* co-localized with *mCherry* transcripts (Figure S4H), which, together with co-localization with the *TgBAC(wt1b:H2B-Dendra2)*^{ox186} reporter expression (Figure S4I), validated the activity of the *TgBAC(wt1b:Cre-2A-mCherry)*^{ox142} line.

Cells in Epi3 expressed *claudin 11a* (*cldn11a*), coding for an essential tight junction component (Gow et al., 1999) (Figure 3A). We observed co-localization of *cldn11a* and *tcf21* transcripts by HCR (Figures 3E–3E″), mostly in the epicardial region between the BA and the atrioventricular boundary. The spatial restriction of *cldn11a* expression was consistent with the small number of cells forming Epi3 (Figure 2C). Additionally, cells in Epi3 expressed the retinoic acid-responsive factors *nr1h5* and *rxraa*, known to interact in mammalian cells (Otte et al., 2003), and the chemokine *cxcl12a* (Figure 3A). Therefore, Epi3 might be responsive to retinoic acid and involved in the guidance of non-epicardial cells.

The Epi1-Enriched Gene *Transglutaminase 2b* Is Essential for Maintaining the Integrity of the Epicardial Cell Layer

To address whether epicardial heterogeneity underlies distinct cell fates and/or function, we used CRISPR-Cas9-mediated gene knockout. As single guide RNAs (sgRNAs) are rapidly degraded when not incorporated into Cas9 protein (Burger et al., 2016; Hendel et al., 2015), we used the Activator (Ac)-Dissociation (Ds) transposable element system (Emelyanov et al., 2006; McCLINTOCK, 1950) to stably express sgRNAs (Chong-Morrison et al., 2018) in zebrafish. We first cloned sgRNAs targeting *Citrine* and *eGFP* into the Ac-Ds U6a mini-vector and injected reporter embryos with the corresponding sgRNA vector, Ac and Cas9 mRNAs. This led to widespread disruption of fluorescence (Figure S5), indicating the system robustly and efficiently perturbed *Citrine* and e*GFP* expression using somatic CRISPR-Cas9 genome editing.

Transglutaminase 2b (*tgm2b*), a gene enriched in Epi1 (Figure 4A), codes for a protein-crosslinking enzyme previously studied during bone formation (Deasey et al., 2012). HCR analysis showed that *tgm2b* transcripts were broadly distributed within the epicardium and co-localized with *tcf21* and *tbx18* mRNAs (Figures 4B–4B″). We designed *tgm2b* sgRNAs targeting the region coding for the active site of glutamyltransferase activity (Figures S6A and S6B). Somatic knockout of *tgm2b* led to defects in the epicardial cell sheet at 5 dpf (Figures 4C and 4D). In severe cases, large regions of the epicardial cell layer were missing, and epicardial cell numbers were significantly reduced in *tgm2b* knockout larvae (Figure 4E, $p = 0.0016$ and 4F, $p = 0.005$). We further established two stable *tgm2b* mutant lines, *tgm2b*$^{38ins/38ins}$ (mutant line 1, ox188, carrying 38-bp insertion in the *tgm2b* coding region) and *tgm2b*$^{7del1sub/7del1sub}$ (mutant line 2, ox189, carrying 7-bp deletion and 1-bp substitution) (Figure S6C). In both mutants, the resulting frameshift created an early translational termination site between WT residues 289 and 290 (length of WT tgm2b protein is 679 aa). *tgm2b* mutant larvae showed a reduction in epicardial cell numbers at 5 dpf, comparable with the phenotype observed in the transient *tgm2b* knockout (Figure 4E, $p_{wt\ versus\ homozyg} = 0.0011$ and 4F, $p_{wt\ versus\ homozyg} = 0.0003$). This phenotype might be due to a reduction in the number of epicardial cells that migrate from the PEO and attach to the myocardium, because the number of mutant *tcf21*$^{+}$ epicardial cells was already lowered at 66 hpf (Figures 4G–4I, $p_{somatic\ KO} = 0.0002$, $p_{wt\ versus\ homozyg} = 0.0051$), yet the numbers in the PEO of control and mutant embryos were similar (Figure 4J, $p_{somatic\ KO} = 0.5951$, $p_{wt\ versus\ homozyg} = 0.3964$). We tested whether *tgm2b* might inhibit epicardial cell proliferation (Figures 4K–4N). Control and *tgm2b* transient knockout embryos were incubated with EdU from 66 hpf to 77 hpf and, surprisingly, we observed an increased number of proliferating *tcf21*$^{+}$ cells in *tgm2b* knockout hearts. This suggests a compensatory mechanism for the decrease in epicardial cell numbers (Figures 4K–4M, $p = 0.0062$ and 4N, $p = 0.0352$).

Figure 3. Transcriptionally Distinct Epicardial Subpopulations Epi1–3 Localize to Different Regions of the Developing Heart

(A) Marker gene expression in Epi1–3. Cells were clustered in an unsupervised manner (columns). Color key indicates range of $\log_2$ transformed FPKM values. Genes analyzed further are highlighted in red. CM, cardiomyocyte; eHC, erythroid hematopoietic cell; mHC, myeloid hematopoietic cell; NC, neural cell; and MC, mesenchymal cell.

(B) mRNA staining of the Epi1 markers *adma* (magenta), *jam2b* (red), and *tcf21* (cyan) in a 5 dpf heart. (B′ and B″) A nucleus (asterisk) in the epicardial region surrounded by *adma*, *jam2b*, and *tcf21*.

(C) mRNA staining of the Epi2 marker *elnb* (magenta) and *tbx18* (green) at 5 dpf. (C′ and C″) Overlap of *elnb* and *tbx18* in the BA.

(D) Antibody staining of Mylka and *tbx18*:myr-Citrine at 5 dpf. (D′ and D″) Overlap of Mylka and myr-Citrine in the BA.

(E) mRNA staining of the Epi3 marker *cldn11a* (magenta) and *tcf21* (cyan) at 5 dpf. (E′ and E″) Two nuclei (asterisks) in the epicardial region between BA and atrium in close proximity to *cldn11a* and *tcf21*. Scale bars: 20 μm in (B)–(E) and 5 μm in (B′)–(E′) and (B″)–(E″). Color channels were adjusted separately for brightness and contrast. (B)–(E) are single optical sections. V, ventricle; BA, bulbus arteriosus.

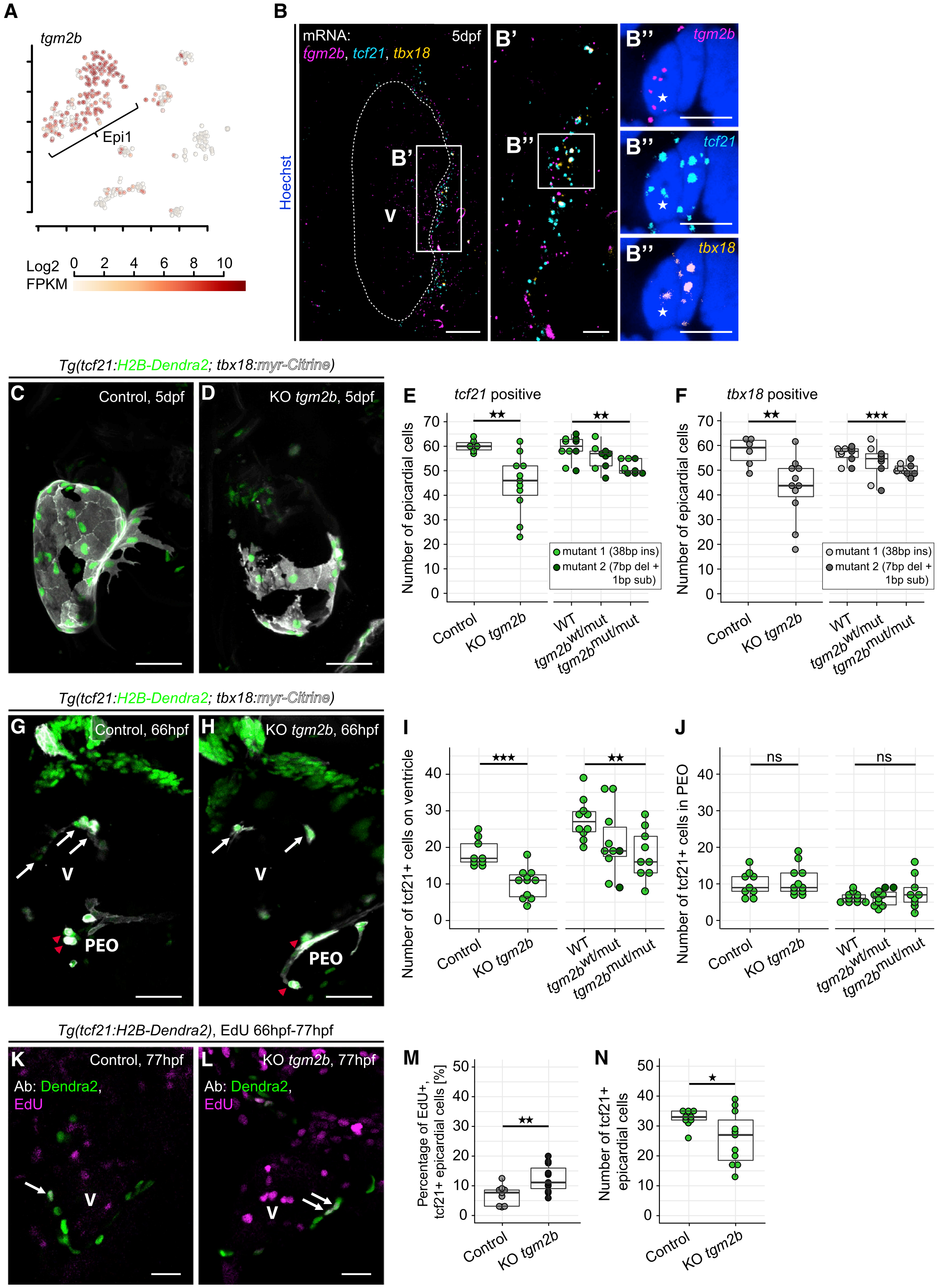

Figure 4. ***Transglutaminase 2b*** **Is Enriched in Epi1 and Functions to Maintain the Integrity of the Epicardial Cell Layer**

(A) Expression of *tgm2b*. Color key indicates range of $\log_2$ transformed FPKM values.

(B) mRNA staining of *tgm2b* (magenta), *tcf21* (cyan), and *tbx18* (orange) in a 5 dpf heart. (B′ and B″) A nucleus (asterisk) in the epicardial region surrounded by *tgm2b*, *tcf21*, and *tbx18*.

(C) The epicardium in a 5 dpf control larva.

(legend continued on next page)

In conclusion, *tgm2b* plays a critical role in maintaining the integrity of the forming epicardium and implicates Epi1 in ensuring the formation of a cohesive epithelial sheet of epicardial cells during heart development.

The Epi2-Enriched Gene *sema3fb* Regulates the Number of *tbx18*$^+$ Cells that Contribute to the Smooth Muscle Layer of the Outflow Tract

One of the most enriched factors in Epi2 was *sema3fb*, coding for a chemo-repellant known to be involved in neural patterning (Terriente et al., 2012) (Figure 5A). sema3fb belongs to the secreted class 3 semaphorins, which require a receptor complex consisting of a neuropilin (nrp1a/b or nrp2a/b) and a plexin for activity (reviewed in Neufeld et al. [2016]). We observed that *nrp1a* was co-expressed with *sema3fb* in Epi2, whereas most of the *nrp2a*$^+$ cells were assigned to Epi1 that also featured *nrp1a* expression (Figures 5B and 5C). HCR analysis revealed that *sema3fb* was strongly expressed within the BA and only sparsely in the surrounding areas (Figures 5D and 5D′). In contrast, *nrp2a* mRNA was almost exclusively located in areas surrounding the BA (Figure 5D, arrowheads). *nrp1a* mRNA was present in close proximity to *sema3fb* in the BA (Figure 5D, arrows) and also adjacent to the BA. At the BA boundary, both *nrp1a* and *nrp2a* were detected in close proximity to *tbx18* (Figure 5D′, asterisk) and *tcf21* (Figure 5E, asterisk in 5E′). In summary, these results suggest that cells within the BA express *sema3fb* and *nrp1a* (and not *nrp2a*), whereas cells outside the BA, such as those within the adjacent epicardial layer, express *nrp2a* and *nrp1a* (and not *sema3fb*).

The expression pattern of *sema3fb* and the *nrp* receptors, together with the association of Epi2 to cardiac cell migration (Fisher's exact test; $p = 0.00211$) (Figure 2D), suggested that Epi2 could be a source of repulsive guidance cues for other epicardial cells migrating into the BA. To test this hypothesis, we performed somatic mutagenesis of *sema3fb* (Figures S6D and S6E), which led to a significant increase in the number of *tbx18*$^+$ cells in the BA (Figures 5F–5H, $p = 0.0021$), supporting a role for *sema3fb* in restricting the number of Epi2 cells in the outflow tract. We investigated whether this function might involve inhibition of *tbx18*$^+$ cell proliferation in the BA (Figures S6F and S6G, arrows). However, there was no significant difference in the number of proliferating *tbx18*$^+$ cells, although many more *tbx18*:myr-Citrine$^+$ cells were detected within the BA in *sema3fb* knockouts (Figures S6F–S6H, $p = 0.8202$). This result argues against a role for *sema3fb* in restricting cell proliferation and suggests that it controls the number of *tbx18*$^+$ cells in the BA, by restricting their migration from surrounding tissues such as the epicardium.

We further isolated cells from *tbx18*:myr-Citrine$^+$*sema3fb* transient knockout hearts and performed scRNA-seq, adapting the recently established TARGET-seq method (Rodriguez-Meira et al., 2019). TARGET-seq allows the detection of mutations in both genomic DNA and transcriptome-derived cDNA at the single-cell level. We identified multiple types of *sema3fb*-gene-editing events in cells with sequencing read coverage of the *sema3fb* sgRNA target sites (Figures 5I–5K). Many of these (Figure 5I) were located in a cell cluster that was enriched in Epi2 markers (Figures 3A and S6I). However, other cells analyzed for *sema3fb* gene editing formed part of an additional cluster termed "KOsema3fb" only present in the *sema3fb* knockout dataset (Figure 5I). The KOsema3fb cluster did not feature the widespread presence of Epi1 or Epi2 markers, although multiple cells in this cluster expressed *tbx18* and *Citrine* mRNA (Figure S6I).

We found that 50% of the analyzed cells carried editing events, with both sgRNAs contributing to these (Figure 5J). Editing event types comprised insertions-deletions (indels) and nucleotide substitutions (Figure 5K). We analyzed *sema3fb* open reading frames (ORFs) after gene editing and found large deletions (Kosicki et al., 2018) and indel-induced frameshifts (Figure 5L), which reduced sema3fb protein length in over a third of the samples analyzed (Figure 5I). In addition, in up to 20% of the sequenced transcripts, we found aberrant splicing of *sema3fb* cDNA (Figures 5M and 5N), such as splicing across multiple *sema3fb* exons (Figure 5M, cell 033, arrow) or abnormal splicing from a possible cryptic intronic site (Figure 5M, cell 052, arrow) (Figure 5N). Aberrant splicing of *sema3fb* was absent in the non-knockout scRNA-seq dataset (Figure S6J), indicating that it was not an ordinarily occurring form of alternative splicing.

These results illustrate the impact of somatic *sema3fb* knockout on Epi2 cells and indicate that it reduces sema3fb functionality in around half of all *sema3fb*$^+$ cells.

Epi3-Enriched *cxcl12a* Attracts Leukocytes to the Developing Heart

Epi3 cells expressed the chemokine *cxcl12a* (Figure 6A). HCR revealed that the spatial distribution of *cxcl12a* transcripts was restricted to an area between BA and atrium (Figures 6B–6B″), very similar to *cldn11a* (Figure 3E), and a subset of *cxcl12a* was located in close proximity to *tcf21*, indicating co-expression.

(D) Disrupted epicardial integrity in a 5 dpf KO *tgm2b* larva.
(E) Absolute quantification of *tcf21*:H2B-Dendra2$^+$ epicardial cell numbers at 5 dpf in transient *tgm2b* knockouts (Control, KO *tgm2b*) and stable *tgm2b* mutants (heterozygous, *tgm2b*$^{wt/mut}$; homozygous, *tgm2b*$^{mut/mut}$). Larvae from two mutant lines were analyzed.
(F) Absolute quantification of *tbx18*:myr-Citrine$^+$ epicardial cell numbers at 5 dpf.
(G) The epicardium in a 66 hpf control embryo. White arrows indicate epicardial cells, and red arrowheads indicate cells in the PEO.
(H) Reduced epicardial cell numbers in a 66 hpf KO *tgm2b* embryo.
(I and J) Absolute quantification of *tcf21*:H2B-Dendra2$^+$ epicardial (I) and proepicardial (J) cell numbers at 66 hpf.
(K) Proliferation in the epicardium of a 77 hpf control larva (white arrows).
(L) Increased epicardial proliferation in a KO *tgm2b* larva.
(M) Relative quantification of proliferating epicardial cells.
(N) Absolute quantification of *tcf21*:H2B-Dendra2$^+$ epicardial cells. Scale bars: 50 μm in (C), (D), (G), and (H); 20 μm in (B); 10 μm in (K) and (L); and 5 μm in (B′) and (B″). Color channels were adjusted separately for brightness and contrast. (C), (D), (G), and (H) are projections; (B), (K), and (L) are single optical sections. Data are represented as median, first, and third quartiles (box). Significance was calculated using Welch's t test. *$p < 0.05$, **$p < 0.01$, ***$p < 0.001$. V, ventricle; Ab, antibody.
See also Figure S6.

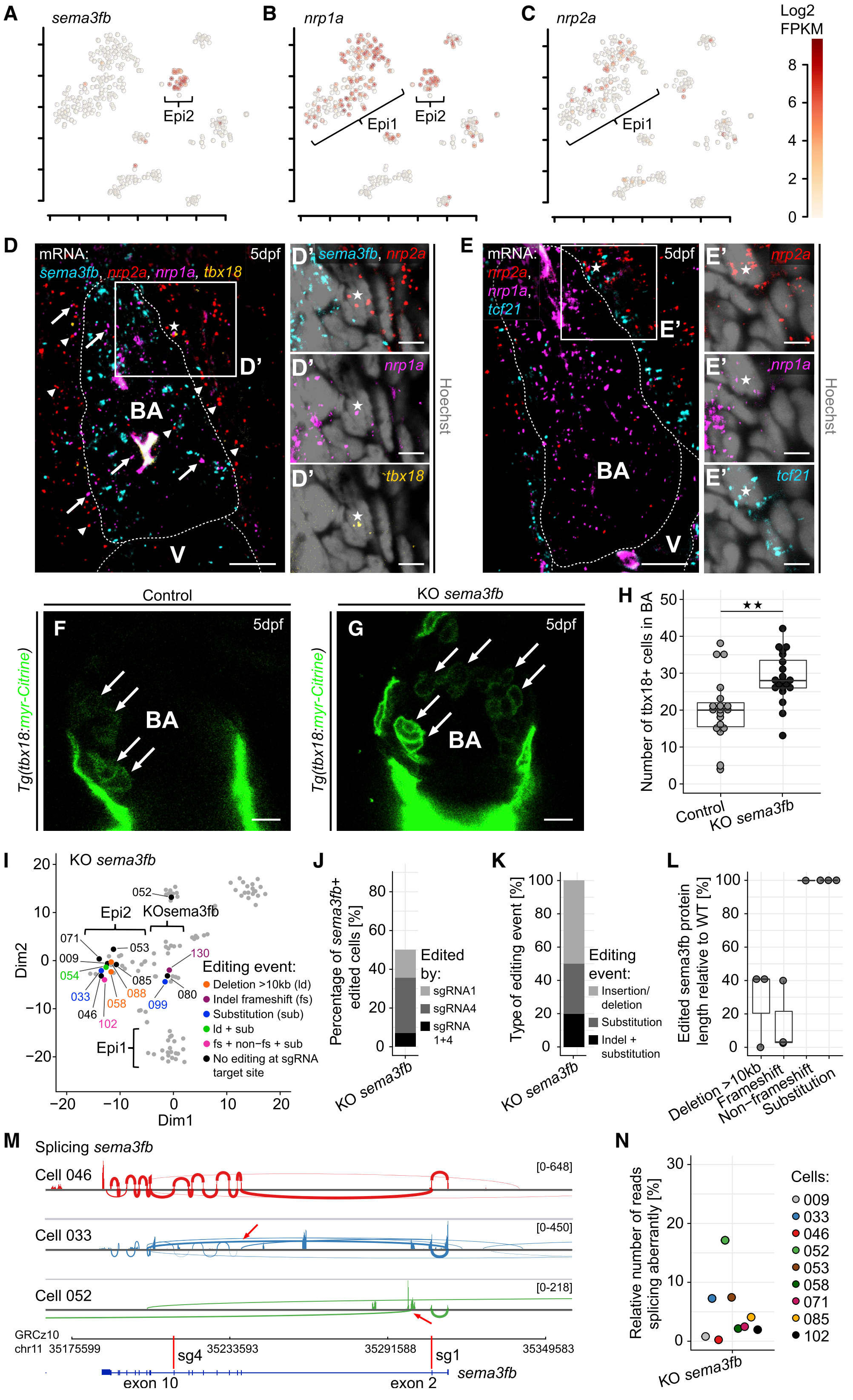

(legend on next page)

GO term analysis showed that Epi3 was enriched for genes involved in leukocyte chemotaxis (Fisher's exact test; $p = 0.0067$), a process also associated with the myeloid hematopoietic cell cluster (Fisher's exact test; $p = 7.2E{-}06$) (Figure 2D). Interestingly, the *cxcl12a* cognate receptor *cxcr4b* was strongly present in this population (Figure 6C, mHC), which was also enriched in *ptprc* (*CD45*) (Figure 6D), a pan-leukocyte marker in zebrafish (Bertrand et al., 2008). HCR revealed areas on the ventricular surface in which *cxcr4b* and *ptprc/CD45* were co-localized (Figures 6E and 6E′, asterisk). *tcf21* was present in neighboring regions but was not closely co-localizing with *cxcr4b* or *ptprc/CD45*. This suggested that epicardial cxcl12a might be attracting *ptprc/CD45*$^+$ leukocytes to the developing heart. We thus designed sgRNAs targeting the *cxcl12a* gene (Figures S7A and S7B). Somatic loss of *cxcl12a* decreased the number of *ptprc/CD45*:DsRed$^+$ cells (Bertrand et al., 2008) that were present on the surface of the heart (Figures 6F–6H, $p = 0.0003$). We observed direct contact between *ptprc/CD45*:DsRed$^+$ cells and the outer epicardial surface in controls (arrows in Figures 6F′ and 6F″) and reduced contacts in *cxcl12a* knockout larvae (Figure 6G′). We next established a stable mutant line featuring a 15-bp deletion at the start of the *cxcl12a* coding sequence, *cxcl12a*$^{15del/15del}$ (ox190) (Figure S7C). Homozygous mutant *cxcl12a*$^{15del/15del}$ embryos showed a defect in lateral line development, recapitulating a previously published *cxcl12a* mutant phenotype arising from a different mutation (Valentin et al., 2007) (Figures S7D–S7F). Importantly, the number of *ptprc/CD45*:DsRed$^+$ cells in contact with the epicardium was significantly reduced in *cxcl12a* mutant larvae (Figure 6H, $p_{wt\ versus\ homozyg} = 0.0042$), consistent with our somatic *cxcl12a* knockout experiments. These data identify a role for *cxcl12a* in chemo-attracting leukocytes to the surface of the developing heart.

RNA-seq analysis of *cxcl12a* mRNA in single transient knockout larvae detected a variety of gene editing events (Figures 6I and 6J), with indels only detected in *cxcl12a* knockouts. Around 5% of the reads at the target site of *cxcl12a* sgRNA1 were edited (Figure 6I), whereas the editing frequency at the *cxcl12a* sgRNA3 target site was around 30% (Figure 6J). Furthermore, a significant number of reads spliced aberrantly from *cxcl12a* exon 1 into exon 3 in the *cxcl12a* transient knockouts (Figures 6J and 6K, arrows, and 6L). Additionally, we found that most indels should result in shortened *cxcl12a* ORFs (Figures S7G and S7H), with aberrant splicing reducing the *cxcl12a* ORF to only 25% of its non-edited length (Figure S7H). Despite the identified mutations, no significant reduction in *cxcl12a* mRNA levels was detected (Figure S7I, $p = 0.2221$).

In summary, we found clear evidence of somatic *cxcl12a* gene editing. All *cxcl12a*-mutated larvae featured indels at both sgRNA target sites, as well as aberrant *cxcl12a* splicing, which likely affected cxcl12a protein size and function.

DISCUSSION

Understanding epicardial cell fate and lineage potential to date has been constrained by the limited set of so-called canonical markers which are either, not entirely specific for epicardial cells or, fail to label the entire epicardial cell pool. Here, we identify epicardial cell subpopulations within the developing zebrafish heart, characterize their transcriptional profiles at the single-cell level, and functionally test their involvement during heart development in zebrafish.

Our study provides insights into the heterogeneity and associated functional diversity of the developing epicardium. We identify the epicardial cell populations Epi1, Epi2, and Epi3, their distinct genetic programs and spatial distributions (Figure 7A). GO analysis of the most highly enriched genes in each of these clusters and perturbation of signature genes provided insights into the maintenance of epicardial integrity, formation of the outflow tract, and myeloid cell recruitment into the developing heart. Because of the relatively low cell numbers analyzed in our dataset, there might, however, be additional scarce epicardial subpopulations that remained undetected.

Maintenance of Epithelial Integrity within the Forming Epicardium

GO term analysis of Epi1 genes indicated a possible Epi1 role in the formation of a coherent epicardial cell sheet that migrates to envelope the myocardium. We focused on *tgm2b*, whose functional perturbation decreased epicardial cell numbers (Figure 7B). Interestingly, cell numbers in the PEO were unaffected by *tgm2b* knockout (Figure 4J), arguing against an early specification defect. Similarly, defective proliferation in the early epicardium was excluded as a possible cause for the reduction in epicardial cell numbers (Figures 4K–4M). Instead, there might be a reduction in the number of pro-epicardial cells that are

Figure 5. *Semaphorin 3fb* Is a Marker of Epi2 and Controls the Number of *tbx18*$^+$ Cells in the Bulbus Arteriosus

(A–C) Expression of *sema3fb* (A), *nrp1a* (B), and *nrp2a* (C). Color key indicates range of log_2 transformed FPKM values.

(D) mRNA staining of *sema3fb* (cyan), *nrp2a* (red, arrowheads), *nrp1a* (magenta, arrows), and *tbx18* (orange) at 5 dpf. (D′) Close proximity of *nrp2a*, *nrp1a,* and *tbx18* to a nucleus (asterisk) at the BA boundary.

(E) mRNA staining of *nrp2a* (red), *nrp1a* (magenta), and *tcf21* (cyan) at 5 dpf. (E′) A nucleus (asterisk) in close proximity to *nrp2a*, *nrp1a*, and *tcf21*.

(F) The BA in a 5 dpf control larva.

(G) Increased numbers of *tbx18*:myr-Citrine$^+$ cells (arrows) in the BA of a KO *sema3fb* larva.

(H) Absolute quantification of *tbx18*:myr-Citrine$^+$ cell numbers in the BA at 5 dpf.

(I–N) Analysis of gene editing in single *sema3fb*-mutated epicardial cells. (I) t-SNE plot showing cell clusters and editing events. Numbers indicate cell identities, and colors depict editing events. Ld, large deletion; fs, frameshift; and sub, substitution. (J) Relative number of Cas9-edited *sema3fb*$^+$ cells. Colors indicate the *sema3fb* sgRNA target site edited. (K) Frequency of editing event types. (L) Inferred edited sema3fb protein lengths, relative to WT. (M) Sashimi plots showing splicing of *sema3fb*. Arrows highlight reads splicing abnormally. Red bars indicate sgRNA target sites. (N) Relative number of *sema3fb* splicing reads that spliced abnormally.

Scale bars: 10 μm in (D)–(G) and 5 μm in (D′) and (E′). Color channels were adjusted separately for brightness and contrast. (D)–(G) are single optical sections. Data in (H) and (L) are represented as median, first, and third quartiles (box). Significance calculated using Welch's t test. $^{**}p < 0.01$. V, ventricle; BA, bulbus arteriosus. See also Figure S6.

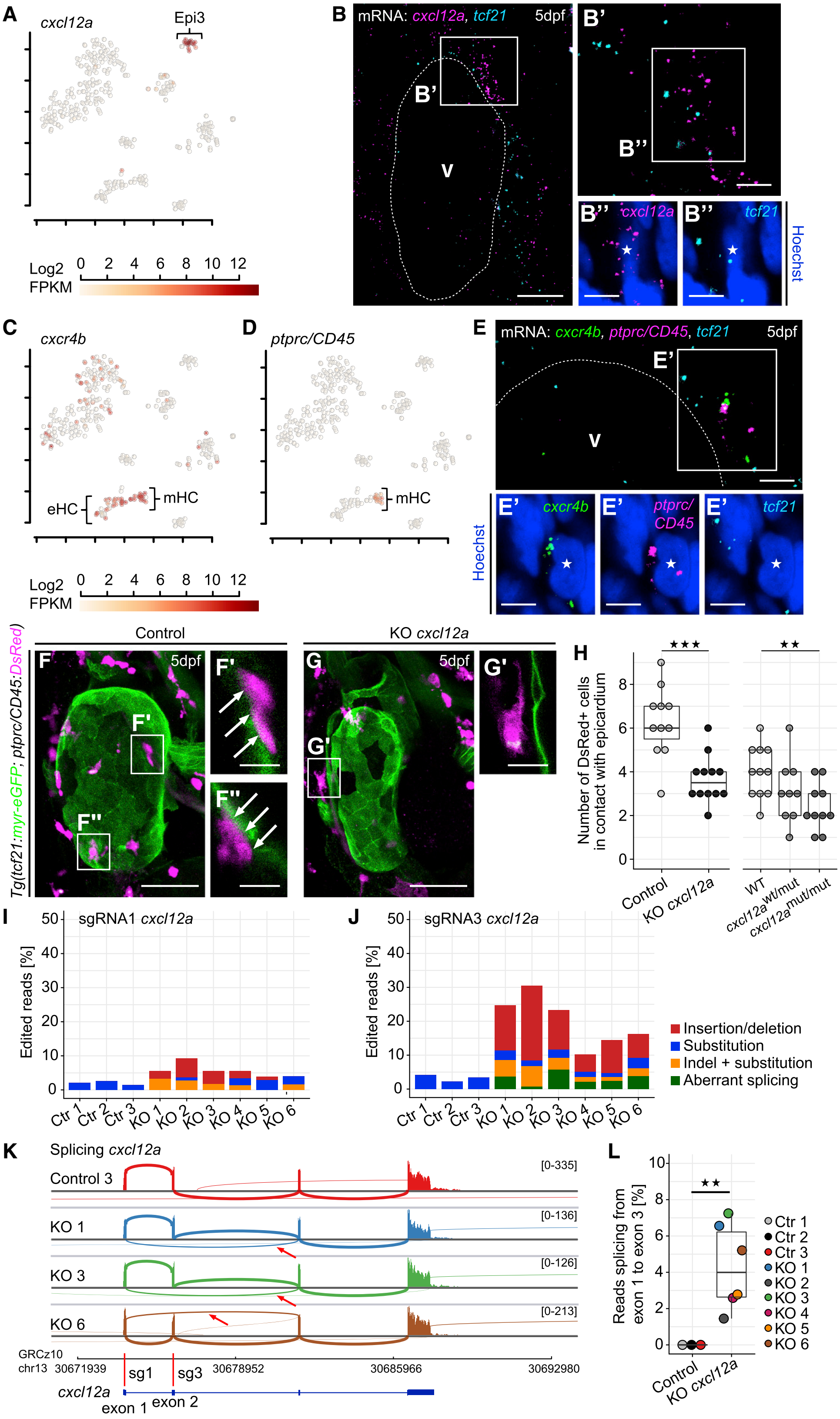

(legend on next page)

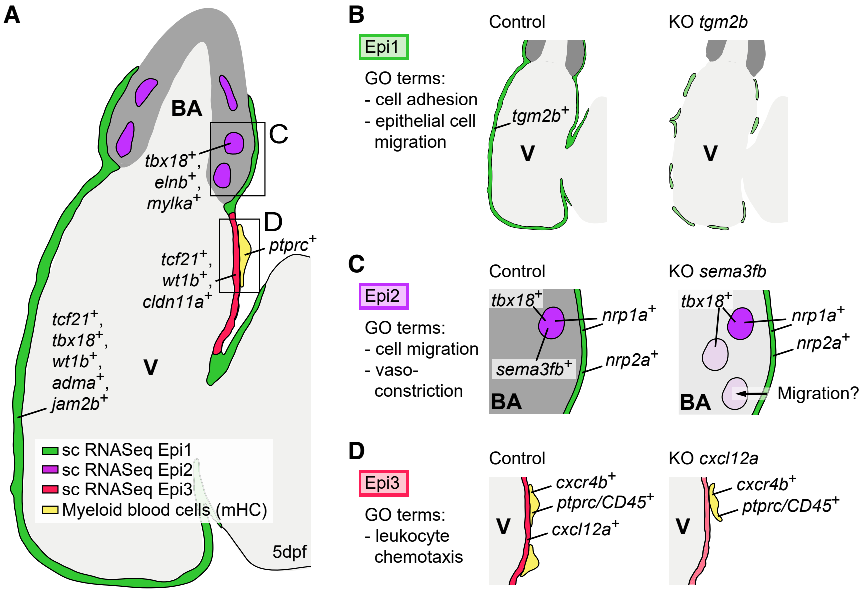

Figure 7. Summary of the Distinct Epicardial Cell Populations in the Developing Zebrafish Heart

(A) Zebrafish heart at 5 dpf, showing the distributions of Epi1-3.

(B) Loss of *tgm2b* disrupts the integrity of the epicardial cell layer.

(C) Loss of *sema3fb* increases the number of Epi2 cells in the BA, possibly owing to increased cell migration from the epicardium.

(D) Loss of *cxcl12a* decreases the number of myeloid cells on the heart. V, ventricle; BA, bulbus arteriosus.

able to attach to the myocardium during epicardium formation (Kwee et al., 1995; Sengbusch et al., 2002; Yang et al., 1995), a process also known to require heartbeat-derived fluid flow (Peralta et al., 2013). Additionally, *tgm2b* might be involved in the regulation of cell-cell contacts within the epicardial cell layer.

The Regulation of Epicardial Cell Migration into the Outflow Tract

GO term analysis of Epi2-enriched genes suggested that this subpopulation might be linked to the smooth muscle wall of the BA. Indeed, many Epi2-specific genes were exclusively expressed in the developing smooth muscle layer of the BA. We show that these *tbx18*$^+$ Epi2 cells had a *tcf21* origin but never expressed *wt1b*. Pseudotime analysis suggested that a subset of Epi1 cells might be in a transition state toward Epi2 (Figures S4A and S4B), supported by our *tcf21* lineage tracing results. The BA of the adult zebrafish heart is a positive regulator of epicardial cell migration (Wang et al., 2015). Following cardiac injury, the BA is thought to drive the coordinated movement of the regenerating epicardial cell sheet via Sonic hedgehog signaling (Wang et al., 2015). The secreted diffusible chemorepulsive factor sema3fb is enriched in Epi2 and functions to negatively regulate the number of epicardial cells that populate the BA (Figure 7C), therefore, acting as a gatekeeper controlling the number of cells in the outflow tract. Both sema3 signaling receptors, nrp1a and nrp2a, were expressed in the epicardium. NRP1 and NRP2 have been reported to interact with SEMA3 ligands differentially, with SEMA3F specifically binding to NRP2 and SEMA3A partnering with NRP1 (reviewed in Neufeld et al. [2016]). The fact that *nrp2a* transcripts were abundant in the vicinity of the BA, but excluded from the inside, supports a model in which nrp2a$^+$ Epi1 cells are retained outside of the BA by the chemorepulsive activity of sema3fb, whereas nrp2a$^-$ epicardial cells are able to migrate into and home to the BA. In transient *sema3fb* knockouts, we identified a *sema3fb*$^+$, partially *tbx18*$^+$ cell cluster not present in the WT setting (Figures 3A and S6I), which may represent a population of cells that have abnormally homed to the BA. Our work highlights the importance of understanding the mechanisms enabling the migration of epicardial cells in response to diffusible guidance cues.

Epicardial Cell Regulation of Leukocyte Homing into the Heart

Epi3 might be involved in the guidance of white blood cells into the developing heart. Links between the epicardium and the

Figure 6. The Chemokine *cxcl12a* Is Expressed in Epi3 and Attracts *ptprc*$^+$ Leukocytes to the Epicardium

(A) Expression of *cxcl12a*. Color key indicates range of $\log_2$ transformed FPKM values.

(B) mRNA staining of *cxcl12a* (magenta) and *tcf21* (cyan) at 5 dpf. (B′ and B″) A nucleus (asterisk) in the epicardial region between BA and atrium surrounded by *cxcl12a* and *tcf21*.

(C and D) Expression of *cxcr4b* (C) and *ptprc* (D). Color key indicates range of $\log_2$ transformed FPKM values.

(E) mRNA staining of *cxcr4b* (green), *ptprc* (magenta), and *tcf21* (cyan) at 5 dpf. (E′) A nucleus (asterisk) in close proximity to *cxcr4b* and *ptprc*.

(F) The heart in a 5 dpf control larva. (F′ and F″) *ptprc/Cd45*:DsRed$^+$ cells in contact with the epicardium (arrows).

(G and G′) Fewer *ptprc/CD45*:DsRed$^+$ cells on the heart of a 5 dpf KO *cxcl12a* larva.

(H) Absolute quantification of *ptprc/CD45*:DsRed$^+$ cells in contact with the epicardium in transient *cxcl12a* knockouts (Control, KO *cxcl12a*) and stable *cxcl12a* mutants (heterozygous, *cxcl12a*$^{wt/mut}$; homozygous, *cxcl12a*$^{mut/mut}$).

(I and J) RNA-seq analysis of *cxcl12a* gene editing in single control (ctr) and *cxcl12a*-knockout (KO) larvae. Relative numbers of edited reads that spanned the target site of *cxcl12a* sgRNA1 (I) or sgRNA3 (J).

(K) Sashimi plots showing splicing of *cxcl12a*. Arrows highlight reads splicing from exon 1 into exon 3. Red bars indicate sgRNA target sites.

(L) Relative number of reads splicing from *cxcl12a* exon 1 that spliced from exon 1 into exon 3.

Scale bars: 50 μm in (F) and (G); 20 μm in (B); 10 μm in (F′), (F″), and (G′), and 5 μm in (B′), (B″), (E), and (E′). Color channels were adjusted separately for brightness and contrast. (F) and (G) are projections; (B), (E), (F′), (F″), and (G′) are single optical sections. Data in (H) and (L) are represented as median, first, and third quartiles (box). Significance calculated using Welch's t test. **p < 0.01, ***p < 0.001. V, ventricle; eHC, erythroid hematopoietic cell; mHC, myeloid hematopoietic cell.

See also Figure S7.

immune system have been implicated during mouse fetal heart development, where CD68$^+$ macrophages required the intact epicardium for their recruitment into the developing heart, a process dependent on the expression of *Wt1* (Stevens et al., 2016). Our study similarly reveals that the Epi3-enriched chemokine *cxcl12a* is necessary for the recruitment and/or retention of *ptprc/CD45*$^+$ hematopoietic cells onto the surface of the heart (Figure 7D). These leukocyte or myeloid cells express the cognate chemokine receptor *cxcr4b*. Although there appear to be only a few Epi3 cells in the epicardium, their strong expression of the diffusible *cxcl12a* in the confined space of the pericardial cavity is likely to affect cxcl12a responsive cells in the vicinity, such as the *cxcr4b*$^+$/*CD45*$^+$ cells we analyzed. Thus, our work unravels a developmental mechanism by which an epicardial subpopulation regulates leukocyte recruitment through the expression of a paracrine signaling molecule. Non-PEO-derived CD45$^+$ hematopoietic cells were shown to contribute to the developing epicardium of the mouse heart (Balmer et al., 2014). Therefore, it will be of interest to further explore the possible role of *cxcr4b*$^+$/*CD45*$^+$ cells present on the developing zebrafish heart. *Cxcl12a* is also expressed in adult epicardial cells upon cardiac injury (Itou et al., 2012), and, in the mouse, epicardial-associated CD45$^+$ hematopoietic cell clusters respond dynamically to myocardial infarction (Balmer et al., 2014). These cells co-express CD45$^+$/*CXCR4*$^+$/*CD11b*$^+$ in the bone marrow and within the ischemic heart (Ghadge et al., 2017), suggesting that *cxcl12a* may recapitulate its developmental role following adult heart injury.

In conclusion, our scRNA-seq work has opened up avenues for understanding epicardial cell biology and provides insights into the formation of a critical lineage, which is essential during both heart development and adult heart regeneration. The identification of functional subpopulations of epicardial cells and their roles as sources of chemo-attractant and repellent signals for cellular cross-talk during development may be exploited to facilitate cell-based therapies to regenerate the injured heart.

STAR★METHODS

Detailed methods are provided in the online version of this paper and include the following:

- KEY RESOURCES TABLE
- LEAD CONTACT AND MATERIALS AVAILABILITY
- EXPERIMENTAL MODEL AND SUBJECT DETAILS
  - Zebrafish Lines
- METHOD DETAILS
  - Generation of Transgenic Zebrafish Lines
  - Larval Heart Isolation, Dissociation and FAC-Sorting
  - Single Cell Library Preparation and Sequencing
  - KO *sema3fb* Single Cell Library Preparation and Sequencing
  - KO *cxcl12a* Single Embryo Library Preparation and Sequencing
  - Hybridisation Chain Reaction
  - Immunocytochemistry
  - EdU Staining
  - Lineage Tracing
  - Transient Cas9-Mediated Gene Knockout
  - Cas9 Activity Efficiency Test
  - Generation of Stable Mutant Zebrafish Lines
  - Bioinformatic Processing
- QUANTIFICATION AND STATISTICAL ANALYSIS
- DATA AND CODE AVAILABILITY

SUPPLEMENTAL INFORMATION

Supplemental Information can be found online at https://doi.org/10.1016/j.devcel.2020.01.023.

ACKNOWLEDGMENTS

We would like to thank the Biomedical Services Unit for fish husbandry as well as MRC WIMM Flow Cytometry Facility, MRC WIMM Sequencing Facility, MRC WIMM Single Cell Core Facility, and the Wolfson Imaging Center Oxford for excellent services. We thank Alba Rodriguez-Meira and Adam Mead for sharing their TARGET-seq protocol. We would also like to thank Vanessa Chong-Morrison for providing us with the zebrafish U6a promoter Ac-Ds sgRNA expression vector. *Tg(tcf21:DsRed2)^{pd37}* and *Tg(tbx18:DsRed2)^{pd22}* lines were kindly provided by Ken Poss, *Tg(wt1b:eGFP)^{li1}* by Christoph Englert, *Tg(ptprc/CD45:DsRed)^{sd3}* by David Traver, and *Tg(ubi:CSY)* by Marianne Voz. *Tg(ubi:Switch)* was obtained from the Zebrafish International Resource Center (ZIRC). This work was supported by a British Heart Foundation (BHF) 4-year PhD studentship FS/13/57/30647 to M.W., an Oxford BHF Centre of Research Excellence fellowship RE/13/1/30181 award to F.C.S., MRC-MHU grant to R.P., MRC G0902418 grant and Oxford BHF CRE RE/08/004 award to T.S.-S., and BHF grants RG/13/9/303269 and CH/11/1/28798 to P.R.R.

AUTHOR CONTRIBUTIONS

Conceptualization, F.C.S., T.S.-S., and P.R.R.; Methodology, M.W. and F.C.S.; Investigation, M.W. and F.C.S.; Writing – Original Draft, M.W. and F.C.S.; Writing – Review & Editing, T.S.-S. and P.R.R.; Bioinformatic Analysis and Data Curation, M.W.; Supervision, F.C.S., T.S.-S., and P.R.R.; Funding Acquisition, R.P., T.S.-S., and P.R.R.

DECLARATION OF INTERESTS

P.R.R. is co-founder and equity holder in OxStem Cardio, an Oxford University spinout that seeks to exploit therapeutic strategies stimulating endogenous repair in cardiovascular regenerative medicine.

Received: October 16, 2018
Revised: December 7, 2019
Accepted: January 22, 2020
Published: February 20, 2020

REFERENCES

Acharya, A., Baek, S.T., Huang, G., Eskiocak, B., Goetsch, S., Sung, C.Y., Banfi, S., Sauer, M.F., Olsen, G.S., Duffield, J.S., et al. (2012). The bHLH transcription factor Tcf21 is required for lineage-specific EMT of cardiac fibroblast progenitors. Development *139*, 2139–2149.

Alexa, A., and Rahnenfuehrer, J. (2019). Gene set enrichment analysis with topGO. https://bioconductor.org/packages/release/bioc/vignettes/topGO/inst/doc/topGO.pdf.

Balmer, G.M., Bollini, S., Dubé, K.N., Martinez-Barbera, J.P., Williams, O., and Riley, P.R. (2014). Dynamic haematopoietic cell contribution to the developing and adult epicardium. Nat. Commun. *5*, 4054.

Beis, D., Bartman, T., Jin, S.W., Scott, I.C., D'Amico, L.A., Ober, E.A., Verkade, H., Frantsve, J., Field, H.A., Wehman, A., et al. (2005). Genetic and cellular analyses of zebrafish atrioventricular cushion and valve development. Development *132*, 4193–4204.

Bertrand, J.Y., Kim, A.D., Teng, S., and Traver, D. (2008). CD41+ cmyb+ precursors colonize the zebrafish pronephros by a novel migration route to initiate adult hematopoiesis. Development *135*, 1853–1862.

Braitsch, C.M., Combs, M.D., Quaggin, S.E., and Yutzey, K.E. (2012). Pod1/Tcf21 is regulated by retinoic acid signaling and inhibits differentiation of epicardium-derived cells into smooth muscle in the developing heart. Dev. Biol. *368*, 345–357.

Burger, A., Lindsay, H., Felker, A., Hess, C., Anders, C., Chiavacci, E., Zaugg, J., Weber, L.M., Catena, R., Jinek, M., et al. (2016). Maximizing mutagenesis with solubilized CRISPR-Cas9 ribonucleoprotein complexes. Development *143*, 2025–2037.

Burns, C.G., and MacRae, C.A. (2006). Purification of hearts from zebrafish embryos. BioTechniques *40*, 274–282.

Bussmann, J., and Schulte-Merker, S. (2011). Rapid BAC selection for tol2-mediated transgenesis in zebrafish. Development *138*, 4327–4332.

Cai, C.L., Martin, J.C., Sun, Y., Cui, L., Wang, L., Ouyang, K., Yang, L., Bu, L., Liang, X., Zhang, X., et al. (2008). A myocardial lineage derives from Tbx18 epicardial cells. Nature *454*, 104–108.

Chau, Y.Y., Bandiera, R., Serrels, A., Martínez-Estrada, O.M., Qing, W., Lee, M., Slight, J., Thornburn, A., Berry, R., Mchaffie, S., et al. (2014). Visceral and subcutaneous fat have different origins and evidence supports a mesothelial source. Nat. Cell Biol. *16*, 367–375.

Choi, H.M., Chang, J.Y., Trinh, le A., Padilla, J.E., Fraser, S.E., and Pierce, N.A. (2010). Programmable in situ amplification for multiplexed imaging of mRNA expression. Nat. Biotechnol. *28*, 1208–1212.

Choi, H.M.T., Schwarzkopf, M., Fornace, M.E., Acharya, A., Artavanis, G., Stegmaier, J., Cunha, A., and Pierce, N.A. (2018). Third-generation in situ hybridization chain reaction: multiplexed, quantitative, sensitive, versatile, robust. Development *145*, dev165753.

Chong-Morrison, V., Simões, F.C., Senanayake, U., Carroll, D.S., Riley, P.R., and Sauka-Spengler, T. (2018). Re-purposing Ac/Ds transgenic system for CRISPR/dCas9 modulation of enhancers and non-coding RNAs in zebrafish. bioRxiv. https://doi.org/10.1101/450684.

Davis, J., Maillet, M., Miano, J.M., and Molkentin, J.D. (2012). Lost in transgenesis: a user's guide for genetically manipulating the mouse in cardiac research. Circ. Res. *111*, 761–777.

Deasey, S., Grichenko, O., Du, S., and Nurminskaya, M. (2012). Characterization of the transglutaminase gene family in zebrafish and in vivo analysis of transglutaminase-dependent bone mineralization. Amino Acids *42*, 1065–1075.

del Monte, G., Casanova, J.C., Guadix, J.A., MacGrogan, D., Burch, J.B., Pérez-Pomares, J.M., and de La Pompa, J.L. (2011). Differential Notch signaling in the epicardium is required for cardiac inflow development and coronary vessel morphogenesis. Circ. Res. *108*, 824–836.

Dempsey, W.P., Fraser, S.E., and Pantazis, P. (2012). PhOTO zebrafish: a transgenic resource for in vivo lineage tracing during development and regeneration. PLoS One *7*, e32888.

Dettman, R.W., Denetclaw, W., Jr., Ordahl, C.P., and Bristow, J. (1998). Common epicardial origin of coronary vascular smooth muscle, perivascular fibroblasts, and intermyocardial fibroblasts in the avian heart. Dev. Biol. *193*, 169–181.

Dobin, A., Davis, C.A., Schlesinger, F., Drenkow, J., Zaleski, C., Jha, S., Batut, P., Chaisson, M., and Gingeras, T.R. (2013). STAR: ultrafast universal RNA-seq aligner. Bioinformatics *29*, 15–21.

Emelyanov, A., Gao, Y., Naqvi, N.I., and Parinov, S. (2006). Trans-kingdom transposition of the maize dissociation element. Genetics *174*, 1095–1104.

Fan, J., Salathia, N., Liu, R., Kaeser, G.E., Yung, Y.C., Herman, J.L., Kaper, F., Fan, J.B., Zhang, K., Chun, J., and Kharchenko, P.V. (2016). Characterizing transcriptional heterogeneity through pathway and gene set overdispersion analysis. Nat. Methods *13*, 241–244.

Gambardella, L., McManus, S.A., Moignard, V., Sebukhan, D., Delaune, A., Andrews, S., Bernard, W.G., Morrison, M.A., Riley, P.R., Göttgens, B., et al. (2019). BNC1 regulates cell heterogeneity in human pluripotent stem cell-derived epicardium. Development *146*, dev174441.

Ghadge, S.K., Messner, M., Van Pham, T., Doppelhammer, M., Petry, A., Görlach, A., Husse, B., Franz, W.M., and Zaruba, M.M. (2017). Prolyl-hydroxylase inhibition induces SDF-1 associated with increased CXCR4+/CD11b+ subpopulations and cardiac repair. J. Mol. Med. (Berl.) *95*, 825–837.

González-Rosa, J.M., Peralta, M., and Mercader, N. (2012). Pan-epicardial lineage tracing reveals that epicardium derived cells give rise to myofibroblasts and perivascular cells during zebrafish heart regeneration. Dev. Biol. *370*, 173–186.

Gow, A., Southwood, C.M., Li, J.S., Pariali, M., Riordan, G.P., Brodie, S.E., Danias, J., Bronstein, J.M., Kachar, B., and Lazzarini, R.A. (1999). CNS myelin and Sertoli cell tight junction strands are absent in Osp/claudin-11 null mice. Cell *99*, 649–659.

Guadix, J.A., Carmona, R., Muñoz-Chápuli, R., and Pérez-Pomares, J.M. (2006). In vivo and in vitro analysis of the vasculogenic potential of avian proepicardial and epicardial cells. Dev. Dyn. *235*, 1014–1026.

Hendel, A., Bak, R.O., Clark, J.T., Kennedy, A.B., Ryan, D.E., Roy, S., Steinfeld, I., Lunstad, B.D., Kaiser, R.J., Wilkens, A.B., et al. (2015). Chemically modified guide RNAs enhance CRISPR-Cas genome editing in human primary cells. Nat. Biotechnol. *33*, 985–989.

Huang, C.J., Tu, C.T., Hsiao, C.D., Hsieh, F.J., and Tsai, H.J. (2003). Germ-line transmission of a myocardium-specific GFP transgene reveals critical regulatory elements in the cardiac myosin light chain 2 promoter of zebrafish. Dev. Dyn. *228*, 30–40.

Itou, J., Oishi, I., Kawakami, H., Glass, T.J., Richter, J., Johnson, A., Lund, T.C., and Kawakami, Y. (2012). Migration of cardiomyocytes is essential for heart regeneration in zebrafish. Development *139*, 4133–4142.

Katz, T.C., Singh, M.K., Degenhardt, K., Rivera-Feliciano, J., Johnson, R.L., Epstein, J.A., and Tabin, C.J. (2012). Distinct compartments of the proepicardial organ give rise to coronary vascular endothelial cells. Dev. Cell *22*, 639–650.

Kell, M.J., Riccio, R.E., Baumgartner, E.A., Compton, Z.J., Pecorin, P.J., Mitchell, T.A., Topczewski, J., and Leclair, E.E. (2018). Targeted deletion of the zebrafish actin-bundling protein L-plastin (lcp1). PLoS One *13*, e0190353.

Kharchenko, P.V., Silberstein, L., and Scadden, D.T. (2014). Bayesian approach to single-cell differential expression analysis. Nat. Methods *11*, 740–742.

Kikuchi, K., Gupta, V., Wang, J., Holdway, J.E., Wills, A.A., Fang, Y., and Poss, K.D. (2011). tcf21+ epicardial cells adopt non-myocardial fates during zebrafish heart development and regeneration. Development *138*, 2895–2902.

Kosicki, M., Tomberg, K., and Bradley, A. (2018). Repair of double-strand breaks induced by CRISPR-Cas9 leads to large deletions and complex rearrangements. Nat. Biotechnol. *36*, 765–771.

Krijthe, J.H. (2015). Rtsne: T-distributed stochastic neighbor embedding using a Barnes-Hut implementation [Online]. https://github.com/jkrijthe/Rtsne.

Kwee, L., Baldwin, H.S., Shen, H.M., Stewart, C.L., Buck, C., Buck, C.A., and Labow, M.A. (1995). Defective development of the embryonic and extraembryonic circulatory systems in vascular cell adhesion molecule (VCAM-1) deficient mice. Development *121*, 489–503.

La Manno, G., Soldatov, R., Zeisel, A., Braun, E., Hochgerner, H., Petukhov, V., Lidschreiber, K., Kastriti, M.E., Lönnerberg, P., Furlan, A., et al. (2018). RNA velocity of single cells. Nature *560*, 494–498.

Lepilina, A., Coon, A.N., Kikuchi, K., Holdway, J.E., Roberts, R.W., Burns, C.G., and Poss, K.D. (2006). A dynamic epicardial injury response supports progenitor cell activity during zebrafish heart regeneration. Cell *127*, 607–619.

Li, H., Handsaker, B., Wysoker, A., Fennell, T., Ruan, J., Homer, N., Marth, G., Abecasis, G., and Durbin, R.; 1000 Genome Project Data Processing Subgroup (2009). The Sequence Alignment/Map format and SAMtools. Bioinformatics *25*, 2078–2079.

Liao, Y., Smyth, G.K., and Shi, W. (2014). featureCounts: an efficient general purpose program for assigning sequence reads to genomic features. Bioinformatics *30*, 923–930.

Liu, Q., Huang, X., Oh, J.H., Lin, R.Z., Duan, S., Yu, Y., Yang, R., Qiu, J., Melero-Martin, J.M., Pu, W.T., and Zhou, B. (2014). Epicardium-to-fat transition in injured heart. Cell Res. *24*, 1367–1369.

Lyons, S.E., Lawson, N.D., Lei, L., Bennett, P.E., Weinstein, B.M., and Liu, P.P. (2002). A nonsense mutation in zebrafish gata1 causes the bloodless phenotype in vlad tepes. Proc. Natl. Acad. Sci. USA *99*, 5454–5459.

Männer, J. (1999). Does the subepicardial mesenchyme contribute myocardioblasts to the myocardium of the chick embryo heart? A quail-chick chimera study tracing the fate of the epicardial primordium. Anat. Rec. *255*, 212–226.

Männer, J., Pérez-Pomares, J.M., Macías, D., and Muñoz-Chápuli, R. (2001). The origin, formation and developmental significance of the epicardium: a review. Cells Tissues Organs *169*, 89–103.

McCarthy, D.J., Campbell, K.R., Lun, A.T., and Wills, Q.F. (2017). Scater: pre-processing, quality control, normalization and visualization of single-cell RNA-seq data in R. Bioinformatics *33*, 1179–1186.

McCLINTOCK, B. (1950). The origin and behavior of mutable loci in maize. Proc. Natl. Acad. Sci. USA *36*, 344–355.

Mikawa, T., and Fischman, D.A. (1992). Retroviral analysis of cardiac morphogenesis: discontinuous formation of coronary vessels. Proc. Natl. Acad. Sci. USA *89*, 9504–9508.

Mikawa, T., and Gourdie, R.G. (1996). Pericardial mesoderm generates a population of coronary smooth muscle cells migrating into the heart along with ingrowth of the epicardial organ. Dev. Biol. *174*, 221–232.

Moriyama, Y., Ito, F., Takeda, H., Yano, T., Okabe, M., Kuraku, S., Keeley, F.W., and Koshiba-Takeuchi, K. (2016). Evolution of the fish heart by sub/neo-functionalization of an elastin gene. Nat. Commun. *7*, 10397.

Mosimann, C., Kaufman, C.K., Li, P., Pugach, E.K., Tamplin, O.J., and Zon, L.I. (2011). Ubiquitous transgene expression and Cre-based recombination driven by the ubiquitin promoter in zebrafish. Development *138*, 169–177.

Neufeld, G., Mumblat, Y., Smolkin, T., Toledano, S., Nir-Zvi, I., Ziv, K., and Kessler, O. (2016). The role of the semaphorins in cancer. Cell Adh. Migr. *10*, 652–674.

Otte, K., Kranz, H., Kober, I., Thompson, P., Hoefer, M., Haubold, B., Remmel, B., Voss, H., Kaiser, C., Albers, M., et al. (2003). Identification of farnesoid X receptor beta as a novel mammalian nuclear receptor sensing lanosterol. Mol. Cell. Biol. *23*, 864–872.

Park, H.C., Kim, C.H., Bae, Y.K., Yeo, S.Y., Kim, S.H., Hong, S.K., Shin, J., Yoo, K.W., Hibi, M., Hirano, T., et al. (2000). Analysis of upstream elements in the HuC promoter leads to the establishment of transgenic zebrafish with fluorescent neurons. Dev. Biol. *227*, 279–293.

Peralta, M., González-Rosa, J.M., Marques, I.J., and Mercader, N. (2014). The epicardium in the embryonic and adult zebrafish. J. Dev. Biol. *2*, 101–116.

Peralta, M., Steed, E., Harlepp, S., González-Rosa, J.M., Monduc, F., Ariza-Cosano, A., Cortés, A., Rayón, T., Gómez-Skarmeta, J.L., Zapata, A., et al. (2013). Heartbeat-driven pericardiac fluid forces contribute to epicardium morphogenesis. Curr. Biol. *23*, 1726–1735.

Pérez-Pomares, J.M., Carmona, R., González-Iriarte, M., Atencia, G., Wessels, A., and Muñoz-Chápuli, R. (2002). Origin of coronary endothelial cells from epicardial mesothelium in avian embryos. Int. J. Dev. Biol. *46*, 1005–1013.

Pérez-Pomares, J.M., and de La Pompa, J.L. (2011). Signaling during epicardium and coronary vessel development. Circ. Res. *109*, 1429–1442.

Pérez-Pomares, J.M., Macías, D., García-Garrido, L., and Muñoz-Chápuli, R. (1997). Contribution of the primitive epicardium to the subepicardial mesenchyme in hamster and chick embryos. Dev. Dyn. *210*, 96–105.

Pérez-Pomares, J.M., Macías, D., García-Garrido, L., and Muñoz-Chápuli, R. (1998). The origin of the subepicardial mesenchyme in the avian embryo: an immunohistochemical and quail-chick chimera study. Dev. Biol. *200*, 57–68.

Pérez-Pomares, J.M., Phelps, A., Sedmerova, M., and Wessels, A. (2003). Epicardial-like cells on the distal arterial end of the cardiac outflow tract do not derive from the proepicardium but are derivatives of the cephalic pericardium. Dev. Dyn. *227*, 56–68.

Perner, B., Englert, C., and Bollig, F. (2007). The Wilms tumor genes wt1a and wt1b control different steps during formation of the zebrafish pronephros. Dev. Biol. *309*, 87–96.

Picelli, S., Björklund, Å.K., Faridani, O.R., Sagasser, S., Winberg, G., and Sandberg, R. (2013). Smart-seq2 for sensitive full-length transcriptome profiling in single cells. Nat. Methods *10*, 1096–1098.

Plavicki, J.S., Hofsteen, P., Yue, M.S., Lanham, K.A., Peterson, R.E., and Heideman, W. (2014). Multiple modes of proepicardial cell migration require heartbeat. BMC Dev. Biol. *14*, 18.

Qiu, X., Mao, Q., Tang, Y., Wang, L., Chawla, R., Pliner, H.A., and Trapnell, C. (2017). Reversed graph embedding resolves complex single-cell trajectories. Nat. Methods *14*, 979–982.

Robinson, J.T., Thorvaldsdóttir, H., Winckler, W., Guttman, M., Lander, E.S., Getz, G., and Mesirov, J.P. (2011). Integrative genomics viewer. Nat. Biotechnol. *29*, 24–26.

Robinson, M.D., Mccarthy, D.J., and Smyth, G.K. (2010). edgeR: a Bioconductor package for differential expression analysis of digital gene expression data. Bioinformatics *26*, 139–140.

Rodriguez-Meira, A., Buck, G., Clark, S.A., Povinelli, B.J., Alcolea, V., Louka, E., Mcgowan, S., Hamblin, A., Sousos, N., Barkas, N., et al. (2019). Unravelling intratumoral heterogeneity through high-sensitivity single-cell mutational analysis and parallel RNA sequencing. Mol. Cell *73*, 1292–1305.e8.

Ruiz-Villalba, A., Ziogas, A., Ehrbar, M., and Pérez-Pomares, J.M. (2013). Characterization of epicardial-derived cardiac interstitial cells: differentiation and mobilization of heart fibroblast progenitors. PLoS One *8*, e53694.

Sengbusch, J.K., He, W., Pinco, K.A., and Yang, J.T. (2002). Dual functions of [alpha]4[beta]1 integrin in epicardial development: initial migration and long-term attachment. J. Cell Biol. *157*, 873–882.

Serluca, F.C. (2008). Development of the proepicardial organ in the zebrafish. Dev. Biol. *315*, 18–27.

Simões, F.C., and Riley, P.R. (2018). The ontogeny, activation and function of the epicardium during heart development and regeneration. Development *145*, dev155994.

Snider, P., Standley, K.N., Wang, J., Azhar, M., Doetschman, T., and Conway, S.J. (2009). Origin of cardiac fibroblasts and the role of periostin. Circ. Res. *105*, 934–947.

Stevens, S.M., von Gise, A., Vandusen, N., Zhou, B., and Pu, W.T. (2016). Epicardium is required for cardiac seeding by yolk sac macrophages, precursors of resident macrophages of the adult heart. Dev. Biol. *413*, 153–159.

Swonger, J.M., Liu, J.S., Ivey, M.J., and Tallquist, M.D. (2016). Genetic tools for identifying and manipulating fibroblasts in the mouse. Differentiation *92*, 66–83.

Terriente, J., Gerety, S.S., Watanabe-Asaka, T., Gonzalez-Quevedo, R., and Wilkinson, D.G. (2012). Signalling from hindbrain boundaries regulates neuronal clustering that patterns neurogenesis. Development *139*, 2978–2987.

Trapnell, C., Cacchiarelli, D., Grimsby, J., Pokharel, P., Li, S., Morse, M., Lennon, N.J., Livak, K.J., Mikkelsen, T.S., and Rinn, J.L. (2014). The dynamics and regulators of cell fate decisions are revealed by pseudotemporal ordering of single cells. Nat. Biotechnol. *32*, 381–386.

Traver, D., Paw, B.H., Poss, K.D., Penberthy, W.T., Lin, S., and Zon, L.I. (2003). Transplantation and in vivo imaging of multilineage engraftment in zebrafish bloodless mutants. Nat. Immunol. *4*, 1238–1246.

Trinh, L.A., Chong-Morrison, V., Gavriouchkina, D., Hochgreb-Hägele, T., Senanayake, U., Fraser, S.E., and Sauka-Spengler, T. (2017). Biotagging of specific cell populations in zebrafish reveals gene regulatory logic encoded in the nuclear transcriptome. Cell Rep. *19*, 425–440.

Valentin, G., Haas, P., and Gilmour, D. (2007). The chemokine SDF1a coordinates tissue migration through the spatially restricted activation of Cxcr7 and Cxcr4b. Curr. Biol. *17*, 1026–1031.

von Gise, A., and Pu, W.T. (2012). Endocardial and epicardial epithelial to mesenchymal transitions in heart development and disease. Circ. Res. *110*, 1628–1645.

Wang, J., Cao, J., Dickson, A.L., and Poss, K.D. (2015). Epicardial regeneration is guided by cardiac outflow tract and Hedgehog signalling. Nature *522*, 226–230.

Wessels, A., van Den Hoff, M.J., Adamo, R.F., Phelps, A.L., Lockhart, M.M., Sauls, K., Briggs, L.E., Norris, R.A., van Wijk, B., Perez-Pomares, J.M., et al. (2012). Epicardially derived fibroblasts preferentially contribute to the parietal leaflets of the atrioventricular valves in the murine heart. Dev. Biol. *366*, 111–124.

Wickham, H. (2016). ggplot2: Elegant Graphics for Data Analysis (Springer-Verlag).

Wu, S.P., Dong, X.R., Regan, J.N., Su, C., and Majesky, M.W. (2013). Tbx18 regulates development of the epicardium and coronary vessels. Dev. Biol. *383*, 307–320.

Yamaguchi, Y., Cavallero, S., Patterson, M., Shen, H., Xu, J., Kumar, S.R., and Sucov, H.M. (2015). Adipogenesis and epicardial adipose tissue: a novel fate of the epicardium induced by mesenchymal transformation and PPARγ activation. Proc. Natl. Acad. Sci. USA *112*, 2070–2075.

Yang, J.T., Rayburn, H., and Hynes, R.O. (1995). Cell adhesion events mediated by alpha 4 integrins are essential in placental and cardiac development. Development *121*, 549–560.

Yelon, D., Horne, S.A., and Stainier, D.Y. (1999). Restricted expression of cardiac myosin genes reveals regulated aspects of heart tube assembly in zebrafish. Dev. Biol. *214*, 23–37.

Zacharias, D.A., Violin, J.D., Newton, A.C., and Tsien, R.Y. (2002). Partitioning of lipid-modified monomeric GFPs into membrane microdomains of live cells. Science *296*, 913–916.

Zhou, B., Ma, Q., Rajagopal, S., Wu, S.M., Domian, I., Rivera-Feliciano, J., Jiang, D., von Gise, A., Ikeda, S., Chien, K.R., and Pu, W.T. (2008). Epicardial progenitors contribute to the cardiomyocyte lineage in the developing heart. Nature *454*, 109–113.

Zhou, B., von Gise, A., Ma, Q., Hu, Y.W., and Pu, W.T. (2010). Genetic fate mapping demonstrates contribution of epicardium-derived cells to the annulus fibrosis of the mammalian heart. Dev. Biol. *338*, 251–261.

Zhou, Y., Cashman, T.J., Nevis, K.R., Obregon, P., Carney, S.A., Liu, Y., Gu, A., Mosimann, C., Sondalle, S., Peterson, R.E., et al. (2011). Latent TGF-β binding protein 3 identifies a second heart field in zebrafish. Nature *474*, 645–648.

STAR★METHODS

KEY RESOURCES TABLE

REAGENT or RESOURCE	SOURCE	IDENTIFIER
Antibodies		
chicken polyclonal anti-GFP	abcam	Cat# ab13970, RRID: AB_300798
Living Colors® DsRed Polyclonal Antibody rabbit	Clontech	Cat# 632496, RRID: AB_10013483
mouse monoclonal anti-Dendra2	Origene	Cat# TA180094, clone OTI1G6, RRID: AB_2622288
mouse monoclonal anti-Mlck	Sigma-Aldrich	Cat# M7905, RRID: AB_477243
rabbit polyclonal anti-cxcr4b	Genetex	Cat# GTX132244; RRID: AB_2827420
Chemicals, Peptides, and Recombinant Proteins		
RNase Inhibitor	Clontech	Cat# 2313A
SMARTScribe reverse transcriptase	Clontech	Cat# 639537
SeqAMP DNA polymerase	Clontech	Cat# 638509
Protease	Qiagen	Cat# 19155
Herculase II Fusion DNA Polymerase	Agilent	Cat# 600675
Critical Commercial Assays		
Quant-iT PicoGreen dsDNA Assay	Thermo Fisher Scientific	Cat# P11496
Nextera XT library preparation kit	Illumina	Cat# FC-131-1024
RNAqueous Micro Total RNA isolation kit	Ambion	Cat# AM1931
NextSeq ® 500/550 Mid Output Kit v2.5 (150 cycles)	Illumina	Cat# 20024904
NextSeq ® 500/550 High Output Kit v2.5 (75 cycles)	Illumina	Cat# 20024906
See STAR Methods section for Hybridization Chain Reaction (HCR) v3.0	Molecular Instruments	N/A
Click-iT™ EdU Alexa Fluor™ 647 Imaging Kit	Thermo Fisher Scientific	Cat# C10340
InFusion HD Cloning kit	Clontech	Cat# 638910
Deposited Data		
Raw and analysed single-cell RNA-seq data	This paper	GEO: GSE121750
Experimental Models: Organisms/Strains		
TgBAC(tcf21:myr-tdTomato)^{ox181}	Riley and Sauka-Spengler laboratories	ox181
TgBAC(tcf21:H2B-Dendra2)^{ox182}	Riley and Sauka-Spengler laboratories	ox182
TgBAC(tcf21:myr-eGFP)^{ox183}	Riley and Sauka-Spengler laboratories	ox183
TgBAC(tbx18:myr-eGFP)^{ox184}	Riley and Sauka-Spengler laboratories	ox184
TgBAC(tbx18:myr-Citrine)^{ox185}	Riley and Sauka-Spengler laboratories	ox185
TgBAC(wt1b:H2B-Dendra2)^{ox186}	Riley and Sauka-Spengler laboratories	ox186
TgBAC(tcf21:myr-tdTomato;tbx18:myr-eGFP;wt1b:H2B-Dendra2)^{ox187}	Riley and Sauka-Spengler laboratories	ox187
TgBAC(wt1b:Cre-2A-mCherry)^{ox142}	Riley and Sauka-Spengler laboratories	ox142
Tg(tgm2b^{38ins};tcf21:H2B-Dendra2;tbx18:myr-Citrine)^{ox188}	Riley and Sauka-Spengler laboratories	ox188
Tg(tgm2b^{7del1sub};tcf21:H2B-Dendra2;tbx18:myr-Citrine)^{ox189}	Riley and Sauka-Spengler laboratories	ox189
Tg(cxcl12a^{15del}; ptprc/CD45:DsRed; tcf21:myr-eGFP)^{ox190}	Riley and Sauka-Spengler laboratories	ox190
TgBAC(tcf21:DsRed2)^{pd37}	Kikuchi et al., 2011	ZFIN: ZDB-TGCONSTRCT-110818-7
TgBAC(tbx18:DsRed2)^{pd22}	Kikuchi et al., 2011	ZFIN: ZDB-TGCONSTRCT-110818-6

(*Continued on next page*)

Continued

REAGENT or RESOURCE	SOURCE	IDENTIFIER
TgBAC(cryaa:EGFP,tcf21:Cre-ERT2)^{pd42}	Kikuchi et al., 2011	ZFIN: ZDB-TGCONSTRCT-110818-8
Tg(wt1b:eGFP)^{li1}	Perner et al., 2007	ZFIN: ZDB-TGCONSTRCT-071127-1
Tg(myl7:eGFP)^{f1}	Huang et al., 2003	ZFIN: ZDB-TGCONSTRCT-070117-164
Tg(kdrl:GFP)^{s843}	Beis et al., 2005	ZFIN: ZDB-TGCONSTRCT-070529-1
Tg(gata1a:DsRed)	Traver et al., 2003	ZFIN: ZDB-TGCONSTRCT-070117-38
Tg(ptprc/CD45:DsRed)^{sd3}	Bertrand et al., 2008	ZFIN: ZDB-TGCONSTRCT-081120-3
Tg(–3.5ubb:loxP-EGFP-loxP-mCherry)	Mosimann et al., 2011	ZFIN: ZDB-TGCONSTRCT-110124-1
Tg(ubb:loxPAmCyanSTOPloxPZsYellow)	Zhou et al., 2011	ZFIN: ZDB-TGCONSTRCT-111115-6
Oligonucleotides		
Smart-seq2 LNA-TSO: AAGCAGTGGTATCAACGCAGAGTACATrGrG+G	Picelli et al., 2013	N/A
Smart-seq2 OligodT-ISPCR primer: aagcagtggtatcaacgcagagtacttttttttttttttttttttttttttttttvn	Picelli et al., 2013	N/A
Smart-seq2 ISPCR primer: AAGCAGTGGTATCAACGCAGAGT	Picelli et al., 2013	N/A
See Table S1 for BAC recombineering primers	This paper	N/A
See Table S2 for sgRNA cloning primers	This paper	N/A
See Table S3 for sgRNA/Cas9 nuclease activity test primers	This paper	N/A
See Table S4 for *sema3fb* and *cxcl12a* cDNA/ genomic DNA primers	This paper	N/A
Recombinant DNA		
pGEM-BirA-2A-H2B-Dendra2-SV40pA-FKF	This paper	Addgene Cat# 119864
pGEM-myr-Citrine-SV40pA-FKF	This paper	Addgene Cat#119865
pGEM-myr-eGFP-SV40pA-FKF	This paper	Addgene Cat#119866
pGEM-myr-tdTomato-SV40pA-FKF	This paper	Addgene Cat#119867
pGEM-NLS-Cre-2A-mCherry-FKF	This paper	Addgene Cat#119868
BAC clone DKEYP-79F12	https://www.sourcebioscience.com	DKEYP-79F12
BAC clone DKEYP-117G5	https://www.sourcebioscience.com	DKEYP-117G5
BAC clone CH73-157N22	https://www.sourcebioscience.com	CH73-157N22
Software and Algorithms		
STAR v2.4.2a	Dobin et al. (2013)	https://github.com/alexdobin/STAR
samtools v1.3	(Li et al., 2009)	http://htslib.org/
FeatureCounts v1.6.2	Liao et al. (2014)	http://bioinf.wehi.edu.au/featureCounts
velocyto v0.17.13	La Manno et al., 2018	http://velocyto.org/
R v3.4.3	R Core Team	https://www.r-project.org/
scater v1.6.3	McCarthy et al., 2017	https://bioconductor.org/packages/release/bioc/html/scater.html
scde v1.99.4	Fan et al. (2016)	http://hms-dbmi.github.io/scde/index.html
edgeR v3.20.9	Robinson et al., 2010	https://bioconductor.org/packages/release/bioc/html/edgeR.html
velocyto.R v0.5	La Manno et al., 2018	https://github.com/velocyto-team/velocyto.R
monocle v2.6.3	Qiu et al., 2017	https://github.com/cole-trapnell-lab/monocle-release
topGO v2.30.1	(Alexa and Rahnenfuehrer, 2019)	http://bioconductor.org/packages/release/bioc/html/topGO.html
Rtsne v0.13	Krijthe, 2015	https://github.com/jkrijthe/Rtsne
ggplot2 v 2.2.1	(Wickham, 2016)	https://ggplot2.tidyverse.org/
IGV v2.5.0	(Robinson et al., 2011)	https://software.broadinstitute.org/software/igv/

LEAD CONTACT AND MATERIALS AVAILABILITY

Further information and requests for resources and reagents should be directed to and will be fulfilled by the Lead Contact, Paul Riley (paul.riley@dpag.ox.ac.uk). Requests for zebrafish transgenic lines should be directed to Tatjana Sauka-Spengler (tatjana.sauka-spengler@imm.ox.ac.uk). All plasmids generated in this study are readily available from Addgene (www.addgene.org/Tatjana_Sauka-Spengler/ pGEM-BirA-2A-H2B-Dendra2-SV40pA-FKF, Cat#119864; pGEM-myr-Citrine-SV40pA-FKF, Cat#119865; pGEM-myr-eGFP-SV40pA-FKF, Cat#119866; pGEM-myr-tdTomato-SV40pA-FKF Cat#119867; pGEM-NLS-Cre-2A-mCherry-FKF, Cat#119868).

EXPERIMENTAL MODEL AND SUBJECT DETAILS

For this study, both females and males of transgenic and wildtype zebrafish strains were used. Animals used for breeding were between 3 and 24 months old. Zebrafish embryos/larvae that were used for experiments were raised to an age of up to 7 days post fertilisation (dpf). Larvae were euthanised and analysed shortly before reaching an age of 5dpf (free-feeding) during all experiments for which the experimental timepoint is stated as "5dpf" or "120hpf" in text or figures. Fish were kept at a 14 hours light, 10 hours dark cycle and fed four times a day. All animal experiments were performed under a Home Office Licence according to the Animals Scientific Procedures Act 1986, UK, and approved by the local ethics committee.

Zebrafish Lines

Published transgenic reporter lines used in this study were: *TgBAC(tcf21:DsRed2)^{pd37}* (Kikuchi et al., 2011), *TgBAC(tbx18:DsRed2)^{pd22}* (Kikuchi et al., 2011), *TgBAC(cryaa:EGFP,tcf21:Cre-ERT2)^{pd42}* (Kikuchi et al., 2011), *Tg(wt1b:eGFP)^{li1}* (Perner et al., 2007), *Tg(myl7:eGFP)^{f1}* (Huang et al., 2003), *Tg(kdrl:GFP)^{s843}* (Beis et al., 2005), *Tg(gata1a:DsRed)* (Traver et al., 2003), *Tg(ptprc/CD45:DsRed)^{sd3}* (Bertrand et al., 2008), *Tg(–3.5ubb:loxP-EGFP-loxP-mCherry)* [*Tg(ubi:Switch)*] (Mosimann et al., 2011) and *Tg(ubb:loxPAmCyanSTOPloxPZsYellow)* [*Tg(ubi:CSY)*] (Zhou et al., 2011). *Tg(tcf21:DsRed2; myl7:eGFP)* and *Tg(kdrl:GFP; gata1a:DsRed)* double transgenic lines were generated by natural mating.

METHOD DETAILS

Generation of Transgenic Zebrafish Lines

To generate TgBAC(tcf21:myr-tdTomato)^{ox181}, TgBAC(tcf21:H2B-Dendra2)^{ox182}, TgBAC(tcf21:myr-eGFP)^{ox183}, TgBAC(tbx18:myr-eGFP)^{ox184}, TgBAC(tbx18:myr-Citrine)^{ox185}, TgBAC(wt1b:H2B-Dendra2)^{ox186} and TgBAC(wt1b:Cre-2A-mCherry)^{ox142} we used a BAC recombineering approach (Bussmann and Schulte-Merker, 2011). Dendra2, tdTomato, eGFP and Citrine fluorophore sequences were linked to 5' histone 2b (H2B) or 2x myristoylation (myr) sequences (Dempsey et al., 2012; Zacharias et al., 2002). pGEM H2B-Dendra2-FRT-Kan-FRT, pGEM myr-tdTomato-FRT-Kan-FRT, pGEM myr-eGFP-FRT-Kan-FRT, pGEM myr-Citrine-FRT-Kan-FRT and pGEM Cre-2A-mCherry-FRT-Kan-FRT recombination donor constructs were generated by amplifying H2B-Dendra2, myr-tdTomato, myr-eGFP, myr-Citrine and Cre-2A-mCherry cassettes using Herculase II fusion DNA polymerase (Agilent Technologies) and cloning them into the donor plasmid (#89890, Addgene) using InFusion (InFusion HD Cloning kit, Clontech). BACs used were DKEYP 79F12 (tcf21), DKEYP 117G5 (tbx18) and CH73 157N22 (wt1b). Donor constructs were inserted into the first coding exon of tcf21, tbx18 or wt1b BACs via bacterial homologous recombination as previously described (Trinh et al., 2017). Primers used are listed in Table S1. The corresponding constructs used for amplification of donor fragments flanked with recombinant arms are available from Addgene (#119864 - #119868). BACs were injected into one-cell zygotes and integrated into the genome via Tol2-mediated recombination. TgBAC(tcf21:myr-tdTomato;tbx18:myr-eGFP;wt1b:H2B-Dendra2)^{ox187} was generated by two consecutive rounds of breeding from TgBAC(tcf21:myr-tdTomato)^{ox181}, TgBAC(wt1b:H2B-Dendra2)^{ox186} and TgBAC(tbx18:myr-eGFP)^{ox184}. Images of euthanised larvae were obtained using a LSM780 confocal microscope (ZEISS) and a 20x objective.

Larval Heart Isolation, Dissociation and FAC-Sorting

Larvae were euthanised using tricaine methanesulfonate (MS-222) and larval hearts were isolated following a published protocol (Burns and MacRae, 2006), using a 21-gauge needle for disruption. This procedure recovered around 50% of the larval hearts. Hearts were dissociated using 15mg/ml collagenase (C8176, Sigma Aldrich) in 0.05% trypsin solution at 30°C for 14mins. Single cells were sorted on a BD FACS-Aria Fusion into Smart-seq2 lysis buffer dispensed in 96 well plates. 7-AAD cell viability dye was used to exclude non-viable cells during FACS. 288 fluorescent cells were purified from *TgBAC(tcf21:H2B-Dendra2)^{ox182}* (300 hearts), 96 fluorescent cells from *TgBAC(tbx18:myr-eGFP)^{ox184}* (180 hearts) and 96 fluorescent cells from *TgBAC(wt1b:H2B-Dendra2)^{ox186}* (130 hearts). Additionally, 31 GFP positive cells were collected from *Tg(myl7:eGFP)^{f1}* (40 hearts), 40 GFP positive cells and 20 DsRed fluorescent cells from *Tg(kdrl:GFP; gata1a:DsRed)* (65 hearts), 96 non-fluorescent cells from *Tg(tcf21:DsRed2; myl7:eGFP)* x *Tg(kdrl:GFP; gata1a:DsRed)* (100 ventricles) and 96 cells from 50 wildtype hearts. Cells from *TgBAC(tcf21:H2B-Dendra2)^{ox182}* were sorted in four separate FACS sessions, GFP positive and DsRed positive cells from *Tg(kdrl:GFP; gata1a:DsRed)* and GFP positive cells from *Tg(myl7:eGFP)^{f1}* were sorted in one session, cells in all other conditions were sorted separately in one session each. Cells sorted in separate FACS sessions were processed separately during cDNA synthesis, cells sorted in one session

were processed as one batch. Three empty wells were processed within the *kdrl*:GFP, *gata1a*:DsRed batch. During library preparation and sequencing, cDNA samples from *tbx18*:myr-eGFP, *wt1b*:H2B-Dendra2, *kdrl*:GFP, *gata1a*:DsRed and *myl7*:eGFP conditions were scrambled and processed as two new batches, *tcf21*:H2B-Dendra2,*myl7*:eGFP,*kdrl*:GFP,*gata1a*:DsRed quadruple negative and wildtype conditions were processed in one batch, all other conditions were processed separately.

During *sema3fb* knockout experiments, 241 Citrine positive single cells were purified from 300 *TgBAC(tbx18:myr-Citrine)*^{ox185} hearts in three batches and sorted into 96-well plates containing lysis buffer with protease (Qiagen). cDNA was synthesised separately for each batch. Batches were combined during library preparation and sequencing.

Single Cell Library Preparation and Sequencing

Single cells were processed following the Smart-seq2 protocol (Picelli et al., 2013) to reverse transcribe poly-adenylated RNA. cDNA was then amplified with 20 PCR cycles. In two of the batches of cells sorted from *TgBAC(tcf21:H2B-Dendra2)*^{ox182}, all cDNA samples were taken further into library preparation and sequencing. However, only a low percentage of these samples passed the quality control after sequencing (Figure S2F). Therefore, in all other batches the amount of synthesized cDNA was quantified using the Quant-iT PicoGreen dsDNA Assay (ThermoFisher). cDNA quality of 11 samples sampling the whole range of the obtained PicoGreen results was tested on an Agilent 2100 Bioanalyzer (Agilent Technologies). The Bioanalyzer results were used to determine a quality cut-off PicoGreen value. Only cDNA samples with a PicoGreen value above the cut-off were taken further into library preparation. Final sequencing libraries were prepared using the Nextera XT DNA Library Preparation Kit (FC-131-1024, Illumina) and 75bp paired-end sequencing reads were generated on a NextSeq500 platform (Illumina). The number of sequenced cells for each condition can be found in Figure S2.

KO *sema3fb* Single Cell Library Preparation and Sequencing

Single cells isolated from hearts of euthanised 5dpf *sema3fb* knockout larvae were processed adapting the TARGET-seq protocol (Rodriguez-Meira et al., 2019). Similar to TARGET-seq, single cells were subjected to a mild protease digestion to release genomic DNA. In addition to template-switching and oligo-dT primers, primers specific to *sema3fb* mRNA were used to enforce reverse transcription of *sema3fb* during first-strand synthesis. Primers specific for *sema3fb* cDNA were added during cDNA amplification. Additionally, genomic DNA primers binding intronic sequences surrounding the target sites of *sema3fb* sgRNA1 (exon 2) and sgRNA4 (exon 10) were added into the same PCR mix. Single cell cDNA was amplified using 24 PCR cycles. Primer specificity was validated against zebrafish RefSeq mRNA and genomic DNA assemblies in PrimerBlast. Final sequencing libraries were prepared using the Nextera XT DNA Library Preparation Kit (Illumina) and 130 libraries were sequenced on a NextSeq500 platform (Illumina), generating 75bp paired-end sequencing reads. A subset of 40 samples was re-sequenced for increased coverage of the *sema3fb* locus, generating 75bp single-read sequencing reads. Primer sequences can be found in Table S4.

KO *cxcl12a* Single Embryo Library Preparation and Sequencing

Zygotes were injected with two sgRNAs targeting AmCyan (control, n=3) or sgRNA1 and sgRNA3 targeting *cxcl12a* (KO *cxcl12a*, n=6), single larvae were euthanised using MS-222 shortly before 5dpf and RNA was extracted using the RNaqueous Micro Kit (Ambion). Approximately 50ng of purified RNA was processed with SMARTScribe reverse transcriptase (Clontech), using 1.5μM *cxcl12a* specific exonic reverse primer and 5μM Smart-seq2 template-switching oligo. cDNA was amplified using SeqAmp polymerase (Clontech), 0.33 μM Smart-seq2 ISPCR primers and 6 PCR cycles. Final sequencing libraries were prepared using the Nextera XT DNA Library Preparation Kit (Illumina) and libraries were sequenced on a NextSeq500 platform (Illumina), generating 75bp paired-end sequencing reads. All samples were re-sequenced for increased coverage, generating 75bp single-read sequencing reads. The *cxcl12a* primer sequence can be found in Table S4.

Hybridisation Chain Reaction

Larvae were euthanised using MS-222 and fixed in 4% paraformaldehyde (PFA) overnight at 4°C. Subsequently, larvae were stored at -20°C in methanol. Hybridisation chain reaction (HCR) v3.0 (Choi et al., 2018) was performed following a protocol by Choi et al. Briefly, larvae were permeabilized using 30μg/ml proteinase K for 45 minutes at room temperature, post-fixed in 4% PFA and incubated overnight at 37°C in 30% probe hybridisation buffer containing 2pmol of each probe mixture. Excess probes were washed off with 30% probe wash buffer at 37°C and 5xSSCT at room temperature and larvae were incubated overnight at room temperature in amplification buffer containing 15pmol of each fluorescently labelled hairpin. Following HCR, larvae were incubated with Hoechst reagent (1:1000, 5xSSCT) for 30 minutes at room temperature. Probe sequences were designed by the manufacturer. Probe sets used were: dr_tcf21-B1, dr_tbx18-B2, dr_wt1b-B3, dr_myl7-B5, dr_adma-B3, dr_jam2b-B4, dr_elnb-B4, dr_cldn11a-B2, dr_tgm2b-B4, dr_sema3fb-B5, dr_nrp1a-B4, dr_nrp2a-B3, dr_cxcl12a-B3, dr_cxcr4b-B5, dr_ptprc-B4, EGFP-B4, Dendra2-B5, tdTomato-B5, mCherry-B1. During BAC reporter line validation, HCR was performed using probes specifically targeting exon 1 of *tcf21*, *tbx18* or *wt1b* (dr_tcf21_exon1-B1, dr_tbx18_exon1-B2, dr_wt1b_exon1-B3), which was absent from the BAC reporter constructs. Images were obtained using a LSM780 confocal microscope (ZEISS) and a 40x objective. Contrast and brightness were adjusted separately for each colour channel.

Immunocytochemistry

Larvae were euthanised using MS-222 and fixed in 4% PFA for 45 minutes at room temperature. After washing, larvae were blocked using 5% goat serum (PBS, 0.5% Triton, 2% DMSO) for 1 hour at room temperature. Primary antibodies used were: chicken anti-GFP (ab13970, abcam, 1:500), rabbit anti-DsRed (632496, Clontech, 1:500), mouse anti-Dendra2 (TA180094, clone OTI1G6, Origene, 1:500), mouse anti-Mlck (M7905, Sigma-Aldrich, 1:500) and rabbit anti-cxcr4b (GTX132244, Genetex, 1:300), added overnight at 4°C. Secondary antibodies and Hoechst reagent were used at 1:1000 dilution and added for 2 hours at room temperature. Images were obtained using a LSM780 confocal microscope (ZEISS) and a 40x objective, or a MVX10 fluorescence stereo microscope (Olympus). Contrast and brightness were adjusted separately for each colour channel.

EdU Staining

Larvae were incubated in embryo medium containing 1mM EdU and 1% DMSO. During *sema3fb* knockout experiments, larvae were incubated for 18 hours, from 102hpf to shortly before 120hpf. During *tgm2b* knockout experiments, larvae were incubated for 11 hours, from 66hpf until 77hpf. KO *sema3fb* larvae were stained by immunocytochemistry against GFP, followed by detection of EdU using the Click-iT Alexa Fluor 647nm kit (ThermoFisher) for 2 hours at room temperature. KO *tgm2b* larvae were immuno-stained against Dendra2, followed by EdU detection.

Lineage Tracing

4-hydroxytamoxifen (4-OHT) was added to the embryo medium at a final concentration of 10μM, from a 1mM stock solution made in 100% ethanol. Embryos were incubated with 4-OHT from 10 hours-post-fertilisation (hpf) or 43hpf. For embryos incubated with 4-OHT from 10hpf, the 4-OHT containing medium was renewed at 2dpf and at 4dpf. For embryos incubated from 43hpf, the 4-OHT containing medium was replaced at 4dpf. Embryos were grown until shortly before 5dpf.

Transient Cas9-Mediated Gene Knockout

sgRNAs against the coding region of the target gene were designed to have a high Cas9-mediated nuclease cutting efficiency, a low number of genomic off-target sites and a target site proximal to the recognition site of a restriction enzyme. sgRNA primers (see Table S2) were annealed and the product inserted via Golden Gate cloning into a U6a mini-vector containing a tracrRNA backbone and Ds transposon sequences (Addgene #119069). sgRNA vectors were first injected individually to test their Cas9-mediated nuclease cutting efficiency. Typically, 30pg of vector was injected into one-cell stage zygotes, together with 10pg Ac mRNA and 160pg Cas9 mRNA. For actual gene editing experiments, two sgRNA vectors were co-injected at an amount of 30-38pg each. Control embryos were injected with two sgRNA vectors targeting mCherry (KO *tgm2b*, *sema3fb*) or AmCyan (KO *cxcl12a*).

Cas9 Activity Efficiency Test

One cell stage zygotes were injected with a single sgRNA vector, Ac mRNA and Cas9 mRNA. Genomic DNA from single euthanised embryos was isolated at 1dpf and the genomic region surrounding the sgRNA target site (200-400bp) was amplified via PCR (see Table S3 for primers used). Half the volume of each PCR product was digested with a restriction enzyme that had a recognition site overlapping or adjacent to the sgRNA target site.

Generation of Stable Mutant Zebrafish Lines

We injected *tgm2b* sgRNA2 vector, Ac mRNA and Cas9 mRNA into one cell stage *TgBAC(tcf21:H2B-Dendra2)*^{ox182} x *TgBAC(tbx18:myr-Citrine)*^{ox185} zygotes, reared fluorescent fish to adulthood and screened for germline transmission of editing events to generate *tgm2b*$^{38ins/38ins}$ *mutant in the Tg(tcf21:H2B-Dendra2;tbx18:myr-Citrine)* background (ox188) and *tgm2b*$^{7del1sub/7del1sub}$ *mutant in Tg(tcf21:H2B-Dendra2;tbx18:myr-Citrine)* background (ox189). Similarly, we injected *cxcl12a* sgRNA1 vector into *Tg(ptprc/CD45:DsRed)*^{sd3} x *TgBAC(tcf21:myr-eGFP)*^{ox183} zygotes to generate *cxcl12a*$^{15del/15del}$ in *Tg(ptprc/CD45:DsRed; tcf21:myr-eGFP)* background (ox190). To identify mutants, embryos were fin-clipped and extracted genomic DNA was PCR amplified using the primers:

*tgm2b*_sgRNA2_for GGGAGGAAGCGTTCAGATACTA and

*tgm2b*_sgRNA2_rev ATACCGCACTGGCAGACAC (wildtype PCR product size 61bp), or *cxcl12a*_sgRNA1_for GCAAACATG GATCTCAAAGTGA and

*cxcl12a*_sgRNA1_rev TTAACTTACCGTTGGAAATCGG (wildtype PCR product size 82bp). The sizes of the PCR products were then determined using gel electrophoresis. Mutant genotypes were characterised via Sanger sequencing.

Bioinformatic Processing

Single Cell RNA Sequencing Data Analysis

Transcriptomic data was mapped to the zebrafish reference genome (GRCz10-91) using the STAR gapped aligner (Dobin et al., 2013). Duplicate reads were removed and reads were summarized using featureCounts (Liao et al., 2014). FeatureCounts files containing data from different sequencing batches were subsequently concatenated. Quality control of the single cell read counts was done using the Scater R package (McCarthy et al., 2017). Libraries that had a size more than 3 median absolute deviations (MADs) below the median of the whole dataset were excluded from the analysis. Furthermore, libraries were excluded from the analysis if the number of expressed genes was more than 3 MADs below the median of the whole dataset, or if the percentage of counts

representing ERCC spike-in features was more than 3 MADs above the median of the whole dataset. Genes with an average expression across all cells of below 0.1 counts as well as mitochondrial genes were excluded from analysis. Quality control plots can be found in Figure S2. The cleaned dataset was further processed using Pagoda routines in the scde package (Fan et al., 2016; Kharchenko et al., 2014). Genes annotated to cell cycle related gene ontology terms were excluded from the dataset. Gene enrichment in the single cell clusters was analysed with scde. Counts were transformed into FPKM expression values with the rpkm() command in the edgeR package (Robinson et al., 2010) and heatmaps drawn using pheatmap. The Rtsne package (Krijthe, 2015) was used to plot t-SNE representations of the dataset that were based on 2376 highly variable genes. Gene ontology term analysis was performed with the R topGO package (Alexa and Rahnenfuehrer, 2019), using enriched genes with cZ>2. RNA velocity was analysed using the Velocyto R package (La Manno et al., 2018) and pseudotime analysis was performed using the Monocle 2 package (Qiu et al., 2017).

KO sema3fb *Single Cell RNA Sequencing Data Analysis*

To identify single cell clusters, sequencing data from *sema3fb* knockout samples was processed using the Pagoda pipeline as described above. FPKM values were computed using the rpkm() function in edgeR. Raw counts for *sema3fb* were generated using featureCounts and a custom gene annotation in which exons targeted by *sema3fb* sgRNAs and amplified from genomic DNA (exons 2 and 10) were absent. Raw counts were then normalised to sample library size; no *sema3fb* FPKM values were determined since effective *sema3fb* transcript lengths could not reliably be computed for each single cell sample. To analyse genome editing, STAR mapped sequencing reads (without duplicates) were inspected using the Integrative Genomics Viewer (Robinson et al., 2011). For re-sequenced samples, the two BAM files were merged using samtools (Li et al., 2009). 14 samples containing reads covering at least one of the sgRNA target sites (sgRNA1: chr11:35307703, sgRNA4:chr11:35213000) were analysed in further detail. For these samples, sequences of reads spanning the sgRNA target sites were compared to the genomic reference sequence and to reads of wildtype *sema3fb* positive cells from the original scRNASeq dataset. Samples with read sequences that differed from these reference sequences were classified as genome-edited. Editing types detected were grouped into insertions/deletions (indels), basepair substitutions and large deletions over 10kb in length. The effect of genome editing on the sema3fb protein open reading frame (ORF) was analysed using SnapGene Viewer (SnapGene) and the lengths of edited sema3fb ORFs were normalised to wildtype sema3fb length (791aa). IGV was used to generate Sashimi plots showing splicing of *sema3fb*. In 9 samples that contained spliced reads for more than one *sema3fb* splice junction, the number of reads indicating aberrant *sema3fb* splicing was normalised against the total number of *sema3fb* splicing reads and plotted. 12 non *sema3fb* knockout *sema3fb* expressing single cell transcriptome samples were analysed similarly.

KO cxcl12a *RNA Sequencing Data Analysis*

Sequencing reads were mapped with STAR to GRCz10-91, duplicate reads removed and the *cxcl12a* locus analysed using IGV. Read sequences at the sgRNA target sites (chr13:30674045 sgRNA1, chr13:30676177 sgRNA3) were compared to the reference genome. The numbers of reads that contained indels or substitutions or that indicated aberrant splicing from exon 1 to exon 3 were normalised against the total number of reads spanning the respective sgRNA target site. Sashimi plots were generated with IGV and the number of reads that indicated aberrant splicing from exon 1 to exon 3 was normalised against the total number of reads splicing from exon 1. The cxcl12a ORFs resulting from genome editing were analysed in SnapGene Viewer and their lengths were normalised against the wildtype cxcl12a length (99aa). FPKM values were computed manually. To determine the effective transcript length (as produced by Smart-seq2 template-switching oligo and *cxcl12a* cDNA reverse primer) for each sample, the amount of *cxcl12a* transcripts not containing exon 2 (length 1224bp) was weighted against the amount of wildtype transcript (length 1342bp) using the previously computed ratio of aberrant splicing.

QUANTIFICATION AND STATISTICAL ANALYSIS

Statistical details of experiments can be found in the figures, figure legends and main text, including p-values and numbers of animals analysed. Statistical analyses were performed in R. Plots were generated with ggplot2 (Wickham, 2016) in R. For the scRNA-seq GO-term enrichment analysis, significance was calculated using Fisher's exact test with Bonferroni correction for multiple testing. For data shown in Figures 4–6, significance was calculated using Welch's t-test. Some plots include box and whiskers plots (in the style of Tukey), indicating median and first/third quartiles.

DATA AND CODE AVAILABILITY

Raw and processed sequencing data generated in this study was submitted to GEO (accession number GSE121750).

Developmental Cell, Volume *52*

Supplemental Information

Functional Heterogeneity within the Developing Zebrafish Epicardium

Michael Weinberger, Filipa C. Simões, Roger Patient, Tatjana Sauka-Spengler, and Paul R. Riley

Figure S1.

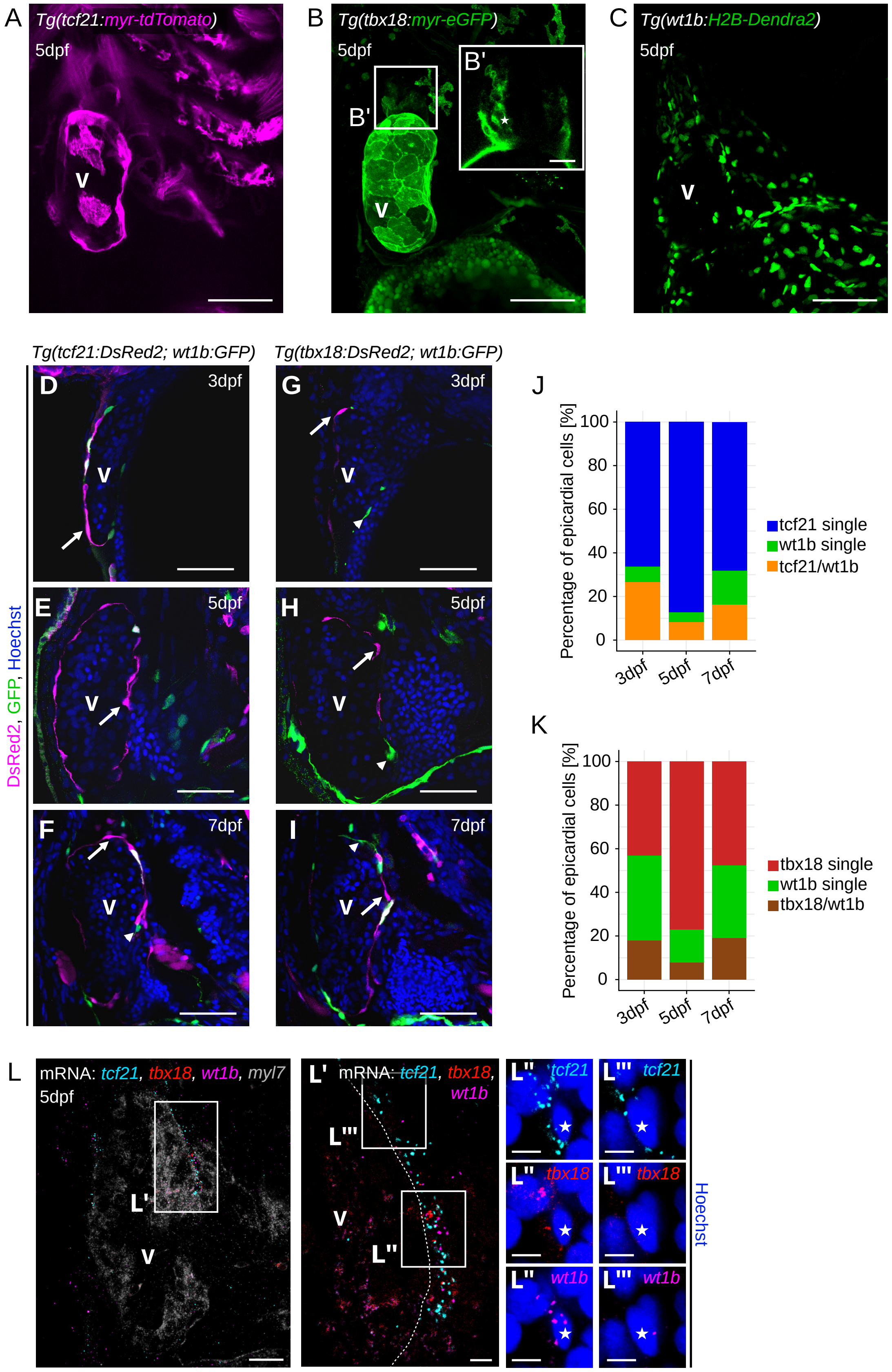
A
Tg(tcf21:myr-tdTomato)
5dpf
V
B
Tg(tbx18:myr-eGFP)
5dpf
B'
V
C
Tg(wt1b:H2B-Dendra2)
5dpf
V
Tg(tcf21:DsRed2; wt1b:GFP)
Tg(tbx18:DsRed2; wt1b:GFP)
DsRed2, GFP, Hoechst
D 3dpf
E 5dpf
F 7dpf
G 3dpf
H 5dpf
I 7dpf
J
Percentage of epicardial cells [%]
0
20
40
60
80
100
3dpf
5dpf
7dpf
tcf21 single
wt1b single
tcf21/wt1b
K
Percentage of epicardial cells [%]
tbx18 single
wt1b single
tbx18/wt1b
L
mRNA: tcf21, tbx18, wt1b, myl7
5dpf
L'
mRNA: tcf21, tbx18, wt1b
L''
L'''
tcf21
tbx18
wt1b
Hoechst

Figure S1. Heterogeneous expression of *tcf21*:DsRed2, *tbx18*:DsRed2 and *wt1b*:GFP in the developing zebrafish epicardium. Related to Figure 1.

(A-C) Projections of *TgBAC(tcf21:myr-tdTomato)* (A), *TgBAC(tbx18:myr-eGFP)* (B) and *TgBAC(wt1b:H2B-Dendra2)* (C) hearts at 5dpf. (B') Magnified single optical section of the boxed area in B showing the presence of *tbx18*:myr-eGFP$^+$ cells within the BA (asterisk). (D-F) Projections of the heart region in *tcf21*:DsRed2$^+$, *wt1b*:GFP$^+$ larvae at 3dpf (D), 5dpf (E) and 7dpf (F). Many epicardial cells are DsRed2$^+$, GFP$^-$ (arrows), at 7dpf some epicardial cells are DsRed2$^-$, GFP$^+$ (arrowhead). (G-I) Projections of the heart region in *tbx18*:DsRed2$^+$, *wt1b*:GFP$^+$ larvae at 3dpf (G), 5dpf (H) and 7dpf (I). Many epicardial cells are DsRed2$^+$, GFP$^-$ (arrows) and DsRed2$^-$, GFP$^+$ (arrowheads). (J) Relative quantification of the epicardial combinations of *tcf21*:DsRed2 and *wt1b*:GFP. 3dpf n=6, 5dpf n=9, 7dpf n=3. (K) Relative quantification of the epicardial combinations of *tbx18*:DsRed2 and *wt1b*:GFP. 3dpf n=6, 5dpf n=10, 7dpf n=6. (L) Visualisation of *tcf21* (cyan), *tbx18* (red) and *wt1b* (magenta) and *myl7* (grey) mRNA at 5dpf. (L'-L''') Cell nuclei (asterisks) in the epicardial region in close proximity to *tcf21*, *tbx18* and *wt1b* (L''), as well as in close proximity to *tcf21* and *wt1b*, but not *tbx18* (L'''). Scale bars in A-C,D-I: 50µm, L: 20µm, B': 10µm, L'-L''': 5µm. Colour channels in microscopy images were adjusted separately for brightness/contrast. A-C are projections, D-I,L are single optical sections. V=ventricle.

Figure S2.

A

Line sorted from	*Wildtype*	*Tg(tcf21:H2B-Dendra2)*	*Tg(tbx18:myr-eGFP)*	*Tg(wt1b:H2B-Dendra2)*	*Tg(myl7:eGFP)*	*Tg(kdrl:GFP;gata1a:DsRed)*	*Tg(kdrl:GFP;gata1a:DsRed)*	*Tg(tcf21:DsRed2; myl7:eGFP) x Tg(kdrl:GFP; gata1a:DsRed)*	*Empty well*
Fluorescent cells sorted	No	Yes	Yes	Yes	Yes	Yes *kdrl*:GFP	Yes *gata1a*:DsRed	No	No
Number of cells sequenced	52	228	52	50	14	15	2	48	3
Number of cells after QC	51	137	52	47	14	15	2	48	0
Proportion passed (%)	98	60	100	94	100	100	100	100	0

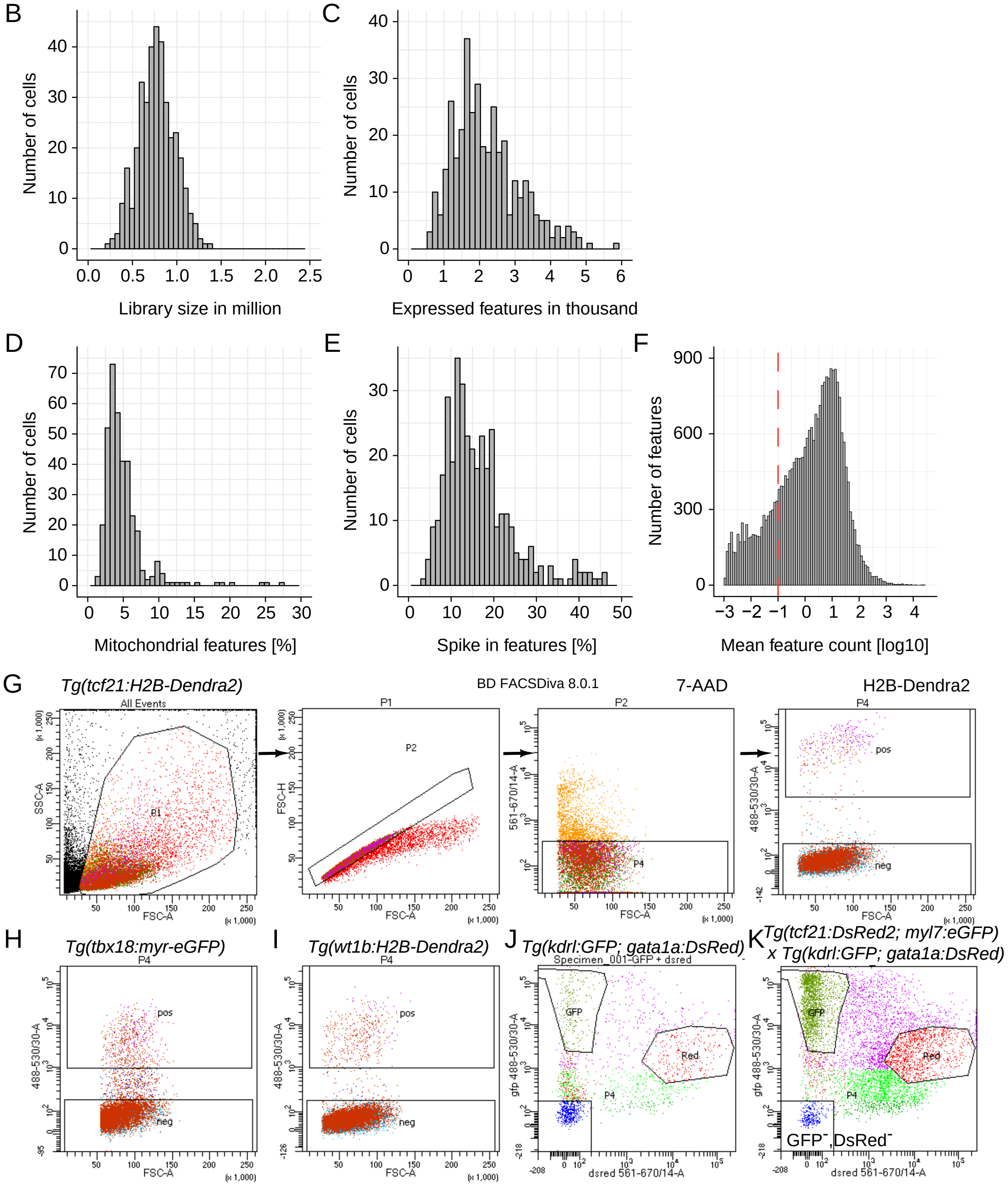

Figure S2. Quality control of the scRNA-seq dataset. Related to Figure 2.

(A) Number of single cells processed from each reporter/wildtype line to generate the scRNA-seq dataset. QC=quality control. (B-F) Histograms of library sizes (B), numbers of expressed features (C), percentage of mitochondrial reads on total reads (D), percentage of spike-in reads on total reads (E) and mean gene expression levels across cells (F). The dashed red line in F indicates the cut-off value of 0.1 read counts that was used to exclude genes with a lower mean expression level. (G) Representative FACS strategy to isolate fluorescent cells from reporter hearts (*TgBAC(tcf21:H2B-Dendra2)*). 7-AAD was used to exclude non-viable cells. (H-J) Flow cytometry profiles of *tbx18*:myr-eGFP^{+} (H), *wt1b*:H2B-Dendra2^{+} (I), *kdrl*:GFP/*gata1a*:DsRed^{+} (J) cells. (K) FACS gating of quadruple negative cells in *Tg(tcf21:DsRed2;myl7:eGFP)* x *Tg(kdrl:GFP;gata1a:DsRed)* hearts.

Figure S3.

A

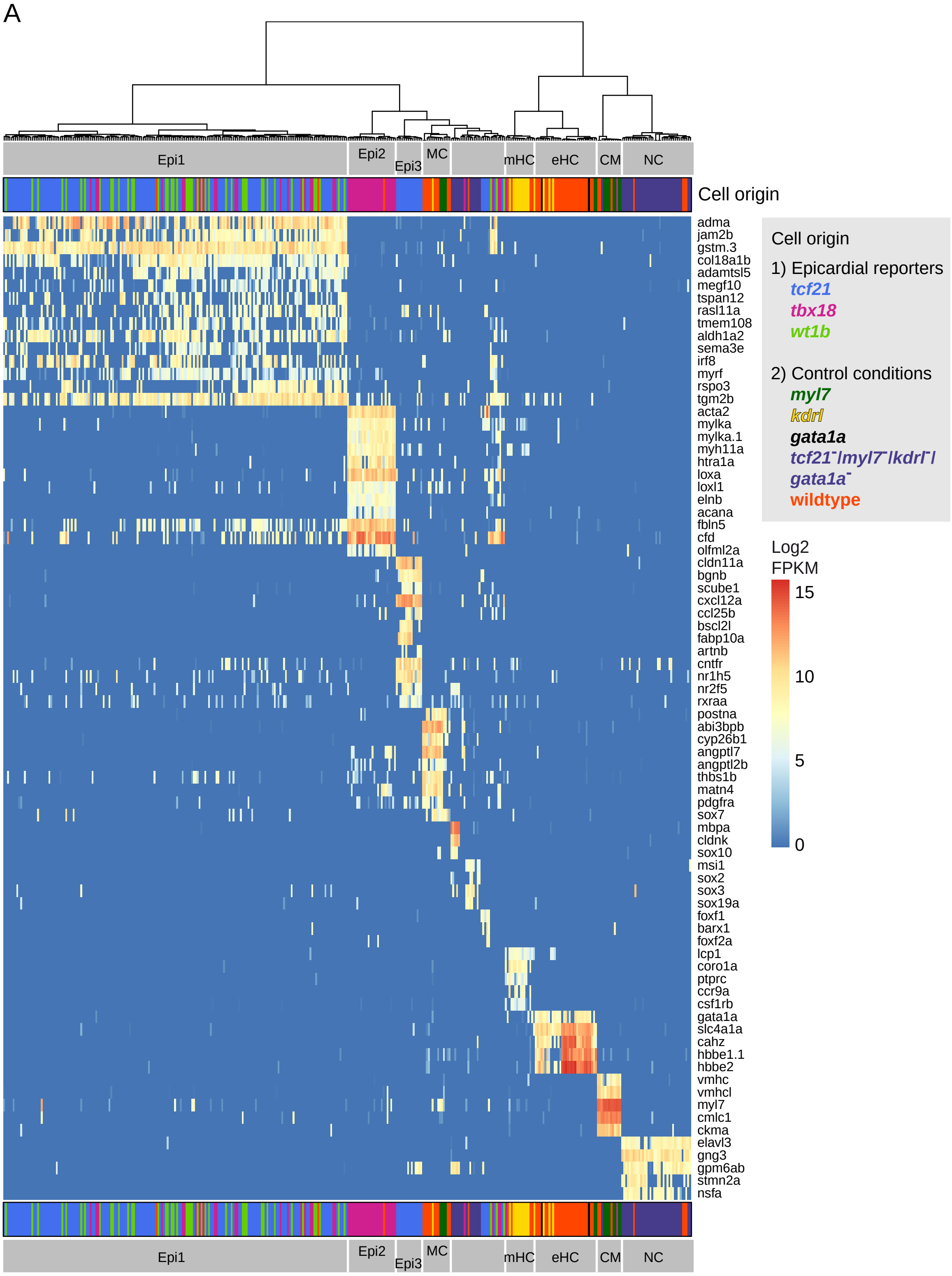
Epi1
Epi2
Epi3
MC
mHC
eHC
CM
NC
Cell origin
adma
jam2b
gstm.3
col18a1b
adamtsl5
megf10
tspan12
rasl11a
tmem108
aldh1a2
sema3e
irf8
myrf
rspo3
tgm2b
acta2
mylka
mylka.1
myh11a
htra1a
loxa
loxl1
elnb
acana
fbln5
cfd
olfml2a
cldn11a
bgnb
scube1
cxcl12a
ccl25b
bscl2l
fabp10a
artnb
cntfr
nr1h5
nr2f5
rxraa
postna
abi3bpb
cyp26b1
angptl7
angptl2b
thbs1b
matn4
pdgfra
sox7
mbpa
cldnk
sox10
msi1
sox2
sox3
sox19a
foxf1
barx1
foxf2a
lcp1
coro1a
ptprc
ccr9a
csf1rb
gata1a
slc4a1a
cahz
hbbe1.1
hbbe2
vmhc
vmhcl
myl7
cmlc1
ckma
elavl3
gng3
gpm6ab
stmn2a
nsfa
Cell origin
1) Epicardial reporters
tcf21
tbx18
wt1b
2) Control conditions
myl7
kdrl
gata1a
tcf21⁻/myl7⁻/kdrl⁻/
gata1a⁻
wildtype
Log2
FPKM
15
10
5
0

Figure S3. Marker gene expression across scRNA-seq clusters. Related to Figure 2.

(A) Cells were clustered in an unsupervised manner (columns). Shown are log2 transformed FPKM values. Cell cluster identity and colour-coded line origin of each cell are indicated at top and bottom of the heatmap. CM=cardiomyocytes, eHC=erythroid haematopoietic cells, mHC=myeloid haematopoietic cells, NC=neural cells, MC=mesenchymal cells.

Figure S4.

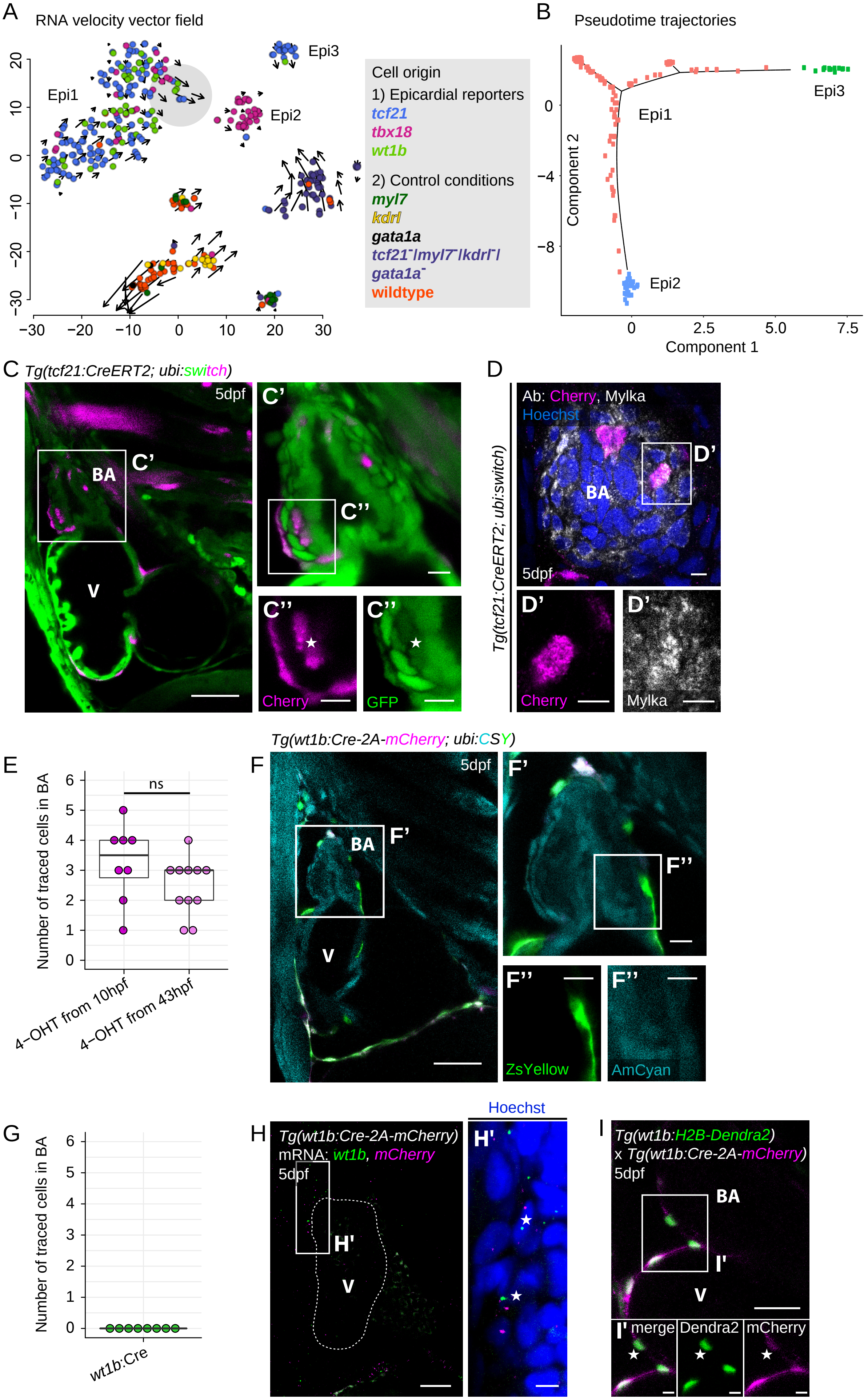
A
RNA velocity vector field
Epi1
Epi2
Epi3
Cell origin
1) Epicardial reporters
tcf21
tbx18
wt1b
2) Control conditions
myl7
kdrl
gata1a
tcf21⁻/myl7⁻/kdrl⁻/
gata1a⁻
wildtype
B
Pseudotime trajectories
Component 1
Component 2
C
Tg(tcf21:CreERT2; ubi:switch)
5dpf
BA
V
C'
C''
Cherry
GFP
D
Ab: Cherry, Mylka
Hoechst
Tg(tcf21:CreERT2; ubi:switch)
D'
Mylka
E
Number of traced cells in BA
ns
4-OHT from 10hpf
4-OHT from 43hpf
Tg(wt1b:Cre-2A-mCherry; ubi:CSY)
F
F'
F''
ZsYellow
AmCyan
G
wt1b:Cre
H
Tg(wt1b:Cre-2A-mCherry)
mRNA: wt1b, mCherry
H'
Hoechst
I
Tg(wt1b:H2B-Dendra2)
x Tg(wt1b:Cre-2A-mCherry)
I'
merge
Dendra2
mCherry

Figure S4. Epi2 cells have a *tcf21* origin but never express *wt1b*. Related to Figure 3.

(A) Directionality of cell differentiation in the scRNA-seq dataset, as determined by the Velocyto R package. mRNA velocities are shown in a vector field that is projected onto the pre-calculated t-SNE plot. A group of cells in Epi1 that differentiates towards Epi2 is highlighted by grey background. (B) Pseudotime trajectories of Epi1-3 cells. Cell affiliation to Epi1-3 is indicated by colour. (C) 4-hydroxy-tamoxifen (4-OHT) mediated permanent colour labeling (magenta) in *tcf21*:Cre-ERT2, *ubi*:switch larvae at 5dpf. 4-OHT was added at 10hpf. (C’,C’’) *tcf21* lineage traced cells in the BA (asterisk, boxed area in C). (D) Antibody staining of Mylka and mCherry in the BA of a 5dpf *tcf21*:Cre-ERT2, *ubi*:switch larvae. (D’) A *tcf21* lineage traced cell expressing Mylka (boxed area in D). (E) Absolute quantification of *tcf21*:Cre-ERT2 traced cells in the BA. Larvae were incubated with 4-OHT from either 10hpf or 43hpf and analysed at 5dpf. (F) Permanent colour labelling (green) in the heart region of a *wt1b*:Cre-2A-mCherry; *ubi*:CSY larva at 5dpf. (F’,F’’) Absence of *wt1b* lineage traced cells in the BA (boxed area in F). (G) Absolute quantification of the number of *wt1b*:Cre lineage traced cells within the BA. (H) mRNA staining of *wt1b* (magenta) and *mCherry* (green) in a *TgBAC(wt1b:Cre-2A-mCherry)* heart at 5dpf. (H‘) Nuclei (asterisks) surrounded by *wt1b* and *mCherry*. (I) Single optical section from a *TgBAC(wt1b:H2B-Dendra2)* x *TgBAC(wt1b:Cre-2A-mCherry)* heart at 5dpf. (I’) Overlap of *wt1b*:H2B-Dendra2 and *wt1b*:Cre-2A-mCherry (asterisk). Scale bars in C,F: 50µm, H,I: 20µm, C’,C’’,D,D’,F’,F’’: 10µm, H’,I’: 5µm. Colour channels in microscopy images were adjusted separately for brightness/contrast. C,D,F,H,I are single optical sections. Data represented as median, first and third quartiles (box). Significance was calculated using Welch’s t-

test. V=ventricle, BA=bulbus arteriosus, Ab=antibody. E: n(4-OHT 10hpf)=8, n(4-OHT 43hpf)=11. G: n=8.

Figure S5.

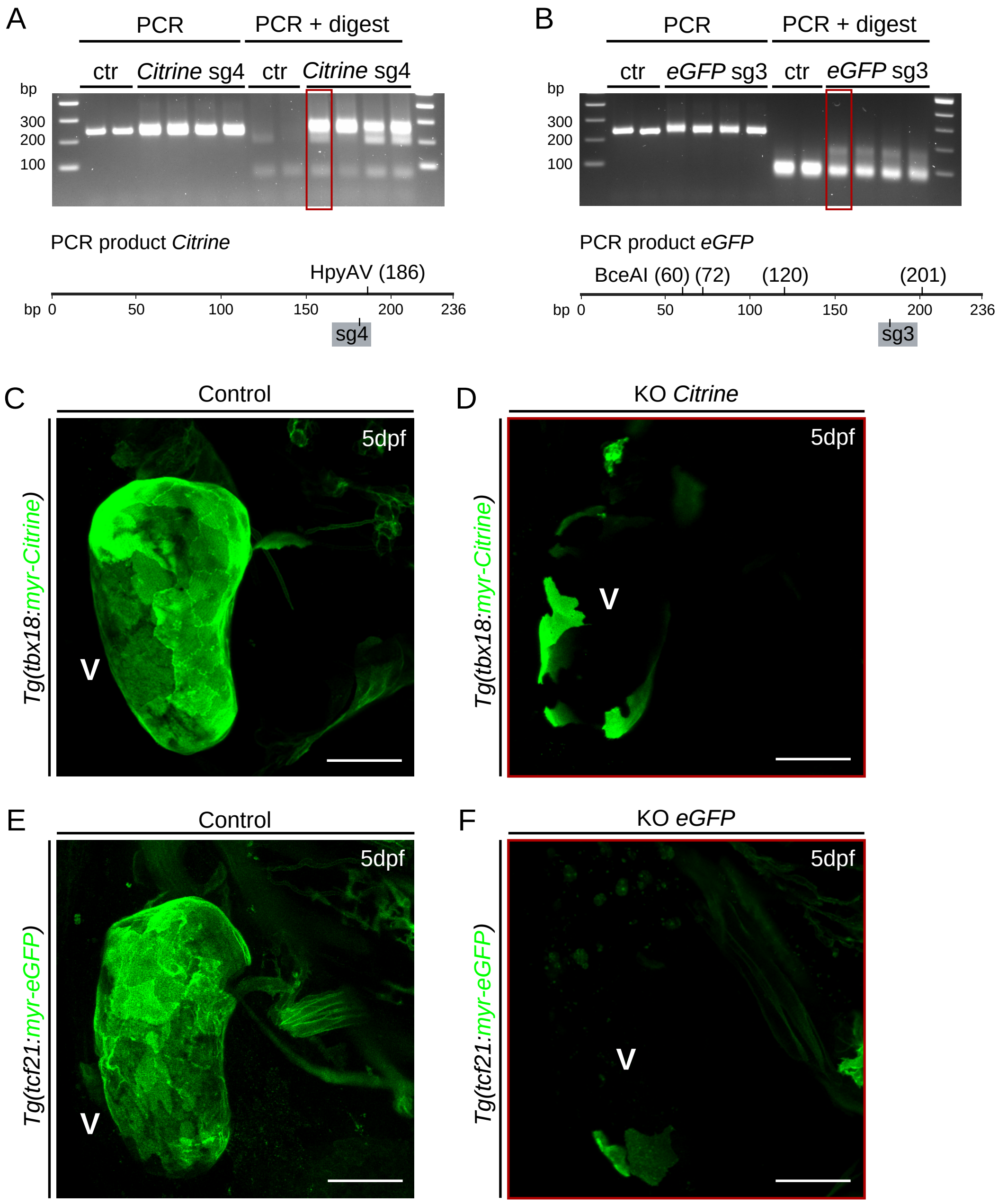
A
PCR
PCR + digest
ctr
Citrine sg4
ctr
Citrine sg4
bp
300
200
100
PCR product Citrine
HpyAV (186)
bp 0
50
100
150
200
236
sg4
B
PCR
PCR + digest
ctr
eGFP sg3
ctr
eGFP sg3
bp
300
200
100
PCR product eGFP
BceAI (60) (72)
(120)
(201)
bp 0
50
100
150
200
236
sg3
C
Control
Tg(tbx18:myr-Citrine)
5dpf
V
D
KO Citrine
Tg(tbx18:myr-Citrine)
5dpf
V
E
Control
Tg(tcf21:myr-eGFP)
5dpf
V
F
KO eGFP
Tg(tcf21:myr-eGFP)
5dpf
V

Figure S5. Efficient transient gene knockout by CRISPR/Cas9 technology. Related to Figures 4-6.

(A) Restriction digest to test the efficiency of sgRNA4 (sg4)/Cas9-mediated nuclease activity targeting *Citrine*. The sgRNA binding sequence is indicated by a grey square. PCR products were digested using HpyAV, presence of undigested PCR product indicates the disruption of the restriction site. DNA from the embryo analysed in D is highlighted by a red box. (B) Restriction digest to test the efficiency of sgRNA3 (sg3)/Cas9-mediated nuclease activity targeting *eGFP*. PCR products were digested using BceAI. DNA from the embryo analysed in F is highlighted by a red box. (C) Projection of the heart in a 5dpf *TgBAC(tbx18:myr-Citrine)*^{ox185} larva injected with control sgRNA. (D) Projection of the heart in a 5dpf larva injected with sg4 targeting *Citrine* (KO *Citrine*), showing strongly disrupted fluorescence. (E) Projection of the heart in a 5dpf *TgBAC(tcf21:myr-eGFP)*^{ox183} larva injected with control sgRNA. (F) Projection of the heart in a 5dpf larva injected with sg3 targeting *eGFP* (KO *eGFP*). Scale bars: 50µm. V=ventricle.

Figure S6.

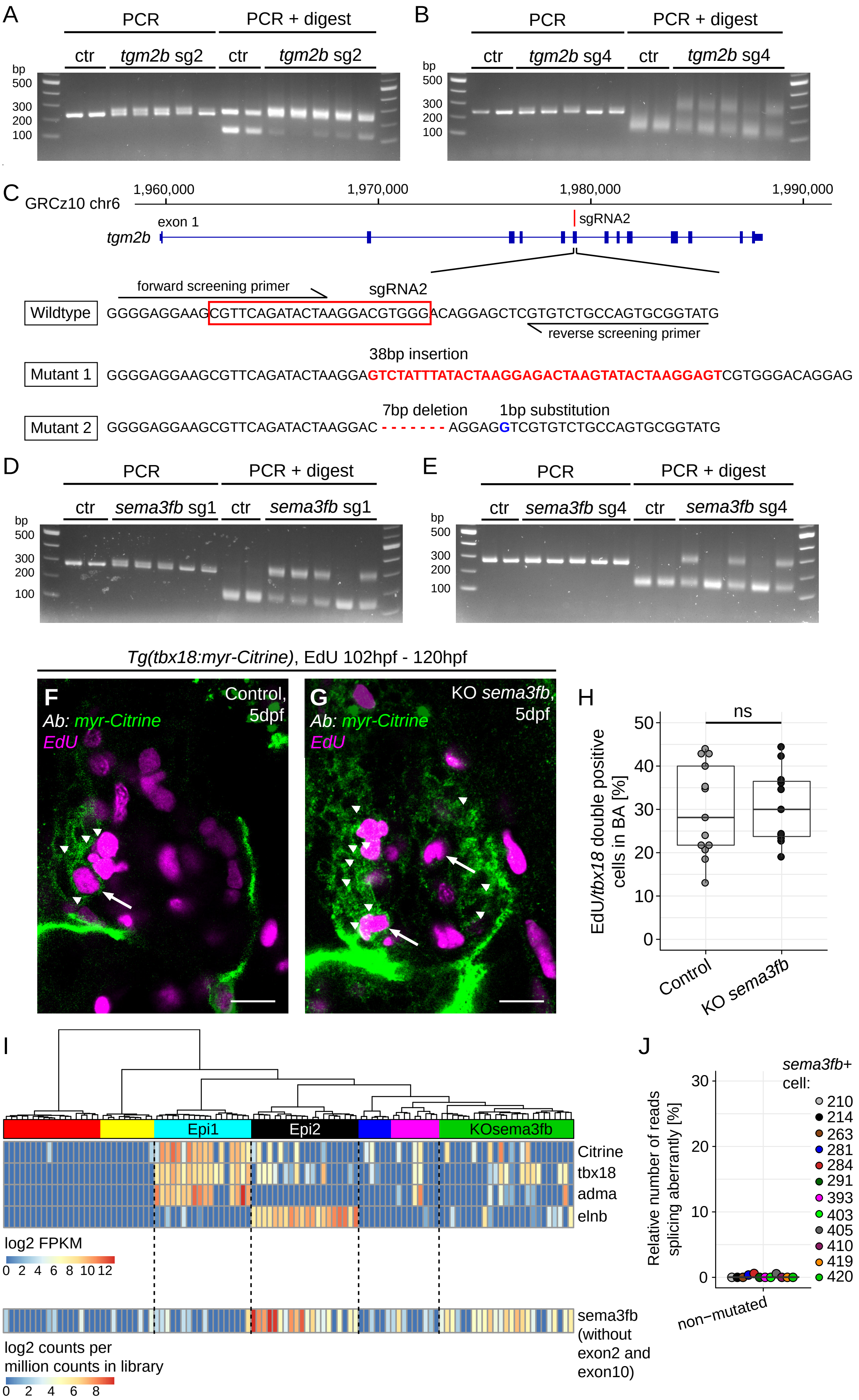
A
PCR
PCR + digest
ctr
tgm2b sg2
bp
500
300
200
100
B
tgm2b sg4
C
GRCz10 chr6
1,960,000
1,970,000
1,980,000
1,990,000
exon 1
sgRNA2
tgm2b
forward screening primer
sgRNA2
Wildtype
GGGGAGGAAGCGTTCAGATACTAAGGACGTGGGACAGGAGCTCGTGTCTGCCAGTGCGGTATG
reverse screening primer
38bp insertion
Mutant 1
GGGGAGGAAGCGTTCAGATACTAAGGAGTCTATTTATACTAAGGAGACTAAGTATACTAAGGAGTCGTGGGACAGGAG
7bp deletion
1bp substitution
Mutant 2
GGGGAGGAAGCGTTCAGATACTAAGGAC - - - - - - - AGGAGGTCGTGTCTGCCAGTGCGGTATG
D
sema3fb sg1
E
sema3fb sg4
Tg(tbx18:myr-Citrine), EdU 102hpf - 120hpf
F
Control, 5dpf
Ab: myr-Citrine
EdU
G
KO sema3fb, 5dpf
H
ns
EdU/tbx18 double positive cells in BA [%]
50
40
30
20
10
0
Control
KO sema3fb
I
Epi1
Epi2
KOsema3fb
Citrine
tbx18
adma
elnb
log2 FPKM
0 2 4 6 8 10 12
sema3fb (without exon2 and exon10)
log2 counts per million counts in library
0 2 4 6 8
J
sema3fb+ cell:
210
214
263
281
284
291
393
403
405
410
419
420
Relative number of reads splicing aberrantly [%]
30
20
10
0
non−mutated

Figure S6. Testing the efficiency of sgRNA/Cas9-mediated nuclease activity targeting *tgm2b* and *sema3fb*. Related to Figures 4 and 5.

(A) Restriction digest to test the efficiency of sgRNA2 (sg2)/Cas9-mediated nuclease activity targeting *tgm2b*. PCR products were digested using BmgBI. (B) Restriction digest to test the efficiency of sgRNA4 (sg4)/Cas9-mediated nuclease activity targeting *tgm2b*. PCR products were digested using HpyCH4III. (C) Schematic indicating the genomic binding site of *tgm2b* sgRNA2 (red box) and the gene editing events identified in the two stable *tgm2b* mutant lines. (D) Restriction digest to test the efficiency of sgRNA1 (sg1)/Cas9-mediated nuclease activity targeting *sema3fb*. PCR products were digested using BsrI. (E) Restriction digest to test the efficiency of sgRNA4 (sg4)/Cas9-mediated nuclease activity targeting *sema3fb*. PCR products were digested using Fnu4HI. (F-H) EdU detection and antibody staining against myr-Citrine in 5dpf *TgBAC(tbx18:myr-Citrine)*^{ox185} larvae. EdU/myr-Citrine^{+} cells in the BA are highlighted by arrows, myr-Citrine^{+} cells by arrowheads. (F) The BA in a control larva. (G) The BA in a KO *sema3fb* larva. (H) Relative quantification of the number of *tbx18*:myr-Citrine^{+} cells in the BA that were EdU^{+}. (I) Heatmap showing marker gene expression (log2 FPKM) in *sema3fb*-mutated single cells. The expression of *sema3fb* is shown as log2 of *sema3fb* read counts per million library counts. *sema3fb* counts were computed excluding exon 2 and exon 10 to ensure that only *sema3fb* cDNA excluding genomic amplicons was quantified. (J) Levels of abnormally splicing reads in the *sema3fb* locus in non *sema3fb* knockout cells, relative to the total number of splicing *sema3fb* reads. Colours indicate cell identity. Scale bars in F,G: 10µm. Colour channels in microscopy images were adjusted separately for brightness/contrast. F,G are single optical sections. Data represented as median, first and third quartiles (box).

Significance was calculated using Welch’s t-test. Ab=antibody, ns=non-significant. H: n(control)=13, n(KO *sema3fb*)=11.

Figure S7.

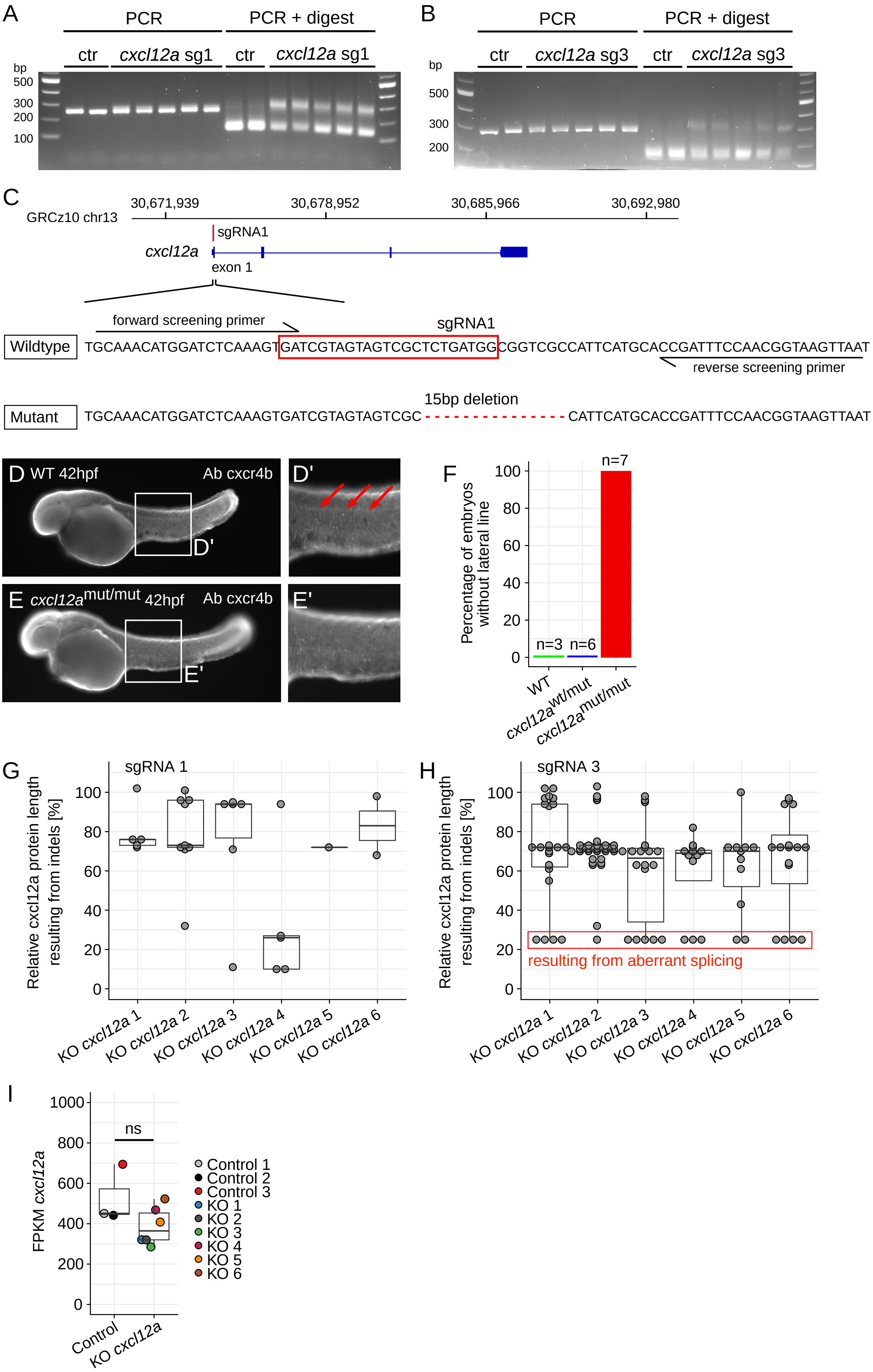
A
PCR
PCR + digest
ctr
cxcl12a sg1
ctr
cxcl12a sg1
bp
500
300
200
100
B
PCR
PCR + digest
ctr
cxcl12a sg3
ctr
cxcl12a sg3
bp
500
300
200
C
GRCz10 chr13
30,671,939
30,678,952
30,685,966
30,692,980
sgRNA1
cxcl12a
exon 1
forward screening primer
sgRNA1
Wildtype
TGCAAACATGGATCTCAAAGTGATCGTAGTAGTCGCTCTGATGGCGGTCGCCATTCATGCACCGATTTCCAACGGTAAGTTAAT
reverse screening primer
15bp deletion
Mutant
TGCAAACATGGATCTCAAAGTGATCGTAGTAGTCGC - - - - - - - - - - - - - - - CATTCATGCACCGATTTCCAACGGTAAGTTAAT
D WT 42hpf
Ab cxcr4b
D'
E cxcl12a mut/mut 42hpf
Ab cxcr4b
E'
F
Percentage of embryos without lateral line
n=7
n=3
n=6
WT
cxcl12a wt/mut
cxcl12a mut/mut
G
sgRNA 1
Relative cxcl12a protein length resulting from indels [%]
KO cxcl12a 1
KO cxcl12a 2
KO cxcl12a 3
KO cxcl12a 4
KO cxcl12a 5
KO cxcl12a 6
H
sgRNA 3
Relative cxcl12a protein length resulting from indels [%]
resulting from aberrant splicing
KO cxcl12a 1
KO cxcl12a 2
KO cxcl12a 3
KO cxcl12a 4
KO cxcl12a 5
KO cxcl12a 6
I
FPKM cxcl12a
ns
Control 1
Control 2
Control 3
KO 1
KO 2
KO 3
KO 4
KO 5
KO 6
Control
KO cxcl12a

Figure S7. Testing the efficiency of sgRNA/Cas9-mediated nuclease activity targeting *cxcl12a*. Related to Figure 6.

(A) Restriction digest to test the efficiency of sgRNA1 (sg1)/Cas9-mediated nuclease activity targeting *cxcl12a*. PCR products were digested using BccI. (B) Restriction digest to test the efficiency of sgRNA3 (sg3)/Cas9-mediated nuclease activity targeting *cxcl12a*. PCR products were digested using BsmFI. (C) Schematic indicating the genomic binding site of *cxcl12a* sgRNA1 (red box) and the 15bp deletion identified in the stable *cxcl12a* mutant line. (D-F) Analysis of lateral line development via antibody staining against cxcr4b in wildtype (D) and homozygous *cxcl12a* mutant (E) embryos at 42hpf. (F) Quantification of the relative number of embryos analysed in which the lateral line was not present. (G,H) Quantification of inferred cxcl12a protein lengths due to editing at sgRNA1 (G) or sgRNA3 (H) target sites, relative to wildtype cxcl12a. Protein length generated by splicing from exon1 into exon 3 is indicated by a red box in H. (I) FPKM values of *cxcl12a* in control and *cxcl12a*-mutated (KO) samples. D,E are stereo microscope images. F: Data represented as mean, G-I: Data represented as median, first and third quartiles (box). Significance was calculated using Welch's t-test.

Primer name	Primer sequence
BAC Tcf21 H2B-Dendra2 fkf for	CATCTCCTCAAGAAGTCCTTTTCTCCACTCCACCCTTGTCTCCAGCCAACatgccagagccagcgaagtctg
BAC Tcf21 myr-tdTomato / myr-eGFP fkf for	CATCTCCTCAAGAAGTCCTTTTCTCCACTCCACCCTTGTCTCCAGCCAACatgggctgcatcaagagcaagc
BAC Tcf21 fkf rev	CCAAACAACATTAGATTAACCGAATCGGAAAACCAAAATGAATTTATGAAAACTCAATATTAATTCTGATTGCAAGAAGTGTCTCACtccaGAAGTAGTGAGGAGGCTTT
BAC Tbx18 myr-Citrine / myr-eGFP fkf for	TTCTGGTGAACTTCTCTTTCTCGGCCAATCTGTCTTCTCGGTCGGTAACCatgggctgcatcaagagcaagc
BAC Tbx18 fkf rev	TACTTACGGGATTCGTCGCTGGTGCAATCTATCTCGCAACTCCTGGTGCTtccaGAAGTAGTGAGGAGGCTTT
BAC Wt1b H2B-Dendra2 fkf for	GTGTTTTGCAACCCAGAAAATCCGTCTAAATGCTGACAGAGCCGTGCGGCCCGatgccagagccagcgaagtctg
BAC Wt1b Cre-2A-Cherry fkf for	GTGTTTTGCAACCCAGAAAATCCGTCTAAATGCTGACAGAGCCGTGCGGCCCGATGCCcAAgAAAAAGcGcAAaG
BAC Wt1b fkf rev	GACCACATTGAGAGAGATTTTGAGGCGAGATTGTAAGGACGGGATGGTTTTCTCACtccaGAAGTAGTGAGGAGGCTTT

Table S1: Primers used for BAC recombineering. Related to Figure 1.

Gene/sgRNA name	sgRNA forward primer	sgRNA reverse primer
tgm2b sg2	ttcgCGTTCAGATACTAAGGACGT	aaacACGTCCTTAGTATCTGAACG
tgm2b sg4	ttcgGCTGCAGGAACTATCACTGT	aaacACAGTGATAGTTCCTGCAGC
sema3fb sg1	ttcgGGACAGTCTATGGCCAGTCC	aaacGGACTGGCCATAGACTGTCC
sema3fb sg4	ttcgTGACGGTGGTCACTGCTGCT	aaacAGCAGCAGTGACCACCGTCA
cxcl12a sg1	ttcgGATCGTAGTAGTCGCTCTGA	aaacTCAGAGCGACTACTACGATC
cxcl12a sg3	ttcgGCTCGCGAATGCTTCTCTGT	aaacACAGAGAAGCATTCGCGAGC
Citrine sg4	ttcgCTCGTGACCACCTTCGGCTA	aaacTAGCCGAAGGTGGTCACGAG
eGFP sg3	ttcgGCACTGCACGCCGTAGGTCA	aaacTGACCTACGGCGTGCAGTGC
mCherry sg1	ttcgCGGCCACGAGTTCGAGATCG	aaacCGATCTCGAACTCGTGGCCG
mCherry sg2	ttcgTCTGGGTGCCCTCGTAGGGG	aaacCCCCTACGAGGGCACCCAGA
AmCyan sg1	ttcgGAATGCAAGGGGCCCACCGT	aaacACGGTGGGCCCCTTGCATTC
AmCyan sg2	ttcgCTATCTACAGTGTTCATGTA	aaacTACATGAACACTGTAGATAG

Table S2: Primers used for Golden Gate cloning of sgRNA sequences into the U6a promoter Ac/Ds mini-vector. Related to Figures 4-6. sgRNA sequences (without PAM) are underlined.

Gene/ sgRNA name	PCR forward primer	PCR reverse primer	Product size (bp)
tgm2b sg2	CAGTAATGACCAGGATTCAGGG	gtgcatgtagcccATTAAGGAT	221
tgm2b sg4	ctgtgcaaaacatatgctggat	TGTGCACACCTTCACTTTTCTC	228
sema3fb sg1	ATTGCCCCACAAAATAACATTC	CAATCCTTAAAAGGAACGCAAG	273
sema3fb sg4	TTCTCACAGCTAACTTTTCCCG	TGTTGGAGATGGAAACAGACAC	259
cxcl12a sg1	AGCCTGTTCGCAAATTTAACTC	TCACCATAAAGTGTGCCAAAAC	245
cxcl12a sg3	TCATTATTCCATCCAACGTTCA	CCATTCAGTGTTTGGAGATTGA	284
Citrine, *eGFP*	GAGGAGCTGTTCACCGGG	AAGTCGTGCTGCTTCATGTG	236
mCherry sg1, sg2	AGGGCGAGGAGGATAACATG	TCAAGTAGTCGGGGATGTCG	254
AmCyan sg1, sg2	CCATATGGATGGCTGTGTCA	CATTCCGTCAGGAAATGCTT	231

Table S3: Primers used to amplify genomic regions containing sgRNA/Cas9 target sites. Related to Figures 4-6.

Primer name	Primer sequence	PCR product size [bp]
sema3fb mRNA for	ATTTGAGGCGAGCGAATGAGT	1669
sema3fb mRNA rev	ACCTCGTCTGGGTAGTCCTT	-
sema3fb-ISPCR cDNA for	aagcagtggtatcaacgcagagtATTTGAGGCGAGCGAATGAGT	1715
sema3fb-ISPCR cDNA rev	aagcagtggtatcaacgcagagtACCTCGTCTGGGTAGTCCTT	-
sema3fb gDNA sg1 for	CAGGGGAATTGCATGGTCCT	3683
sema3fb gDNA sg1 rev	AGTGGGGTTGGATGTCATGG	-
sema3fb gDNA sg4 for	ACTGAGCTGAAACAACCTCACTT	3104
sema3fb gDNA sg4 rev	ATTCTCAGTCTGTACGCTTGGCT	-
cxcl12a-ISPCR cDNA rev	aagcagtggtatcaacgcagagtGTGTGTTGTGACGCTGAGTTC	1395

Table S4. Primers used to amplify *sema3fb* cDNA and genomic regions in *sema3fb*-mutated single cells. Related to Figure 5.
